# Nanoparticle Therapeutics in Clinical Perspective: Classification, Marketed Products, and Regulatory Landscape

**DOI:** 10.1002/smll.202502315

**Published:** 2025-06-02

**Authors:** Nimeet Desai, Dhwani Rana, Mitali Patel, Neha Bajwa, Rajendra Prasad, Lalitkumar K. Vora

**Affiliations:** ^1^ Department of Eye and Vision Science, Institute of Life Course and Medical Sciences University of Liverpool Liverpool L7 8TX UK; ^2^ Department of Pharmaceutics National Institute of Pharmaceutical Education and Research‐Ahmedabad (NIPER‐A) Gandhinagar Gujarat 382355 India; ^3^ Maliba Pharmacy College Uka Tarsadia University Surat Gujarat 394350 India; ^4^ University Center of Excellence in Research Baba Farid University of Health Sciences Faridkot Punjab 151203 India; ^5^ School of Biochemical Engineering Indian Institute of Technology (BHU) Varanasi Uttar Pradesh 221005 India; ^6^ School of Pharmacy Queen's University Belfast 97 Lisburn Road Belfast BT9 7BL UK

**Keywords:** marketed products, nanomedicines, nanoparticles, regulatory approval, regulatory guidelines

## Abstract

Nanoparticle‐based therapeutics, emerging from advances in nanotechnology, outperform traditional drug therapies by virtue of their distinct biological properties that enhance therapeutic efficacy, reduce toxicity, and enable precise targeting. Since the 1980s, the number of nanoparticle‐based pharmaceutical products has expanded considerably, capturing a significant portion of the pharmaceutical market. These systems function as therapeutic agents or as vehicles for delivering active pharmaceutical or diagnostic compounds to targeted areas. However, despite their transformative potential, the development of comprehensive and harmonized regulatory frameworks for nanomedicines remains a critical challenge. This review provides a current overview of market‐approved nanoparticle therapeutics, analyzing global regulatory strategies, including pre‐clinical testing, safety assessments, manufacturing processes, and quality control standards. By discussing the existing shortcomings, this review highlights the importance of adaptive regulatory pathways in a global context. It aims to support researchers and stakeholders in navigating the regulatory landscape, facilitating the successful commercialization and clinical translation of nanoparticle‐based therapeutics.

## Graphical abstract

This review presents a detailed overview of clinically approved nanoparticle therapeutics, classifying them by type and discussing their unique advantages in drug delivery. It highlights regulatory challenges across global markets and emphasizes the need for adaptive approval pathways. A comparative analysis of nanoparticle classes and a survey of approved products offer insights into current successes and future directions.

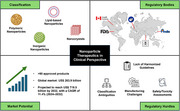

## 1 Introduction

Pharmaceutical research is advancing rapidly, particularly in understanding how drug delivery technologies influence therapeutic efficacy, patient outcomes, and economic viability. Major pharmaceutical companies are increasingly focused on developing drug delivery systems that integrate precision, convenience, and cost‐effectiveness.^[^ ^1^ ^]^ These efforts aim to achieve site‐specific delivery of active pharmaceutical ingredients, thereby improving clinical outcomes while minimizing off‐target effects and toxicity.^[^ ^2^ ^]^ However, conventional formulations such as tablets, capsules, and injectables continue to encounter major limitations, including poor solubility, nonspecific biodistribution, and heightened systemic toxicity.^[^ ^3^ ^]^ Nanomedicine has emerged as a promising solution to overcome these challenges by utilizing nanoscale carriers engineered for enhanced pharmacokinetics, controlled release, and targeted delivery to pathological sites.^[^ ^4^ ^]^

As the field of nanomedicine continues to expand, translating these advances into clinically approved therapies remains a complex endeavor, largely due to the unique physicochemical properties of nanoparticles and the absence of harmonized global regulatory guidelines.^[^ ^5^ ^]^ These complexities introduce uncertainties across preclinical evaluation, clinical trial design, and quality control processes. The development, approval, and commercialization of nanoparticle therapeutics demand comprehensive regulatory oversight to ensure safety, efficacy, and consistency in manufacturing, while also accommodating the scientific nuances specific to nanoscale formulations.^[^ ^6^ ^]^ Recent efforts to streamline this process include the publication of the DELIVER framework, which offers a structured roadmap addressing translational hurdles in nanomedicine development by integrating design principles, experimental rigor, regulatory considerations, and manufacturing challenges.^[^ ^7^ ^]^ While this framework marks an important step toward bridging the gap between research and clinical application, it primarily focuses on overarching strategies and prospective checkpoints to guide early‐phase researchers and developers. It provides high‐level principles but stops short of deeply analyzing existing regulatory precedents and implementation bottlenecks encountered in actual nanomedicine approvals.

In contrast, this review provides a regulatory‐centric analysis by synthesizing current global approval trends and proposing adaptive frameworks to support clinical translation. It consolidates data from market‐approved nanomedicines and investigates formulation‐specific regulatory considerations that influence development timelines and approval outcomes. Moreover, it critically examines the regulatory strategies and requirements adopted by key international agencies such as the US Food and Drug Administration (FDA), the European Medicines Agency (EMA), and Japan's Pharmaceuticals and Medical Devices Agency (PMDA), identifying how these institutions converge or diverge in their approaches to nanomedicine oversight. Beyond outlining the standard development pathway, this review also addresses contextual barriers that often complicate implementation. These include inconsistencies in manufacturing scalability, challenges in ensuring reproducibility of complex nanosystems, and the evolving landscape of post‐marketing surveillance obligations. By spotlighting these under‐discussed aspects, the review aims to complement existing translational frameworks with practical, evidence‐based insights. In doing so, it seeks to inform researchers, industrial stakeholders, and regulatory bodies working to align scientific innovation with clinical accessibility in the nanomedicine domain.

## 2 Nanoparticle Types and Classification

Nanoparticle size and properties are pivotal in revolutionizing drug delivery systems, enabling precise adjustments to therapeutic efficacy and safety. Nanomedicine solutions have made a significant market impact, offering innovative therapeutic options. Particularly, sub‐200 nm nanoparticles exhibit exceptional tissue targeting, enhancing treatment effectiveness and minimizing adverse reactions.^[^ ^8^, ^9^ ^]^ Targeting tactics in nanoparticle delivery are classified as passive and active. Passive targeting exploits the enhanced permeability and retention effect, directing nanoparticles to accumulate at diseased sites with compromised blood vessels.^[^ ^10^ ^]^ Active targeting involves surface modifications using ligands that bind to specific cellular receptors, guiding nanoparticles to disease‐specific areas like inflamed tissues or infected cells.^[^ ^11^ ^]^ Imaging contrast agents can complement drug delivery by enabling real‐time monitoring of distribution, target accumulation, and therapeutic efficacy.^[^ ^12^ ^]^ Utilizing nanoparticles offers a notable advantage due to their larger surface area compared to micron‐scale counterparts, enhancing solution transfer coefficients.^[^ ^13^ ^]^ Systems below 200 nm demonstrate high saturation solubility as per the Ostwald‐Freundlich equation, facilitating faster dissolution and improved medication absorption by the body. This attribute is particularly valuable for new medications, which often exhibit increased lipophilicity, molecular weight, and limited water solubility.^[^ ^14^ ^]^

**Figure 1:**
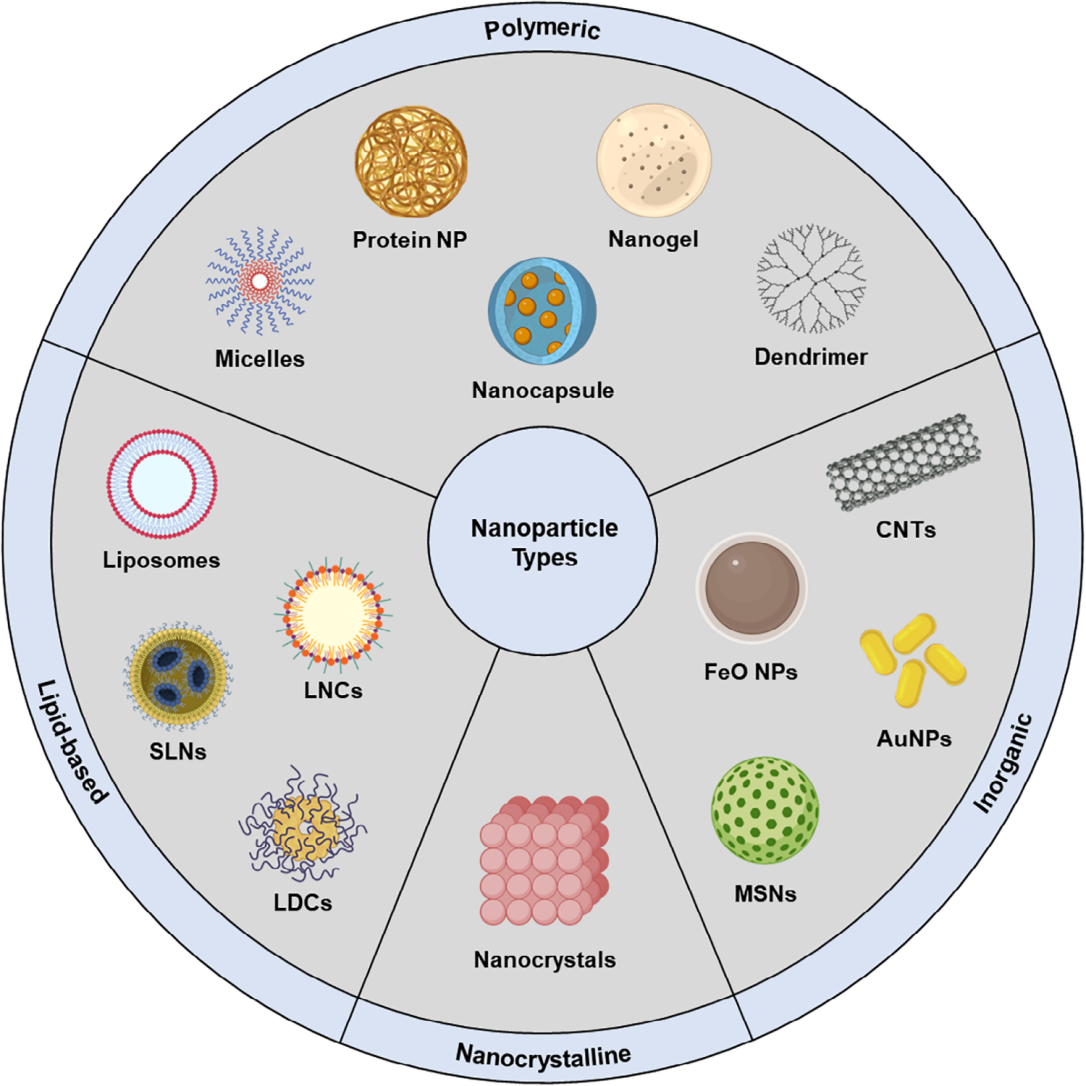
Schematic representation of different classes of nanoparticles.

Due to the sector's rapid expansion, it's crucial to establish a robust classification system for nanoparticles. This section offers a systematic evaluation of nanoparticles, grouping them into four main categories based on composition: lipid‐based, polymeric, inorganic, and nanocrystalline (**Figure** **1**). The aim is to provide a thorough understanding of the unique characteristics of each class and their potential roles in advancing drug delivery techniques.

### 2.1 Lipid‐Based Nanoparticles

This class of nanoparticles leverages lipids' amphiphilic structure (hydrophilic heads, hydrophobic tails) to form carriers such as liposomes, solid lipid nanoparticles (SLNs), and nanostructured lipid carriers (NLCs).^[^ ^15^ ^]^ They encapsulate both hydrophilic and hydrophobic drugs for tailored delivery, while benefiting from lipids' inherent biocompatibility and biodegradability, which reduces adverse effects and immunogenicity.^[^ ^16^ ^]^ By eliminating harmful solubilizing agents and enabling large‐scale production, these systems accelerate the clinical translation of novel therapies^[^ ^17^ ^]^ Various lipid nanoparticle designs can be customized to achieve specific therapeutic goals.

#### 2.1.1 Liposomes

**Figure 2:**
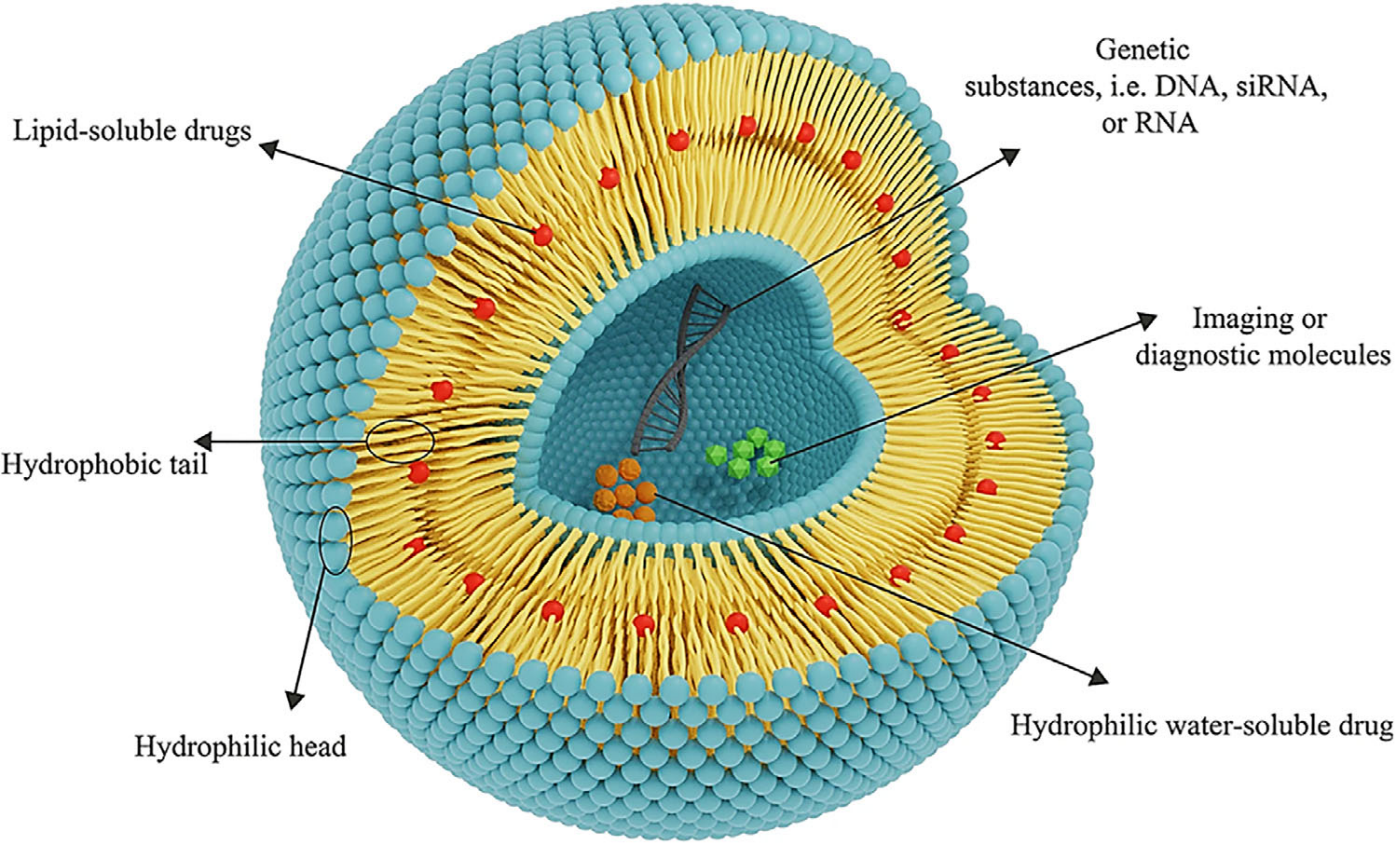
The general structure of the liposomes. Adapted with permission from ref. [28], Copyright Springer Nature 2021.

Liposomes are lipid–based spherical vesicles with aqueous cores enclosed by self‐assembled amphiphilic bilayers (hydrophilic heads facing water, hydrophobic tails inward).^[^ ^18^ ^]^ These nanocarriers facilitate precise, controlled release of diverse therapeutics, revolutionizing interventions (**Figure** **2**).^[^ ^19^ ^]^ They exist as multilamellar, small unilamellar, or large unilamellar vesicles depending on preparation (lipid film hydration, reverse‐phase evaporation, or ether injection), which determines size, stability, and encapsulation efficiency.^[^ ^20^, ^21^ ^]^ Size can be fine‐tuned via extrusion, sonication, or microfluidics to optimize release kinetics and targeting.^[^ ^22^ ^]^ Modifying lipid composition, such as adding cholesterol, adjusts membrane rigidity and stability,^[^ ^23^, ^24^ ^]^ while charged lipids tailor surface charge and biological interactions. Surface functionalization by ligand conjugation targets disease sites through ligand‐receptor interactions (e.g., antibody‐antigen binding),^[^ ^25^, ^26^ ^]^ and PEGylation extends circulation time, reduces immune clearance, and improves pharmacokinetics.^[^ ^27^ ^]^

#### 2.1.2 Solid Lipid Nanoparticles (SLNs)

**Figure 3:**
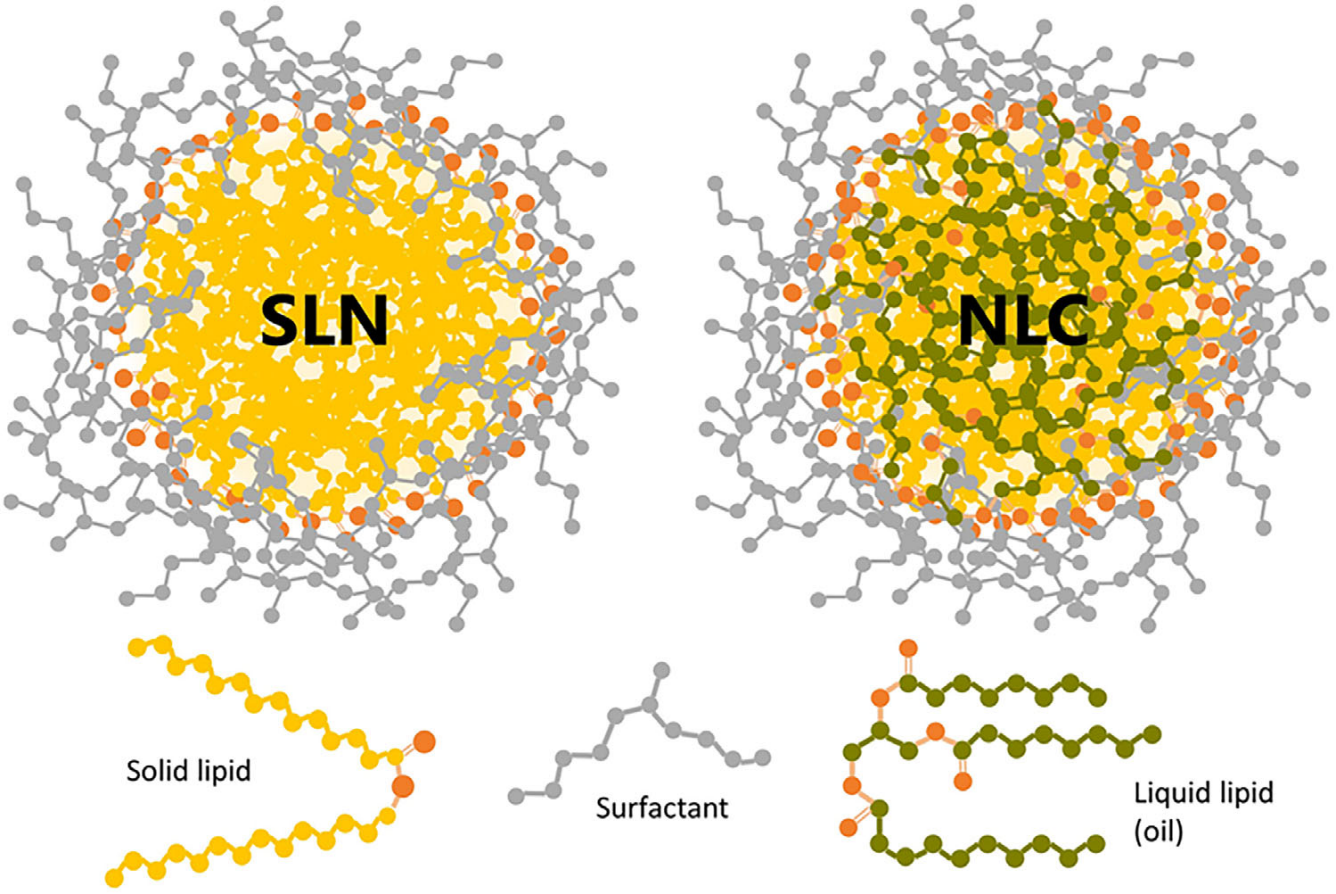
Schematic illustration comparing the structures of SLN, made entirely of solid lipids, and NLC, which contain both solid and liquid lipids. Both are stabilized by a neutral surfactant depicted in gray. Adapted with permission from ref. [47], Copyright Frontiers Media S.A. 2020.

SLNs, originally called lipospheres, emerged in the 1990s as biocompatible, biodegradable drug carriers with a solid lipid matrix (10–1000 nm) dispersed in aqueous or nonaqueous phases (**Figure** **3**).^[^ ^29^ ^]^ Stabilized by surfactants and composed of triglycerides, glyceride mixtures, or waxes, these particles remain solid at room and body temperature,^[^ ^30^ ^]^ reducing drug mobility to enhance controlled release and minimize leakage compared to liposomes.^[^ ^31^ ^]^ SLNs can encapsulate small and large drug molecules, genetic material, and vaccines, and can be dried into powders for capsule or tablet formulations.^[^ ^32^ ^]^ High‐pressure homogenization (HPH) melts a lipid‐surfactant‐drug mixture^[^ ^33^ ^]^ and forces it through narrow‐gap valves under high pressure to create nanodroplets that solidify into SLNs;^[^ ^34^ ^]^ tuning passes and pressure controls particle size and stability. Alternative fabrication methods include microemulsion, membrane contactor, phase inversion temperature, and coacervation, while spray drying or lyophilization improves stability and injectability.^[^ ^35^ ^]^ SLN classification depends on lipid composition, surfactants, and drug solubility, with three types identified.^[^ ^36^ ^]^ Type I SLNs feature a homogeneous matrix with controlled drug release. Type II SLNs have a drug‐free lipid core surrounded by a lipid‐drug shell due to lower drug load. Type III SLNs exhibit drug super‐saturation in the lipid matrix, enabling prolonged release.^[^ ^37^ ^]^ These formulations enhance drug stability against photochemical, oxidative, and chemical degradation,^[^ ^38^ ^]^ improve bioavailability of poorly water‐soluble compounds, and offer scalable, GMP‐compliant, cost‐effective production.^[^ ^39^ ^]^

#### 2.1.3 Nanostructured Lipid Carriers (NLCs)

NLCs combine solid and liquid lipids to form an amorphous solid matrix at both physiological and room temperatures. Unlike SLNs, NLCs include liquid lipids, preventing the formation of well‐defined lipid crystals (Figure 3). This enhances drug‐loading capacity and reduces particle size for more efficient drug delivery.^[^ ^40^ ^]^ The liquid lipid component also minimizes gelation and drug leakage during storage, ensuring long‐term stability. These carriers share with SLNs low toxicity, biodegradability, drug protection, controlled release kinetics, and a solvent‐free production process.^[^ ^41^ ^]^ Three NLC types are distinguished by lipid composition and fabrication. Type I replaces part of the solid lipids with liquid lipids, creating an imperfect crystal matrix that accommodates drugs without risk of expulsion.^[^ ^42^ ^]^ Type II retains the solid lipid ‐ polymorph combined with liquid lipids in an amorphous core to prevent crystallization and improve drug retention. Type III uses a water‐in‐oil‐in‐water emulsion to disperse small oil droplets in a solid lipid matrix, increasing loading and stability of oil‐soluble drugs.^[^ ^43^ ^]^ Typical NLC formulations blend solid and liquid lipids at ratios from 70:30 to 99.9:0.1 with surfactant concentrations of 0.5% to 5%,^[^ ^44^ ^]^ using common lipids such as stearic acid, glyceryl monostearate, carnauba wax, cetyl palmitate, and glyceryl palmitostearate together with oils like soybean, corn oil, squalene, medium‐chain triglycerides, and caprylic/capric triglycerides.^[^ ^45^ ^]^ Preparation methods mirror those for SLNs, including high‐pressure homogenization, emulsification ultrasonication, film ultrasonication, and solvent diffusion, with adjustments to accommodate the liquid lipid phase.^[^ ^46^ ^]^

#### 2.1.4 Other Lipid‐Based Nanoparticles

Liposomes, SLNs, and NLCs have demonstrated significant advancements in clinical translation. However, beyond these established lipid‐based nanosystems, several alternative lipid‐centric platforms hold substantial promise for clinical applications. One such platform is Lipid Nanocapsules (LNCs), self‐assembled nanoscale structures with an oily core enveloped by a sturdy shell, resembling a fusion of liposomes and traditional polymeric nanoparticles.^[^ ^48^ ^]^ LNCs consist of three main components: oils, a lipophilic surfactant like lecithin, and a non‐ionic surfactant such as Solutol. These components are FDA‐approved for various administration routes, ensuring suitability for medical applications.^[^ ^49^ ^]^ The oily core serves as both a drug reservoir and a penetration enhancer, employing medium‐chain triglycerides like capric and caprylic acid triglycerides (Labrafac). Lecithin concentration influences shell rigidity, while non‐ionic surfactants induce emulsion phase inversion, affecting particle size.^[^ ^50^ ^]^ LNC preparation typically employs the phase inversion temperature method, producing uniform particles through controlled heating‐cooling cycles.^[^ ^51^ ^]^ A recent innovation introduces a low‐temperature technique for LNC fabrication, which is advantageous for thermolabile pharmaceuticals because it avoids high temperatures. This method enables high co‐encapsulation of multiple drugs. These LNCs offer notable stability, adjustable release profiles, and adaptability for surface modification using PEGylated lipids or cationic lipids.^[^ ^52^ ^]^

Besides LNCs, lipid‐drug conjugates (LDCs) have also been extensively explored by the research community. LDCs entail covalently linking a therapeutic drug compound with a lipid molecule, aiming to enhance drug solubility and bioavailability for hydrophobic drugs, address challenges like poor absorption or rapid clearance, and achieve targeted tissue or cellular delivery.^[^ ^53^ ^]^ Typically, the drug is chemically bonded to a lipid moiety, which can encompass fatty acids, phospholipids, glycolipids, or other derivatives, chosen based on compatibility, desired release kinetics, and delivery requisites.^[^ ^54^ ^]^ This linkage offers multiple advantages: lipids facilitate drug solubilization, promoting aqueous dispersion; they confer stability, extending drug shelf life; and they can alter pharmacokinetics, prolonging circulation time, augmenting bioavailability, and enabling tissue/cellular targeting.^[^ ^55^ ^]^ The efficacy of an LDCs hinges on its conjugation chemistry, with options like esterification, amide bond formation, click chemistry, or functional group interactions selected based on the drug‐lipid characteristics and desired properties of the conjugate.^[^ ^56^ ^]^

Other novel lipid‐based nanosystems include exosomes and cell membrane‐functionalized nanoparticles. Exosomes, small extracellular vesicles typically sized from 30 to 150 nanometers, serve vital roles in intercellular communication and cargo transport. They transfer proteins, nucleic acids, and lipids to regulate various physiological and pathological processes.^[^ ^57^ ^]^ Exploiting their innate capacity to navigate biological barriers, such as the blood‐brain barrier, exosomes promise to be effective vehicles for drug delivery. Their natural biocompatibility and minimal immunogenicity make them advantageous for delivering therapeutic agents, including small molecules, proteins, nucleic acids (e.g., siRNA or miRNA), and gene‐editing tools like CRISPR‐Cas9, to specific cells or tissues.^[^ ^58^ ^]^

The process of utilizing exosomes for drug delivery involves several key steps. First, exosomes are isolated from donor cells (or biofluids), such as stem cells or immune cells, either through conventional ultracentrifugation‐based methods or newer techniques like size exclusion chromatography or polymer‐based precipitation.^[^ ^59^, ^60^ ^]^ These isolation methods help ensure the purity and quality of the exosome preparation. Then, exosomes are engineered to carry therapeutic cargo by leveraging their natural ability to incorporate biomolecules.^[^ ^61^ ^]^ This is done by treating donor cells with therapeutic agents or directly loading exosomes with drugs through methods like electroporation or sonication. Upon administration, exosomes navigate through the bloodstream and are internalized by target cells, wherein their cargo is released and exerts its therapeutic effect. The natural propensity of exosomes to fuse with the cell membrane aids in cargo delivery into the cytoplasm, where it can influence cellular processes.^[^ ^62^ ^]^ Exosomes, which are being clinically trialed for drug delivery applications, have also found commercial use in diagnostic products, highlighting their dual significance in advancing both targeted therapeutics and medical diagnostics.^[^ ^63^ ^]^

Cell membrane‐functionalized nanoparticles represent an emerging approach in drug delivery, utilizing lipids, or lipid membranes from diverse cell types to construct nanocarriers with biomimetic attributes.^[^ ^64^ ^]^ These nanoparticles consist of a synthetic or inorganic core enshrouded by cell‐derived membranes, with well‐established examples including red blood cells, white blood cells, platelets, cancer cells, and stem cell membrane‐coated nanoparticles. These biomimetic systems harness the distinct functionalities of cell sources for targeted applications.^[^ ^65^ ^]^ Red blood cell‐coated nanoparticles featuring CD47 proteins for extended circulation excel in drug delivery and imaging.^[^ ^66^ ^]^ White blood cell‐coated nanoparticles exhibit immune‐like traits, penetrating inflamed tissues and targeting tumors. Platelet‐coated nanoparticles offer hemostatic properties and wound‐healing potential.^[^ ^67^ ^]^ Cancer cell‐coated nanoparticles leverage cancer cell characteristics for tumor‐specific targeting, while stem cell‐coated nanoparticles tap into regenerative attributes for tissue engineering.^[^ ^68^ ^]^ These biomimetic nanoparticles hold immense promise for overcoming biological barriers and enhancing therapeutic outcomes in drug delivery, diagnostics, and regenerative medicine. The fabrication process involves cell membrane extraction, purification, and nanoparticle coating, necessitating careful preservation of functional membrane proteins.^[^ ^69^, ^70^ ^]^

### 2.2 Polymeric Nanoparticles

Polymeric nanoparticles are versatile 1–1000 nm carriers composed of biocompatible polymers that encapsulate or bind drugs for therapeutic, imaging, and diagnostic applications.^[^ ^71^ ^]^ Three main polymer categories exist: natural polymers (proteins such as albumin and gelatin, polysaccharides such as chitosan and alginate, and nucleic acids such as DNA), which provide biocompatibility, low immunogenicity, and biomimicry;^[^ ^72^ ^]^ synthetic polymers such as polyethylene glycol (PEG), poly(lactic‐co‐glycolic acid) (PLGA), and poly(ethyleneimine) (PEI), which enable precise control over nanoparticle size, shape, and release kinetics;^[^ ^73^ ^]^ and hybrid polymers combining natural and synthetic elements, as exemplified by Pluronic‐conjugated pharmapolymers, which enhance stability and deliver stimuli‐responsive release.^[^ ^74^ ^]^ The straightforward synthesis of these nanoparticles affords tight regulation of size, drug loading, and surface characteristics, resulting in structures that resist degradation, avoid premature drug release, achieve prolonged circulation, and allow adjustable release profiles to improve therapeutic outcomes and reduce side effects.^[^ ^75^ ^]^ Below are key classes of polymeric nanoparticles.

#### 2.2.1 Polymeric Nanosphere/Nanocapsules

**Figure 4:**
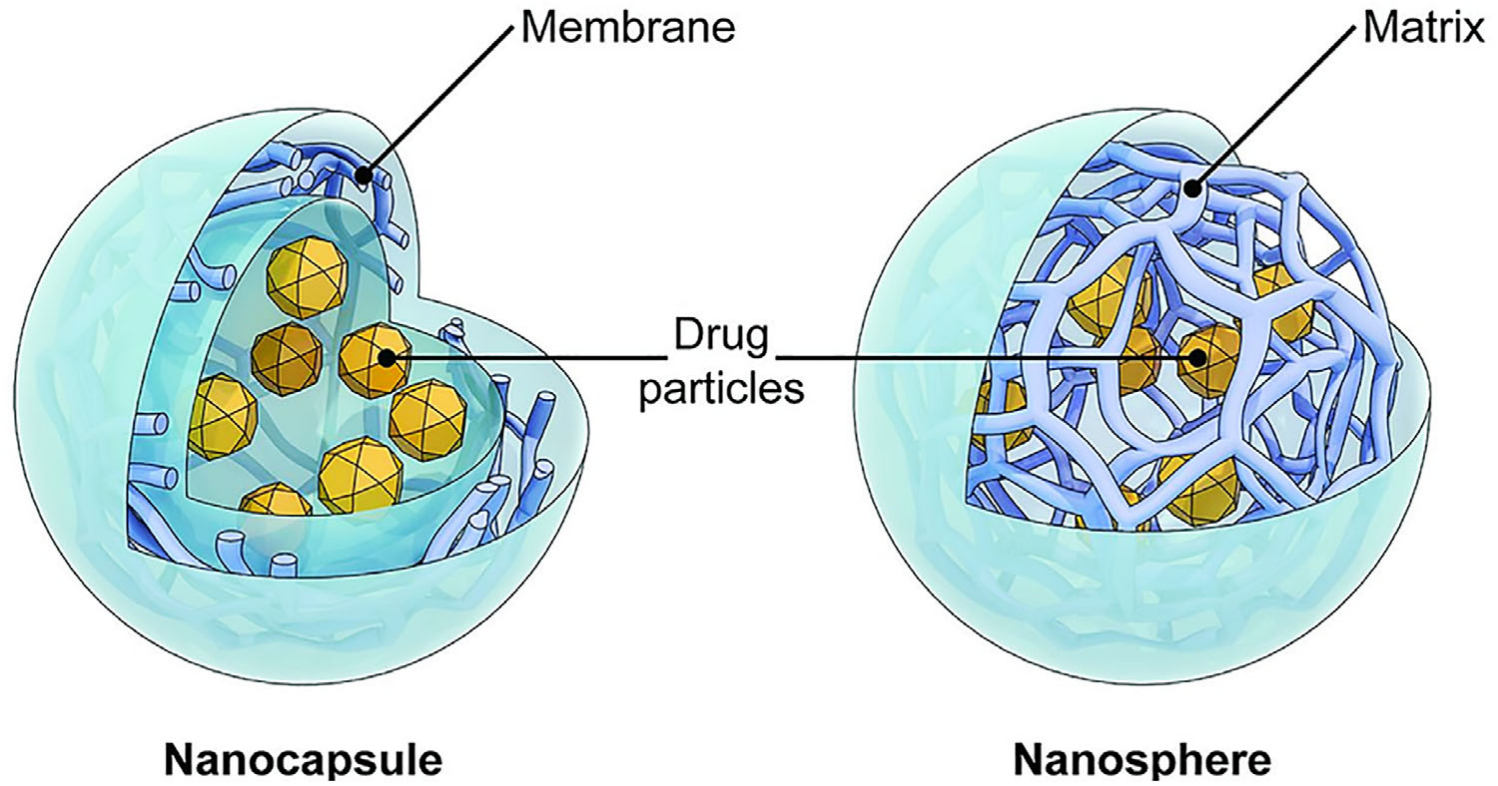
Diagrammatic depiction of the structural design of a nanocapsule compared to a nanosphere. The image is in the public domain and credited to the National Institutes of Health/Department of Health and Human Services.

Polymeric nanospheres and nanocapsules represent the most fundamental iterations of polymer‐based nanoparticles (**Figure** **4**). Nanospheres exhibit a monolithic structure, within which drugs are either dispersed, adsorbed onto surfaces, or encapsulated internally.^[^ ^76^ ^]^ Conversely, nanocapsules manifest as a vesicle‐like system wherein the drug resides within an inner liquid core, enveloped by a polymeric membrane. The active substance is generally dissolved within the inner core, though surface adsorption is also feasible.^[^ ^77^ ^]^

Making polymeric nanospheres involves methodologies yielding solid, spherical nanoparticles devoid of a distinct core‐shell arrangement.^[^ ^78^ ^]^ Preferred techniques encompass emulsion/solvent evaporation (wherein a polymer dissolved in a solvent is emulsified into another immiscible solvent, followed by solvent evaporation to induce nanosphere precipitation), nanoprecipitation (involving rapid mixing of a polymer solution with a nonsolvent to prompt polymer precipitation and nanosphere formation), and emulsion/solvent diffusion (where controlled solvent diffusion into a polymer solution induces nanosphere creation).^[^ ^79^ ^]^ Polymeric nanocapsules entail methods for establishing a core‐shell structure. Common approaches encompass interfacial polymerization (where polymerization of monomers at the interface of immiscible phases engenders a polymer shell encasing a core), template‐assisted synthesis (where the polymer is layered onto a core particle template, creating a core‐shell architecture), and coacervation (which induces phase separation via pH or temperature manipulation in a polymer solution to yield a polymer‐rich phase encapsulating the core material).^[^ ^80^ ^]^

Drug loading for both systems can be achieved through incorporation (during nanoparticle production) or incubation (adsorption post‐nanoparticle formation), although the former yields better loading efficiency. In addition, several loading enhancement methods are reported, which vary based on preparation techniques, additives used, and drug‐polymer properties.^[^ ^81^ ^]^ The drug release profile for both systems hinges on particulate system cross‐linking, morphology, size, density, drug properties, and several external factors (like pH, polarity, and presence of enzymes in dissolution media).^[^ ^82^ ^]^ Drug release from polymeric nanospheres/nanocapsules involves surface dissolution, where surface‐bound drug molecules dissolve rapidly; diffusion through the polymer matrix, as drug molecules move through the nanoparticle's structure; and erosion‐induced release, as the polymer matrix degrades, leading to particle disintegration and drug discharge.^[^ ^83^ ^]^

#### 2.2.2 Dendrimers

Dendrimers are hyperbranched, globular macromolecules (1–100 nm) with low polydispersity, built around a central core that anchors recurring branching units forming internal shells, and terminating in functional groups at the periphery.^[^ ^84^, ^85^ ^]^ These layers create internal voids that enhance structural stability and provide sites for guest molecule encapsulation.^[^ ^86^, ^87^ ^]^ By adjusting the number and length of dendron branches and the core's architecture, one can precisely control dendrimer size, cavity formation, and functional versatility for targeted delivery applications.^[^ ^88^ ^]^ The preparation of dendrimers involves diverse methodologies, each bearing distinct merits and considerations:

- Divergent Growth: This strategy originates from the core and extends outward, promoting branching growth. However, it may result in incomplete reactions and structural anomalies due to partial surface functionalization.^[^ ^89^ ^]^
- Convergent Growth: Initiated from the periphery and progressing toward the core, this method involves attaching surface units with additional monomers. While it enables rapid synthesis, purification challenges and limitations to low‐generation dendrimers are noted.^[^ ^90^ ^]^
- “Hypercores” and “Branched Monomers” Growth: This hybrid approach amalgamates elements of divergent and convergent methods, involving the assembly of oligomers before their attachment to the core.^[^ ^91^ ^]^
- “Double Exponential” Growth: Utilizing a double exponential function, this technique regulates the number of repeat units per dendrimer generation, allowing precise growth control.^[^ ^92^ ^]^
- “Lego” Chemistry: Employing highly functional monomers and groups, this method offers advantages like straightforward purification and environmentally friendly by‐products.^[^ ^93^ ^]^
- Click Chemistry: This method entails linking smaller units using heteroatoms, yielding dendrimers with a range of peripheral groups and distinct functionalities.^[^ ^94^ ^]^

**Figure 5:**
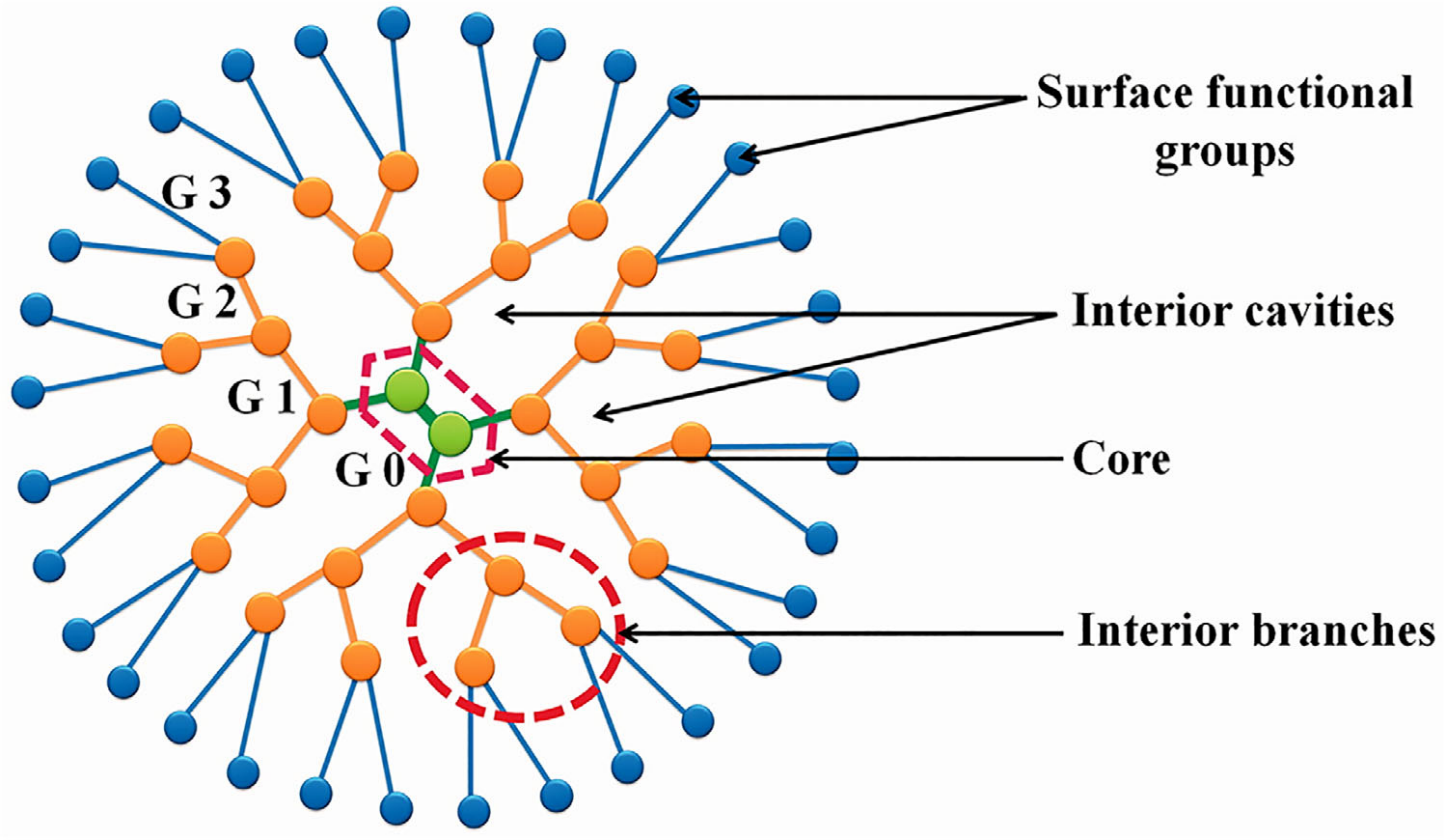
Typical architecture of dendrimers. Adapted with permission from ref. [100], Copyright Taylor & Francis 2016.

Dendrimers predominantly adopt two primary shapes: ellipsoidal and spherical.^[^ ^95^ ^]^ The resulting morphology is influenced by factors such as the initiator component; for instance, ethylenediamine‐based dendrimers frequently assume an ellipsoidal form. This shape significantly dictates the spatial distribution of functional groups on the dendrimer's surface and interior.^[^ ^96^ ^]^ Dendrimers exhibit generation‐dependent characteristics. Low‐generation dendrimers (generations 0–2) have increased branching and an amorphous structure, promoting diverse interactions due to their asymmetry.^[^ ^97^ ^]^ In contrast, high‐generation dendrimers (generations four and beyond, up to 12) possess a globular shape, enabling better encapsulation of hydrophobic drugs within their cavities and allowing for multiple drug molecules to bind to their surface (**Figure** **5**).^[^ ^98^ ^]^ Higher generations result in more voids within the structure, enhancing drug solubility. Additionally, high‐generation dendrimers often form a membrane‐like structure due to densely packed peripheries.^[^ ^99^ ^]^

Modifying the core, interior layers, and periphery enables precise control over dendrimer shape, internal cavities, and overall physicochemical properties.^[^ ^101^ ^]^ By selecting initiator cores (for example, phosphorus or nitrogen) and specific branching units, one can fine‐tune morphology and generation size.^[^ ^102^ ^]^ Terminal functional groups dictate solubility and reactivity: hydrophilic end groups confer solubility in polar solvents over a hydrophobic interior,^[^ ^103^ ^]^ while guest molecules engage within hydrophobic voids and bond via tertiary amines or amides through electrostatic and hydrogen bonding.^[^ ^104^ ^]^ Controlled release of entrapped therapeutic cargo is achieved by combining physical encapsulation with bonding modalities including hydrogen, covalent, and biodegradable linkages.^[^ ^105^ ^]^ Widely studied dendrimer classes in biomedicine and drug delivery include polypropylene imine dendrimers,^[^ ^106^ ^]^ polyamidoamine dendrimers,^[^ ^107^ ^]^ core‐shell tecto dendrimers,^[^ ^108^ ^]^ peptide dendrimers,^[^ ^109^ ^]^ and glycodendrimers.^[^ ^110^ ^]^

#### 2.2.3 Micelles

**Figure 6:**
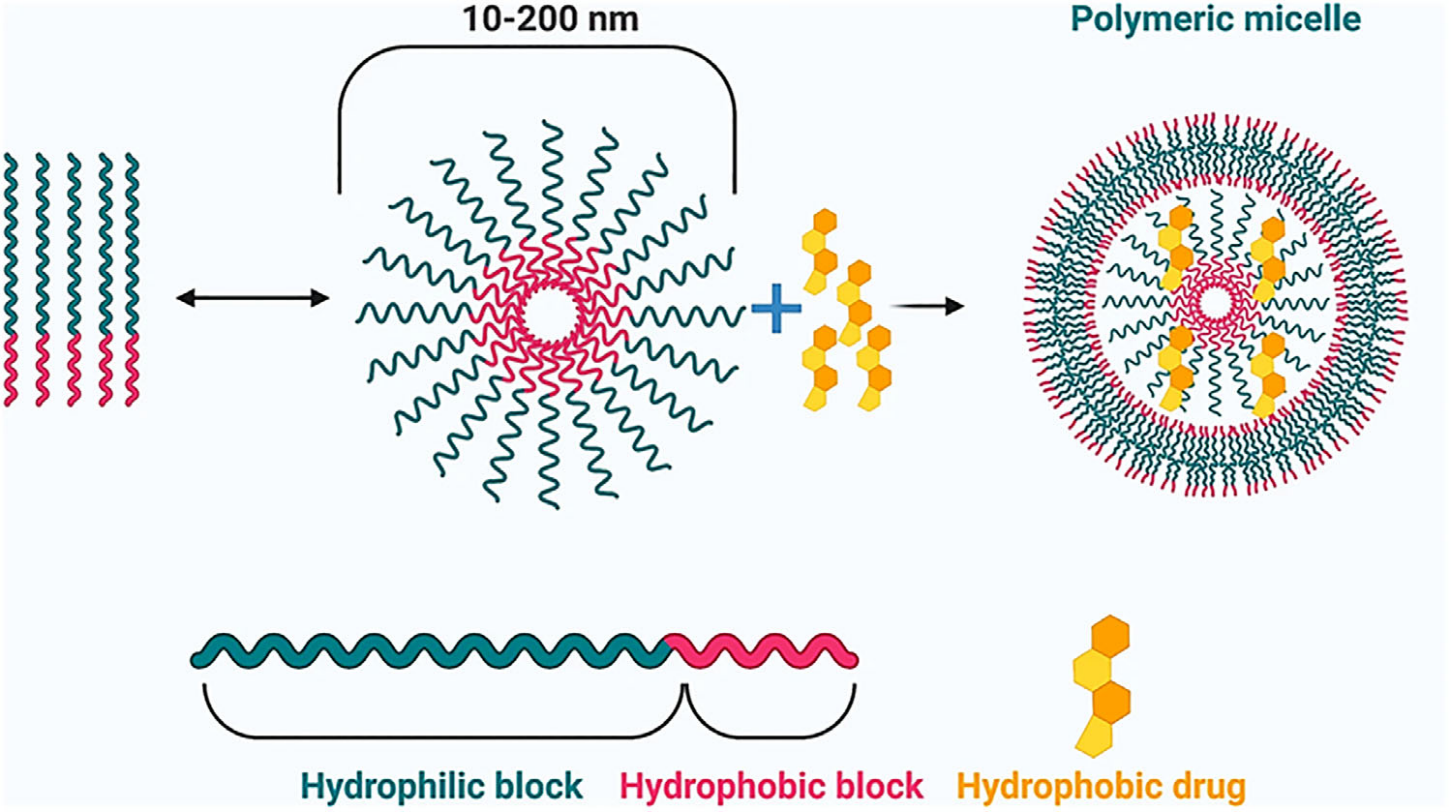
Schematic depiction of polymeric micelles. Self‐assembly of di‐block copolymers into a polymeric micelle takes place above the CMC. The hydrophobic drug is encapsulated into the hydrophobic core. Adapted with permission from ref. [113], Copyright Springer Nature 2022.

Polymeric micelles (PMs) exhibit a core‐shell structure due to the self‐assembly of amphiphilic block copolymers in water (**Figure** **6**). Initially acting as surfactants to lower surface tension, these molecules aggregate into micelles when the solution reaches the Critical Micellar Concentration (CMC).^[^ ^111^ ^]^ This concentration marks the threshold for micelle formation. Above the CMC, micelles remain stable due to both thermodynamic and kinetic factors, allowing a continuous exchange of unimers with the bulk phase. PMs, unlike low molecular weight surfactants, offer advantages such as lower CMC and improved kinetic stability. This stability is crucial for applications like drug delivery systems.^[^ ^112^ ^]^

PMs are highly versatile and influenced by polymer selection, solvent properties, and environmental conditions. Categorization is vital for tailoring PM characteristics. Types include diblock, triblock, multi‐block copolymers, graft polymers, and stimuli‐sensitive polymers, impacting self‐assembly and structure.^[^ ^114^ ^]^ Conventional micelles consist of amphiphilic polymers with hydrophilic shells and hydrophobic cores; reverse micelles invert this arrangement, and mixed micelles incorporate solubilizates within surfactant assemblies.^[^ ^115^ ^]^ Common materials include amphiphilic di‐block copolymers (for example, polystyrene‐poly(ethylene glycol)) and triblock copolymers (for example, poloxamers), graft polymers (for example, G‐chitosan), and ionic copolymers (for example, PEG‐poly(–caprolactone)‐g‐polyethyleneimine).^[^ ^116^ ^]^ Hydrophilic segments are typically PEG or alternatives such as poly(vinyl pyrrolidone), poly(acryloylmorpholine), or poly(trimethylene carbonate), while hydrophobic blocks include polypropylene oxide, poly(–caprolactone), and polymers or copolymers derived from glycolic and lactic acids.^[^ ^117^ ^]^

Preparation techniques shape micelle size, stability, and drug loading. Direct dissolution dissolves copolymers and drugs in aqueous media with stirring, sonication, or heating, triggering micelle formation upon dehydration of core‐forming blocks.^[^ ^118^ ^]^ Simple mixing of oppositely charged copolymers assembles micelles that encapsulate charged macromolecules such as nucleic acids and proteins within hydrophobic cores protected by PEG shells.^[^ ^119^, ^120^ ^]^ Solvent evaporation entails co‐dissolving the drug and polymer, evaporating the solvent to form a thin film, then hydrating to yield micelles with uniform size via sonication or high‐pressure extrusion.^[^ ^121^, ^122^ ^]^ Dialysis replaces organic solvents with water to induce micelle formation and remove solvents.^[^ ^123^ ^]^ Continuous processing offers precise control over parameters to achieve high drug loading and low polydispersity indices^[^ ^124^, ^125^ ^]^

PMs have been extensively investigated for intravenous chemotherapy delivery but also demonstrate promise for oral and topical administration (ocular, nasal, buccal).^[^ ^126^ ^]^ Their size (30–100 nm for permeable tumors, ≈30 nm for poorly permeable tumors) governs biodistribution and tissue penetration.^[^ ^127^, ^128^ ^]^ Surface characteristics such as neutral hydrophilic shells reduce protein corona formation and prolong circulation,^[^ ^129^ ^]^ while positive surface charge enhances mucoadhesion but may decrease colloidal stability.^[^ ^130^ ^]^ Micelle morphology extends beyond spherical shapes to rod‐, worm‐ or disk‐like structures, impacting circulation time, biodistribution, and cellular uptake.^[^ ^131^, ^132^ ^]^ Filomicelles exhibit elongated, flexible forms with slower clearance and enhanced tumor penetration when shorter segments fragment within tumors.^[^ ^133^ ^]^ Drug release proceeds via diffusion from intact micelles or micelle disassembly and requires both thermodynamic and kinetic stability achieved by modifying hydrophobic segments, crosslinking micelle cores, or forming polymer‐drug conjugates.^[^ ^134^ ^]^ Stability must be verified under biorelevant conditions, considering protein corona effects and potential micelle disaggregation on mucosal surfaces.^[^ ^135^ ^]^ Ongoing research focuses on stimuli‐responsive PMs, tailoring drug release in response to biological cues or artificial stimuli like pH, redox potential, enzymes, ultrasounds, magnetic fields, or temperature changes.^[^ ^136^ ^]^

#### 2.2.4 Polymer‐Drug Conjugates

**Figure 7:**
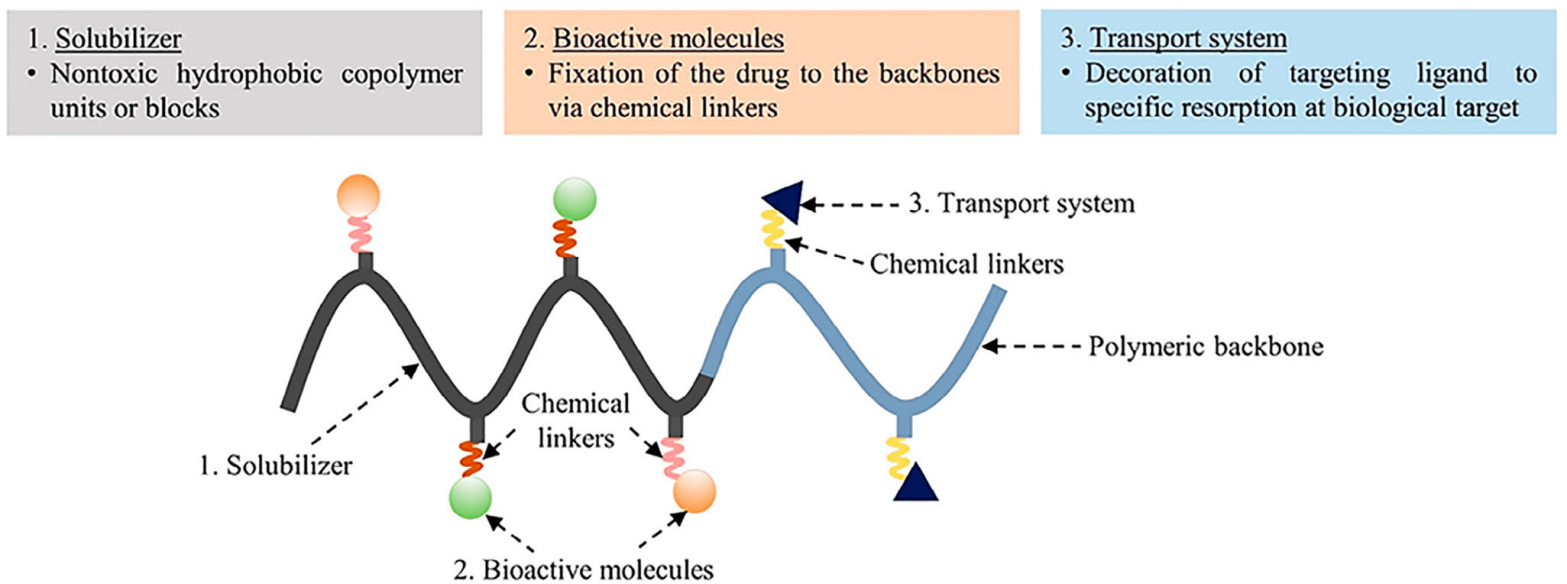
Basic structure of polymer‐drug conjugates. Adapted with permission from ref. [146], Copyright MDPI 2023.

Polymer‐drug conjugates (PDCs) are macromolecular constructs in which one or more therapeutic agents are covalently attached to polymer carriers to overcome limitations of conventional drug delivery.^[^ ^137^ ^]^ The first PDC was synthesized in 1955 by von Horst Jatzkewitz, who linked mescaline to poly(vinyl)pyrrolidone.^[^ ^138^ ^]^ In 1977, Abuchowski et al. demonstrated that PEGylation reduces protein immunogenicity, enhances solubility, and prolongs plasma half‐life.^[^ ^139^ ^]^ Subsequent advances by Kopecek and Duncan have produced multiple PDC formulations now marketed or in clinical trials.^[^ ^140^ ^]^ Besides therapeutic cargo, PDCs comprise an intricate assembly of components meticulously designed to optimize drug delivery and therapeutic outcomes (**Figure** **7**):

- Polymeric Backbone: It forms the structural foundation of the construct. Polymer selection is crucial in PDC design, offering diverse physicochemical properties. Polymers like PEG, N‐(2‐hydroxypropyl) methacrylamide copolymers, polyglycolic acid, polyvinyl alcohol, and polyvinylpyrrolidone enhance drug solubility, stability, and bioavailability. Their biocompatibility minimizes adverse effects upon administration.^[^ ^141^ ^]^
- Linker Molecules: Crucial for connecting components in PDCs, linkers are selected or crafted to respond to distinct stimuli, internal or external, facilitating controlled drug release. They often undergo cleavage triggered by environmental factors like enzymes, pH shifts, or glutathione, enabling precise drug release at the target site and reducing off‐target effects.^[^ ^142^ ^]^ Various stimuli‐sensitive linkers, such as protease‐sensitive peptides, matrix metalloprotease‐sensitive peptides, azo‐bonds, and redox‐sensitive disulfide linkers, are used to customize PDC release kinetics to specific disease conditions.^[^ ^143^ ^]^
- Targeting Ligands: They enable precise delivery of therapeutic agents to target cells or tissues by recognizing specific receptors or exploiting unique diseased tissue environments.^[^ ^144^ ^]^ Examples include D‐mannose and galactosylated ligands for mannose receptor targeting, glycyrrhetinic acid for hepatocyte recognition, and albumin, lysozyme, and receptor‐associated proteins for megalin and cubilin receptor targeting. Integrating these ligands improves delivery selectivity, reducing systemic toxicity and off‐target effects.^[^ ^145^ ^]^

The synthesis of PDCs involves a meticulous process to produce drug delivery systems with improved effectiveness and regulated drug release. It starts by selecting a compatible polymeric carrier and therapeutic drug, ensuring chemical functionality for conjugation.^[^ ^147^ ^]^ Coupling agents such as EDC, DCC, NHS, and HOBt activate functional groups on both components, forming stable covalent bonds. Site‐specific coupling can preserve the drug's biological activity, enhancing specificity in conjugation.^[^ ^148^, ^149^ ^]^ Recent advancements involve employing homo‐ and hetero‐bifunctional coupling reagents for precise control over conjugation strategies.^[^ ^150^ ^]^ Optimization of reactions, purification, and characterization processes ensures purity, structural integrity, drug loading, and stability of resulting PDCs.^[^ ^151^ ^]^

#### 2.2.5 Protein NPs

Protein nanoparticles, constructed primarily from natural sources like albumin, gelatin, or silk fibroin, or synthesized through genetic engineering, are ideal carriers at the nanoscale. They possess enzymatic degradability and induce minimal immune response. Their amphiphilic properties enable interaction with both hydrophilic and hydrophobic substances. Abundant hydroxyl, amino, and carboxyl groups facilitate chemical modification, allowing covalent or non‐covalent attachment of various ligands and drugs, thus offering significant surface modification possibilities.^[^ ^152^, ^153^ ^]^

Proteins from diverse sources, such as animals, plants, insects, and recombinant protein expression systems, have been explored for drug delivery. Some key examples are as follows:

- Albumin: It is a globular protein found in abundance in human and animal blood plasma. It has a relatively simple structure, consisting of a single polypeptide chain with 585 amino acid residues folded into three homologous domains.^[^ ^154^ ^]^ Sourced from various origins like egg white (ovalbumin), bovine serum albumin, and human serum albumin, it plays a crucial role in maintaining osmotic pressure and transporting nutrients to cells. Numerous drugs and molecules bind to albumin, utilizing it as a carrier and depot protein.^[^ ^155^ ^]^ Highly soluble in water and diluted salt solutions (up to 40% w/v at pH 7.4), albumin serves as an excellent macromolecular carrier for a diverse array of drugs.^[^ ^156^ ^]^ With stability within a pH range of 4 to 9 and tolerance to heat up to 60 °C for 10 h, it remains unaffected. Albumin nanocarriers, featuring biodegradability and ease of preparation, boast well‐defined sizes and surface reactive functional groups (thiol, amino, and carboxyl) useful for ligand binding and surface modifications. Natural drug release from albumin nanoparticles can be achieved via protease digestion.^[^ ^157^ ^]^
- Gelatin: Gelatin, a denatured protein derived from animal collagen, is extensively employed in pharmaceuticals, cosmetics, and food industries due to its unique properties. It functions as a Polyampholyte, possessing cationic, anionic, and hydrophobic groups in balanced proportions. A gelatin molecule typically carries 13% positive charge (from lysine and arginine), 12% negative charge (from glutamic and aspartic amino acids), and 11% hydrophobic amino acids (including leucine, isoleucine, methionine, and valine). Commercially, gelatin is available in cationic (from pig skin) and anionic (from bovine collagen) forms, serving as a proteinaceous material suitable for nanoparticle synthesis.^[^ ^158^ ^]^ It is FDA‐approved as a safe excipient for pharmaceutical formulations, offering biodegradability, non‐toxicity, and facile chemical modification or cross‐linking, thereby holding promise for drug delivery systems. Crosslinking agents like glutaraldehyde can enhance its stability and control drug release.^[^ ^159^ ^]^
- Elastin: It is a crucial protein in connective tissues (prevalent in arterial walls), conferring elasticity and shape‐restoration properties. Derived from tropoelastin, a 60–70‐kDa protein, it exists in open globular and distended polypeptide forms. Two elastin‐based polypeptides for drug delivery include –elastin, which aggregates at a specific temperature known as the cloud point (CP), and elastin‐like polypeptides (ELPs) with repetitive sequences.^[^ ^160^ ^]^ ELPs demonstrate temperature‐dependent self‐assembly below a modifiable transition temperature. Through genetic engineering, recombinant ELPs have been developed to mimic natural elastin without inducing an immune response.^[^ ^161^ ^]^ ELPs offer advantages such as tailored pharmacokinetics, precise molecular weight control, monodispersity, drug molecule conjugation, and targeted nanoparticle delivery. These polymers exhibit rapid phase transitions in response to temperature fluctuations.^[^ ^162^ ^]^
- Casein: Casein, the main protein in milk, is advantageous for formulating drug‐loaded nanoparticles due to its cost‐effectiveness, availability, and stability under heat and mechanical stress. It has favorable physicochemical properties, such as ion and molecule binding, self‐assembly, emulsification, and gel formation.^[^ ^163^ ^]^ It offers protection against radiation, particularly in the ultraviolet spectrum. With hydrophilic and hydrophobic amino acids, Casein forms self‐regulating micelles sized 50–500 nm, stabilized by casein kappa through electrostatic and spatial repulsion.^[^ ^164^ ^]^ These micelles, naturally occurring in milk, maintain stability during dairy product processing and are effective for transporting hydrophobic medicines, protecting them from degradation and oxidation. Notably, casein nanoparticles can undergo lyophilization without cryo‐protectants.^[^ ^165^ ^]^
- Silk proteins: Silk, derived from arthropods such as silkworms and spiders, comprises fibroin, a structural protein, and sericin, an adhesive protein. Sericin, constituting 20–30% of mulberry cocoon weight, enhances silk fiber strength and is utilized in nanoparticle preparation.^[^ ^166^ ^]^ Recent research highlights sericin's nonimmunogenic and biocompatible properties, finding applications in scaffolds, hydrogels, and nanoparticles.^[^ ^167^ ^]^ Silk sericin nanoparticles, combined with polymers like poloxamer or PEG, self‐assemble into drug carriers (100–400 nm). Fibroin, comprising 65–85% of silk proteins, offers exceptional physical properties, low immunogenicity, and biocompatibility.^[^ ^168^ ^]^ Silk fibroin nanoparticles, as drug carriers, exhibit controllable size, minimal cytotoxicity, stability, and hydrophobic drug loading capabilities.^[^ ^169^ ^]^ Explored for cancer therapy, they've been used in a carrier‐in‐carrier system for regenerative cell therapy and nanomedicine. Silk fibroin nanoparticles facilitate transdermal drug delivery and can be customized for site‐specific delivery through surface conjugation of targeting molecules. The self‐assembly ability, mechanical strength, and low inflammatory response of silk proteins make them promising for drug delivery.^[^ ^170^ ^]^
- Soy: Soybeans offer abundant plant protein, primarily in the form of soy protein isolate (SPI), prized for its nutritional value and versatility. SPI consists mainly of glycinin (MW = 360 000) and –conglycinin (MW = 180 000), with glycinin being dominant.^[^ ^171^ ^]^ These proteins form spherical structures in water, with hydrophilic outer layers and hydrophobic cores, along with small water‐soluble aggregates. Introducing dissolvents or cross‐linking agents further aggregates SPI molecules, producing various nanostructures.^[^ ^172^ ^]^ This structural manipulation allows for modifying drug release patterns. The well‐balanced amino acid composition in SPI accommodates different drugs.^[^ ^173^ ^]^ SPI nanoparticles can be produced by desolvation from fresh SPI or by utilizing glycinin from defatted soy flour via coacervation. These nanoparticles show potential in pharmaceutical applications by finely adjusting drug release patterns with added linker agents. Additionally, SPI finds extensive use in the food industry, leveraging its nutritional value and functional properties as an ingredient.^[^ ^174^, ^175^ ^]^
- Virus‐like particles (VLPs): Emerging from viral capsid engineering, VLPs are noninfectious protein‐based nanoparticles devoid of viral genomes. These structures, also known as viral protein cages, consist solely of viral coat proteins, making them ideal for secure therapeutic cargo transport.^[^ ^176^ ^]^ VLPs can encapsulate diverse medicinal compounds like proteins, peptides, siRNA, and chemotherapeutic drugs, along with imaging agents.^[^ ^177^ ^]^ Surface modifications, such as PEGylation, enhance targeting and reduce phagocytosis. Derived from animal viruses (e.g., hepatitis B), bacteriophages (e.g., MS2, Q‐, P22), and plant viruses (e.g., CCMV, CPMV), VLP manufacturing primarily employs recombinant technologies and chromatography for purification.^[^ ^178^ ^]^ Their uniform size enables precise drug loading, crucial for pharmacokinetic studies. Moreover, they hold promise in cancer treatment, penetrating tumor tissues for targeted drug delivery while evading liver macrophage clearance due to their small size.^[^ ^179^ ^]^

**Figure 8:**
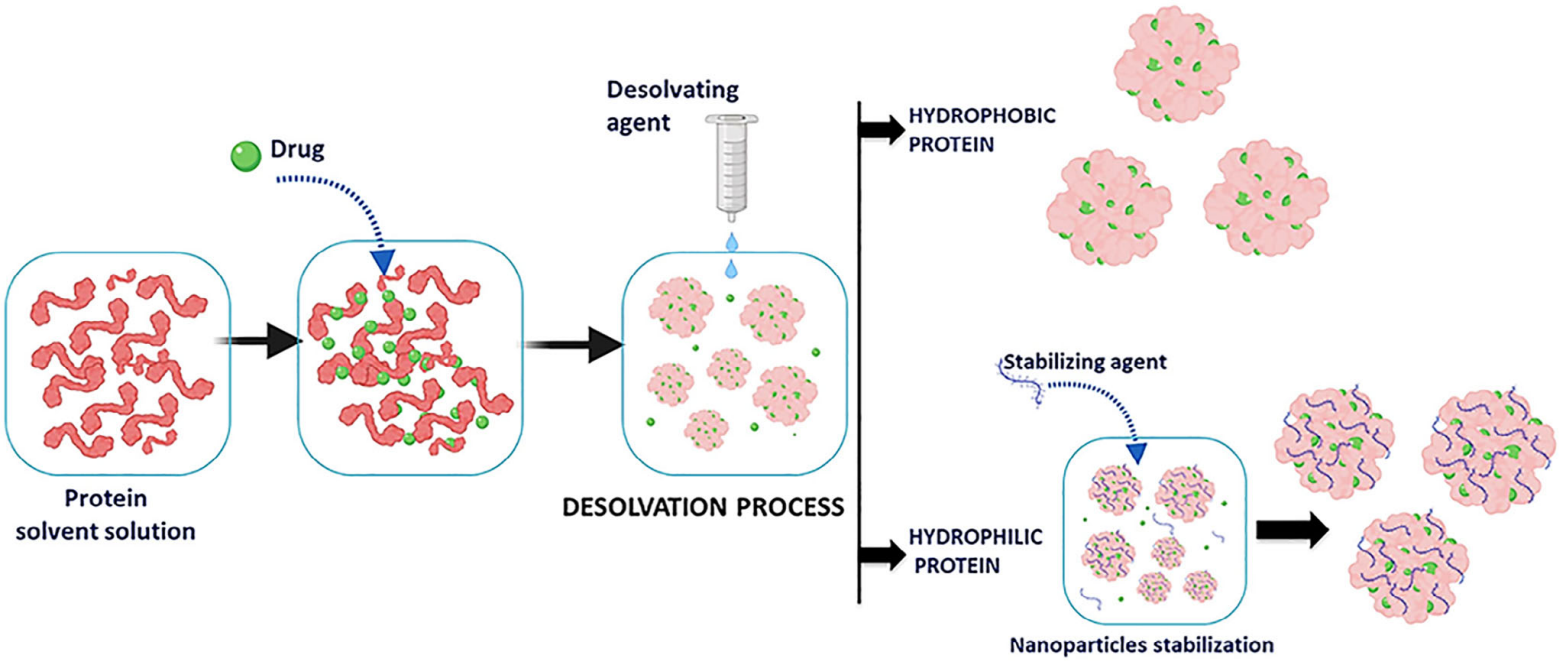
Diagram illustrating the preparation of Protein nanoparticles via Desolvation. Adapted with permission from ref. [184], Copyright Elsevier 2020.

Protein nanoparticle preparation methods vary depending on desired properties and applications. Coacervation/desolvation is common, altering solvent conditions to induce protein phase separation and nanoparticle formation (**Figure** **8**). Factors like solvent polarity, pH, ionic strength, and electrolytes affect solubility and nanoparticle size.^[^ ^180^ ^]^ Cross‐linking agents like glutaraldehyde stabilize these nanoparticles. Complex coacervation, ideal for gene therapy, uses electrostatic interactions between charged proteins and polyelectrolytes to entrap DNA or oligonucleotides in nanoparticles induced by pH adjustments and salts.^[^ ^181^, ^182^ ^]^ Electrospray applies high voltage to a protein solution, forming aerosolized liquid droplets containing protein nanoparticles collected afterward. It's useful for elastin‐like peptide nanoparticles, enabling efficient drug and nucleic acid incorporation.^[^ ^183^ ^]^ Method choice depends on a specific protein, particle size, and intended applications.

#### 2.2.6 Nanogels

**Figure 9:**
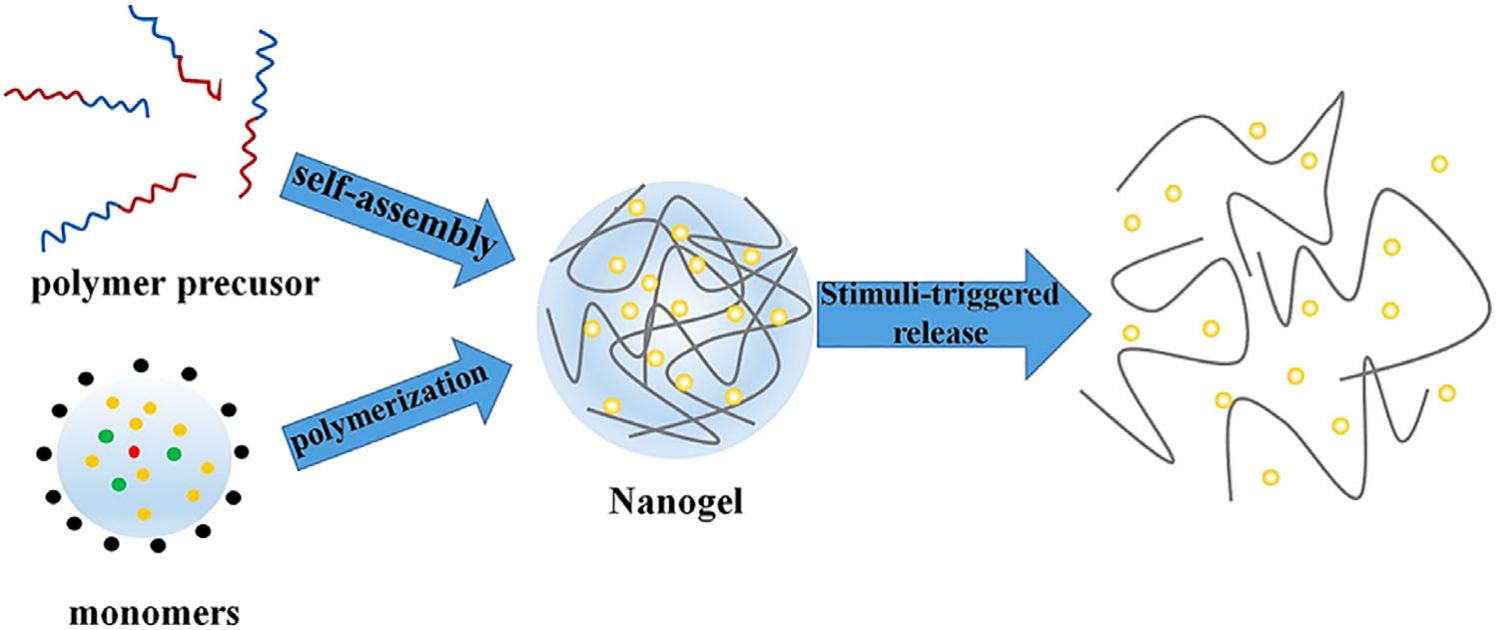
Schematic representation of nanogel preparation via self‐assembly or polymerization and its stimuli‐triggered drug release. Adapted with permission from ref. [187], Copyright Elsevier 2016.

Nanogels are intricate 3D networks of polymer chains linked through cross‐linking, combining hydrogel and nanoparticle properties due to their nanoscale size.^[^ ^185^ ^]^ Crucial to their synthesis are cross‐links, which can be physical or chemical. Nanogels can absorb significant amounts of water or biological fluids while maintaining their structure, thanks to hydrophilic functional groups (─OH, ─CONH‐, ─CONH2─, ─SO3H) within the polymer network. They swell rather than dissolve due to internal cross‐links, making them promising for various applications.^[^ ^186^ ^]^ Research highlights their potential as drug‐delivery carriers due to high drug‐loading capacity, stability, and responsiveness to environmental stimuli like ionic strength, pH, and temperature variations (**Figure** **9**).^[^ ^187^ ^]^ These qualities make nanogels ideal for advanced pharmaceutical applications. Principal approaches for the synthesis of nanogels are discussed below:

- Self‐assembly of polymer chains: This process, often carried out in mild aqueous conditions, involves controlled aggregation of amphiphilic or hydrophilic polymers. Interactions occur via electrostatic attractions, Van der Waals forces, hydrogen bonding, or hydrophobic interactions.^[^ ^188^ ^]^ Parameters like polymer concentration, amphiphilic nature, functional groups, pH, ionic strength, and temperature are adjusted to control nanogel size.^[^ ^189^ ^]^ Polysaccharide‐based nanogels, with hydrophobic modifications, exemplify this approach, where polymer hydrophobic segments drive nanoparticle formation.^[^ ^190^ ^]^ Although physically cross‐linked nanogels avoid toxic cross‐linkers, their stability in biological environments may limit biomedical applications.
- Polymerization of monomers: Nanogels can also be synthesized through polymerization of monomers in either a homogeneous phase or a micro‐/nano‐heterogeneous phase.^[^ ^191^ ^]^ This method is further categorized into emulsion and inverse emulsion polymerization. In inverse emulsion polymerization, stable nanogels are formed by adding specific co‐monomers that serve as bifunctional cross‐linkers.^[^ ^192^ ^]^ Polymerization can also occur within oil‐in‐water nano‐emulsions or aqueous suspensions.^[^ ^193^ ^]^ For example, poly(methacrylic acid‐grafted‐PEG) nanogels, promising for oral protein delivery, are synthesized using UV‐initiated free‐radical solution/precipitation polymerization.^[^ ^194^ ^]^ Monomers such as methacrylic acid and PEG, along with a cross‐linker, yield controlled nanogel formation.
- Cross‐linking of polymers: An alternative nanogel synthesis method involves the covalent cross‐linking of polymer chains to create robust networks. This method has proven effective in producing diverse functional nanogels tailored for drug delivery.^[^ ^195^ ^]^ For instance, cationic nanogels for polynucleotide transport are created by linking double‐activated PEG to branched PEI within an oil/water emulsion. The flexibility of this technique is demonstrated through disulfide‐based cross‐linking, which imparts responsiveness and biodegradability to nanogels.^[^ ^196^ ^]^ A specific instance involves polymers with PEG and pyridyl disulfide. Furthermore, diamines serve as advantageous cross‐linkers due to their reactivity with various functional groups.^[^ ^197^ ^]^
- Template‐assisted nanofabrication: The PRINT (Particle Replication in Non‐wettable Templates) method revolutionizes nanogel synthesis by enabling precise control over size, composition, shape, and surface functionality. Initially, a master template is crafted through lithographic techniques.^[^ ^198^ ^]^ Liquid fluoropolymer is then applied to the template's surface and cross‐linked via photochemical means, forming nanoscale cavities.^[^ ^199^ ^]^ Organic liquid precursors fill these cavities through capillary action, yielding nanogels. PRINT offers superior loading control of pharmaceuticals and biomacromolecules, enhancing its potential for drug delivery.^[^ ^200^ ^]^

Nanogels exhibit a range of architectures tailored for drug delivery and biomedical applications. Artificial chaperone nanogels, such as cross‐linked polyion‐anionic systems (for example, PEI‐PEG or PEG‐CL‐PEI), function as synthetic molecular chaperones and carriers for polynucleotides and hydrophobic drugs.^[^ ^201^ ^]^ Cholesterol‐bearing pullulan nanogels offer multiple hydrophobic zones for drug and protein entrapment.^[^ ^202^ ^]^ Core‐shell nanogels feature distinct compartments that respond to stimuli like temperature, pressure, or pH; they are typically prepared by precipitation, batch or seed polymerization, and may be coated with secondary nanoparticles to enhance specificity in temperature‐sensitive therapies.^[^ ^203^, ^204^ ^]^ Hairy nanogels possess stimuli‐responsive shell and can be synthesized via grafting, controlled radical polymerization or convenient one‐pot methods, with size adjustable by monomer concentration.^[^ ^205^, ^206^ ^]^ Hollow nanogels composed of temperature‐sensitive polymers provide large storage capacity and controlled release; common fabrication techniques include layer‐by‐layer assembly, lipid or block copolymer self‐assembly, template methods, and ultrasonic fabrication.^[^ ^207^, ^208^ ^]^ The introduction of mesoporous channels further increases drug loading, making these nanogels versatile platforms for responsive drug delivery and other biomedical uses.^[^ ^209^, ^210^ ^]^

### 2.3 Nanocrystals

Nanocrystals (NCs), or nanosuspensions, in pharmaceuticals are ultra‐small solid drug particles, typically sized from 10 to 1000 nanometers. They're coated or stabilized by surfactants or polymers, which often form during drug molecule crystallization.^[^ ^211^ ^]^ NCs boast high drug‐loading potential, typically ranging from 50% to 90% (w/w), although they can theoretically reach 100%. These colloidal dispersions offer improved solubility and drug loading due to their large surface area.^[^ ^212^ ^]^ Unlike other nanoparticulate systems, NCs primarily consist of an active pharmaceutical ingredient with minimal surfactant content. This unique composition allows for therapeutic concentrations at low doses, reducing toxic side effects associated with additional carrier materials.^[^ ^213^ ^]^

**Figure 10:**
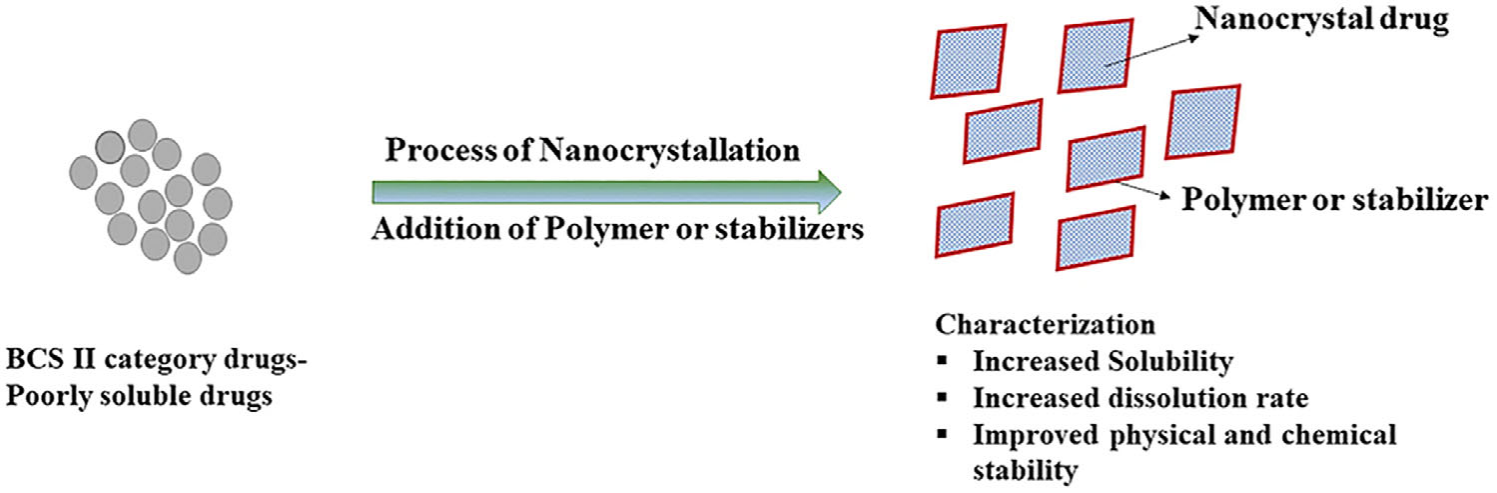
A diagram illustrating the nanocrystallization process used to enhance the physicochemical properties of drugs with low solubility. Adapted with permission from ref. [213], Copyright Springer Nature 2022.

NCs are versatile, being prepared in diverse media such as aqueous or non‐aqueous solutions, often with safe additives like surfactants or sugars for stability. These stabilizers are vital to maintaining colloidal stability and preventing NC aggregation. They are instrumental in enhancing the solubility, dissolution rate, and bioavailability of poorly water‐soluble drugs (BCS Class II) or potential new compounds (**Figure** **10**).^[^ ^214^ ^]^ NCs provide a flexible drug delivery platform suitable for various routes, like oral or intravenous administration. They can be processed into solid or sterile injectable forms to suit specific therapeutic needs.^[^ ^215^ ^]^ Notably, orally administered NCs swiftly disintegrate, aiding rapid drug absorption, which is especially advantageous for fast‐acting drugs. By modifying NC structure, sustained or targeted release of therapeutic agents can be achieved, enabling lower doses with reduced side effects. Intravenously administered NCs exhibit improved biodistribution due to slower dissolution in the bloodstream, enhancing local concentration, and reducing systemic effects.^[^ ^216^ ^]^

Preparation techniques for NCs can be divided into top‐down, bottom‐up, and combination approaches.

- Top‐down techniques: It rely on physicomechanical processes to reduce particle size. Wet ball milling uses shear and attrition between milling media (e.g., ceramic beads) and drug particles in a surfactant‐stabilized liquid, with efficiency governed by media selection, dispersion viscosity, temperature, and initial particle characteristics.^[^ ^217^, ^218^ ^]^ HPH, including microfluidization and piston‐gap homogenization, applies intense mechanical forces: microfluidization fractures particles via a high‐pressure jet, while piston‐gap homogenization forces suspensions through narrow gaps to induce cavitation and collisions.^[^ ^219^ ^]^ These scalable methods are effective for commercial nanocrystal production,^[^ ^220^ ^]^ but they require substantial energy and time, often involving multiple cycles or extended milling, and pose contamination risks from grinding media, particularly for intravenous formulations.^[^ ^221^ ^]^ Rigorous temperature control is also essential to mitigate heat generation. Despite these challenges, top‐down approaches markedly enhance drug solubility, dissolution rate, and bioavailability, driving progress in pharmaceutical formulations and delivery systems.^[^ ^222^ ^]^
- Bottom‐up techniques: These techniques employ physicochemical processes to precisely control nanocrystal properties. Cryogenic solvent evaporation atomizes a drug solution into liquid nitrogen, rapidly freezing droplets that are then lyophilized to remove solvents and yield nanocrystals with defined attributes.^[^ ^223^ ^]^ High‐gravity precipitation combines centrifugation with controlled precipitation, while evaporation precipitation in aqueous media at elevated temperatures followed by freeze‐drying further refines particle formation^[^ ^224^ ^]^ Supercritical fluid methods, including rapid expansion of supercritical solutions or using supercritical fluids as antisolvents, permit tight control over particle size and morphology .^225^ ^]^ Precise nucleation is achieved through mixing techniques ranging from magnetic stirring and sonication to advanced confined impinging jet reactors and multiple inlet vortex mixers, which create homogeneous, supersaturated solutions that produce uniformly small nanocrystals.^[^ ^226^ ^]^ Solvent removal methods such as spray drying, freeze‐drying, spray‐freezing into liquid and controlled crystallization during freeze‐drying eliminates solvents while preserving drug's crystalline structure.^[^ ^227^ ^]^
- Combination approaches: It integrates elements from both top‐down and bottom‐up methods to tailor NCs with specific attributes.^[^ ^228^ ^]^ Noteworthy methods include Nanoedge, H42, and H96. Nanoedge dissolves the drug in a suitable solvent, undergoes microprecipitation to form fragile drug particles, and utilizes HPH to further reduce particle size and enhance stability. This method enables precise control over particle size and distribution.^[^ ^229^ ^]^ H42 technology employs non‐aqueous spray drying, followed by HPH. Here, the drug dissolves in a non‐aqueous solvent, undergoes spray drying to yield a dry, fine powder, and is processed through HPH, enhancing dispersibility and being especially beneficial for hydrophobic drugs.^[^ ^230^ ^]^ H96, akin to Nanoedge, merges microprecipitation and HPH but emphasizes minimizing the time between precipitation and homogenization. Conducting precipitation directly within the homogenizer's dissipation zone yields very small drug particles, optimizing drug dissolution and bioavailability.^[^ ^231^ ^]^

Characterization of NCs is essential in drug development, with dynamic light scattering (DLS) measuring particle size and distribution, and zeta potential assessing surface charge.^[^ ^232^ ^]^ Imaging techniques such as SEM, TEM, and AFM complement DLS by revealing morphology, while X‐ray powder diffraction and differential scanning calorimetry determine crystallinity.^[^ ^233^ ^]^ Raman and FT‐IR spectroscopy monitors crystallization processes and drug‐excipient interactions, and stability is evaluated by DLS, absorbance measurements, and HPLC assays for chemical integrity.^[^ ^234^ ^]^ Assessing dissolution behavior and drug release rates is critical to establish in vitro‐in vivo correlations, taking into account the influence of particle size and stabilizer choice on release kinetics.^[^ ^235^ ^]^ NCs are thermodynamically prone to aggregation and Ostwald ripening due to their high interfacial free energy.^[^ ^236^ ^]^ Selecting appropriate stabilizers (ionic surfactants such as sodium dodecyl sulfate, non‐ionic surfactants such as Tween and TPGS, polymeric stabilizers such as HPMC, PVA and PVP, and novel agents like plant‐derived saponins) is vital to impart electrostatic repulsion and steric hindrance while enhancing solubility and dissolution rates.^[^ ^237^ ^]^ TPGS is particularly valued for its inhibition of P‐glycoprotein efflux, which helps overcome drug resistance in cancer therapy, and for conferring extended‐release kinetics and improved efficacy against chemotherapy‐resistant tumors.^[^ ^238^ ^]^

### 2.4 Inorganic Nanoparticles

#### 2.4.1 Metallic NPs

Metals are vital for cellular functions, acting as cofactors, catalysts, and structural elements. Transition metals like iron (Fe), copper (Cu), and zinc (Zn) are crucial in enzymatic reactions, facilitating essential biochemical processes. Iron is essential for oxygen transport and energy production, copper aids electron transfer, and zinc is vital for DNA replication and immune function.^[^ ^239^ ^]^ Noble metals such as gold (Au) and silver (Ag) have unique properties that are useful in nanomedicine.^[^ ^240^ ^]^ Historically known for their antimicrobial effects, they are now studied at the nanoscale for medical applications.^[^ ^241^ ^]^ Metallic nanoparticles, due to their biocompatibility, interact effectively within biological systems. Their size‐dependent properties enable precise targeting at cellular and molecular levels.^[^ ^242^ ^]^ Synthesizing metallic nanoparticles is crucial for tailoring properties to specific nanomedical uses. Various synthesis methods, including physical, chemical, and biological approaches, offer versatile tools for controlling nanoparticle size, shape, and surface features.

- Physical Approach: Techniques such as vapor condensation, laser ablation, and ball milling are employed in the physical approach to tailor the nanoscale characteristics of particles. Vapor condensation utilizes controlled environments to condense metal vapor into nanoparticles, enabling the production of ultra‐small particles with precise size control and narrow size distributions.^[^ ^243^ ^]^ Laser ablation harnesses laser energy to form nanoparticles rapidly and efficiently, potentially yielding unique morphologies.^[^ ^244^ ^]^ Meanwhile, ball milling breaks down bulk materials into nanoparticles using mechanical energy, providing a versatile, large‐scale production method.^[^ ^245^ ^]^ Physical approaches are invaluable for applications requiring precise control over nanoparticle size and morphology, particularly in advanced medical applications where uniformity and precision are essential.^[^ ^246^ ^]^
- Chemical Approach: Metal ion reduction to nanoparticles via diverse chemical agents is a common methodology. The Turkevich method, using citrate as a reducing agent, is widely employed for gold salt reduction, yielding well‐defined gold nanoparticles (AuNPs) vital for biomedical applications.^[^ ^247^ ^]^ Seed‐mediated growth, another chemical method, employs pre‐formed seed nanoparticles to regulate larger nanoparticle growth, offering flexibility in property manipulation. The choice of reducing agent is critical; sodium borohydride is frequently used due to its efficacy in producing stable nanoparticles.^[^ ^248^ ^]^ Polyol‐based agents like ethylene glycol provide a scalable synthesis route for various metallic nanoparticles.^[^ ^249^ ^]^ Chemical approaches extend to silver, copper, and other metallic nanoparticles, each serving specific nanomedical applications. These methods offer reproducibility, scalability, and tunability, with researchers adjusting parameters such as reaction temperature and concentration and reducing agent selection to refine nanoparticle properties.^[^ ^250^ ^]^
- Biological Approach: It represents a cutting‐edge and environmentally sustainable approach, often termed as green synthesis. This method harnesses the inherent reducing capabilities of living organisms, such as bacteria, fungi, or plant extracts, to facilitate the conversion of metal ions into nanoparticles.^[^ ^251^ ^]^ The biological approach is characterized by its eco‐friendly nature, simplicity, and potential for large‐scale production. In the context of nanomedicine, the biological approach offers distinct advantages, particularly in enhancing the biocompatibility and reducing the toxicity of metallic nanoparticles.^[^ ^252^ ^]^ Plant‐mediated synthesis, for instance, has gained attention due to the diverse range of phytochemicals present in plant extracts, acting as both reducing and stabilizing agents.^[^ ^253^ ^]^ This green synthesis route not only minimizes the use of hazardous chemicals but also introduces natural compounds that may impart additional therapeutic properties to the resulting nanoparticles.^[^ ^254^ ^]^

Some prominent metallic nanoparticles are discussed below:

- AuNPs: With a size range of 1–100 nanometers, these nanoparticles exhibit a surface plasmon resonance effect, making them highly efficient in absorbing and scattering light.^[^ ^255^ ^]^ This property is exploited in various diagnostic imaging techniques, such as surface‐enhanced Raman spectroscopy and photoacoustic imaging. The Turkevich method is a common synthesis approach. Additional methods, such as seed‐mediated growth and chemical reduction with alternative agents, provide control over size and shape, influencing their biomedical applications.^[^ ^256^, ^257^ ^]^
- Silver Nanoparticles (AgNPs): AgNPs possess remarkable antimicrobial properties, making them valuable in nanomedicine for wound healing, infection control, and medical device coatings.^[^ ^258^ ^]^ Ranging from 1–100 nanometers, AgNPs induce the generation of reactive oxygen species and disrupt microbial cell membranes. Chemical reduction methods, utilizing agents like sodium borohydride or citrate, are frequently employed for synthesis.^[^ ^259^ ^]^
- Iron Oxide Nanoparticles (FeO NPs): FeO NPs have extensive applications in magnetic resonance imaging (MRI), drug delivery, and hyperthermia treatment.^[^ ^260^ ^]^ These nanoparticles, typically superparamagnetic, can be manipulated under external magnetic fields, facilitating targeted drug delivery and imaging.^[^ ^261^ ^]^ Synthesis methods, including co‐precipitation, thermal decomposition, and microemulsion, offer control over size, composition, and surface characteristics, influencing their performance in biomedical applications.^[^ ^262^ ^]^
- Copper Nanoparticles (CuNPs): CuNPs exhibit promise in antitumor and antimicrobial therapies within nanomedicine. Their cytotoxic effects on cancer cells and antimicrobial properties against various microorganisms make them attractive candidates.^[^ ^263^ ^]^ Chemical reduction methods employing copper salts and reducing agents like sodium borohydride or hydrazine are commonly utilized.^[^ ^264^ ^]^

#### 2.4.2 CNTs

Carbon nanotubes (CNTs) exhibit a unique amalgamation of mechanical, electrical, and optical attributes, along with the potential for encapsulating various molecular compounds. Since their inception in 1991 by Iijima and collaborators, CNTs have emerged as promising nanomaterials in the realm of biomedical applications.^[^ ^265^ ^]^ Referred to colloquially as buckytubes, CNTs represent nanoscale hollow cylindrical structures formed from rolled‐up sheets of single‐layer carbon atoms. Each carbon atom in the structure is connected to three adjacent atoms, akin to graphite, resulting in a distinctive hexagonal structure and ‐ electrons conjugation through sp2 bonding.^[^ ^266^ ^]^ They can manifest as open‐ended structures or capped, with the latter exhibiting half of a fullerene molecule. Characterized by a high aspect ratio, CNTs typically feature diameters in the range of a few nanometers and lengths extending to several micrometers.^[^ ^267^ ^]^ The carbon backbone of CNTs is amenable to additional functionalization through various non‐covalent and covalent modifications.^[^ ^268^ ^]^ Classification based on structure delineates CNTs into single‐walled carbon nanotubes (SWCNTs) and multi‐walled carbon nanotubes (MWCNTs). SWCNTs consist of a single graphene cylinder, while MWCNTs incorporate at least two coaxial cylinders that enclose a hollow core.^[^ ^269^ ^]^

The synthesis of CNTs employs three primary methods: arc discharge, laser ablation, and chemical vapor deposition. All methods utilize a carbon source and energy to fabricate CNTs. In the arc discharge method, carbon electrodes undergo expulsion through an electric discharge (50–100 A, 20 V) in the presence of inert gas and low pressure, generating exceptionally high temperatures.^[^ ^270^ ^]^ This process vaporizes the surface of carbon electrodes, resulting in a rod‐shaped deposit of nanotubes. Pure carbon electrodes yield MWCNTs, whereas metal‐doped electrodes produce SWCNTs.^[^ ^271^ ^]^ The laser ablation approach mirrors this concept, utilizing a high‐intensity laser pulsed repeatedly as the energy source.^[^ ^272^ ^]^ Chemical vapor deposition emerges as a widely adopted method for the large‐scale production of CNTs. In this method, hydrocarbons such as CH4, acetylene, or carbon monoxide undergo decomposition at elevated temperatures using specific catalysts, yielding CNTs with high efficiency.^[^ ^273^, ^274^ ^]^ Post‐synthesis, impurities can be eliminated through acid treatment, magnetic purification, or size‐exclusion chromatography, enhancing the purity and applicability of the synthesized CNTs.^[^ ^275^ ^]^

**Figure 11:**
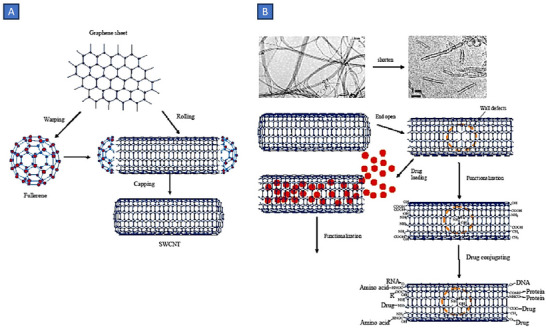
Development and modification of SWCNT for drug delivery applications. A) Diagram showing how SWCNTs are formed with both ends sealed. B) Diagram outlining the process for creating drug delivery systems using carbon nanotubes. Adapted with permission from ref. [280], Copyright Springer Nature 2011.

The hollow structure of CNTs provides an ideal nanocarrier platform, enabling the loading of bioactive compounds within their inner cavities or on their external surfaces.^[^ ^276^ ^]^ This unique feature not only enhances the stability and solubility of encapsulated biomolecules but also facilitates controlled release kinetics, minimizing side effects and optimizing therapeutic efficacy.^[^ ^277^ ^]^ Moreover, the tunable surface chemistry of CNTs allows for precise functionalization, enabling the attachment of targeting ligands or imaging agents. This functionalization enhances the specificity of CNTs, enabling them to selectively bind to specific cells or tissues, thereby improving the precision and efficiency of drug delivery (**Figure** **11**).^[^ ^278^ ^]^ Additionally, the high aspect ratio and nanoscale dimensions of CNTs enable them to penetrate cellular membranes, facilitating the intracellular delivery of therapeutic payloads.^[^ ^279^ ^]^

#### 2.4.3 Mesoporous Silica NPs

Mesoporous silica nanoparticles (MSNs) represent nanostructures based on silica, characterized by a solid framework exhibiting a well‐defined atomic‐level arrangement of mesopores, resulting in a substantial surface area exceeding 1000 m^2^g^−1^.^[^ ^280^ ^]^ Although the initial documentation of MSNs dates back to the early 1990s, the past decade has witnessed a surge in extensive research delving into their biomedical potential. This surge can be attributed to the unique attributes of MSNs, including superior biocompatibility, high chemical/biological stability, biodegradability, and the ease of tuning pore size within the 2 to 50 nm range.^[^ ^281^ ^]^ Four predominant methods for synthesizing MSNs have been identified, namely template‐directed technique, sol–gel approach, microwave‐assisted technique, and chemical etching.^[^ ^282^ ^]^ Key variables influencing the utilization of MSNs are particle size and pore volume, both of which can be manipulated by adjusting the silica source and operating parameters such as pH, temperature, and surfactant concentration within the reaction mixture.^[^ ^283^ ^]^

MSNs have proven effective carriers for a diverse array of cargo, encompassing drugs and macromolecules such as proteins, DNA, and RNA.^[^ ^284^ ^]^ The tunable pore size plays a pivotal role in tailoring MSNs for specific applications and desired release profiles. Larger pore diameters, up to 50 nm, are preferred for facilitating the delivery of macromolecules, whereas a smaller pore size is conducive to achieving a controlled release profile for drug molecules.^[^ ^285^ ^]^ Moreover, various pore morphologies, including hexagonal, cubic, concentric, and radial, have been reported, presenting an additional avenue for modulating release kinetics.^[^ ^286^ ^]^ Due to the inherent silicon dioxide matrix composition, MSNs are prone to hydrolytic breakdown through OH‐mediated nucleophilic attack in physiological fluids, ultimately forming ortho‐silicic acid that is subsequently eliminated through urine.^[^ ^287^ ^]^ In this context, surface modification is generally unnecessary for enhancing cytocompatibility. Functionalization strategies are primarily directed toward imparting targeted nanoparticle delivery or triggering drug release in response to external stimuli.^[^ ^288^ ^]^

### 2.5 Comparative Analysis of Nanoparticle Classes

As discussed, the field of nanomedicine encompasses a diverse array of nanocarrier platforms, each engineered to address specific therapeutic challenges. These systems vary widely in their physicochemical properties, including particle size, surface charge, composition, drug loading efficiency, and release kinetics; all of which significantly influence their biological performance, pharmacokinetics, and clinical outcomes. Importantly, their behavior in vivo is shaped not only by these inherent attributes but also by their interaction with biological systems, such as protein corona formation, immune recognition, and clearance mechanisms. While previous sections have examined the structural, functional, and clinical aspects of each nanoparticle class in detail, bringing these insights together in a comparative format helps clarify their relative advantages, limitations, and potential for clinical translation. Presenting this information side by side allows researchers and clinicians to more clearly evaluate how different formulation strategies influence key development considerations, including scalability in manufacturing, safety and toxicity profiles, regulatory requirements, and the feasibility of targeted delivery. **Table** **1** presents a side‐by‐side comparison of lipid‐based, polymeric, inorganic, and nanocrystal‐based platforms, focusing on key parameters that directly influence their clinical translation and commercial viability.

**Table 1:** Comparative Overview of Nanoparticle Classes.

Feature	Lipid‐based	Polymeric	Inorganic	Nanocrystals
Drug loading strategy	Encapsulation in a lipid bilayer or an aqueous core	Encapsulation or conjugation within a polymer matrix	Entrapment, adsorption, or surface conjugation	Nanoparticles consist of 100% active pharmaceutical ingredient (API)
Pharmacokinetics	Long circulation with PEGylation, hepatic clearance, EPR‐dependent delivery	Sustained release, customizable clearance via polymer chemistry	Prone to rapid RES uptake without stealth coatings	Rapid dissolution enhances absorption without complex distribution
Targeting capability	Modifiable with PEG, ligands, or aptamers	Easily functionalized with antibodies, peptides, or polymers	Limited targeting, often passive or via external fields	Lacks intrinsic targeting, relies on systemic distribution
Formulation complexity	Moderate, requires lipid optimization and stabilizers	High sensitive to polymer composition and processing parameters	Very high synthesis control is challenging	Low, requires surfactants or stabilizers to prevent aggregation
Stability	Moderate, may require cold chain for storage	Generally high with appropriate formulation	Physically and chemically stable, non‐biodegradable	Chemically stable, prone to physical aggregation if not stabilized
Toxicity	Low lipids and PEG are biocompatible	Low to moderate, depending on polymer type and degradation products	Potential for long‐term accumulation and metal‐related toxicity	Typically low, excipient toxicity can vary
Manufacturability	Scalable using microfluidics or ethanol injection methods	Scalable with nanoprecipitation and solvent evaporation methods	Batch‐dependent, hard to scale uniformly	Highly scalable with wet milling and HPH
Regulatory acceptance	High, with established pathways and precedents	Moderate, varies with polymer and delivery system	Low, requires full toxicology and biodegradation profiling	High, with several FDA and EMA‐approved products
Best application areas	Gene delivery, mRNA vaccines, antifungals, oncology	Chemotherapy, RNA delivery, and immune modulation	Imaging, diagnostics, and cancer hyperthermia	Oral delivery of poorly soluble BCS Class II and IV drugs

## 3 Overview of Market Size and Approved Products

Recent findings from the International Market Analysis Research and Consulting (IMARC) Group indicate that the global nanomedicine market achieved a significant valuation of US$ 263.9 Billion in 2023. Projections suggest a substantial escalation, with an anticipated valuation of US$ 719.5 Billion by 2032, reflecting a compound annual growth rate approximating 11.4% during the forecast period spanning from 2024 to 2032.^[^ ^289^ ^]^

Over the last few decades, the US FDA has sanctioned over 90 nanomedicine formulations for commercialization. This upward trend in approvals is anticipated to continue, fueled by both the escalating adoption of extant nano‐pharmaceuticals and the forthcoming approval of those undergoing clinical evaluation. The global landscape features over 100 companies/firms engaged in developing nanotechnology‐driven therapeutic solutions, with approximately one‐third comprising small to medium‐sized enterprises or start‐up ventures.^[^ ^290^ ^]^ Within the pharmaceutical domain, nanomedicine has exhibited notable predominance in oncology, commanding a significant 35% market share presently. This underscores the focal role of nanotechnology‐enabled research in cancer management, facilitated by enhanced therapeutic efficacy, precision targeting, and augmented safety profiles afforded by novel nano‐drug delivery platforms.^[^ ^291^ ^]^ Beyond oncology, nanomedicines have found utility across diverse clinical domains, encompassing neurological disorders, cardiovascular ailments, infectious maladies, diagnostic modalities, and medical imaging techniques, among others.

The growing interest of pharmaceutical companies in nanomedicine reflects a strategic response to the evolving landscape of healthcare needs. The increasing prevalence of complex diseases, such as neurological disorders and cancer, necessitates innovative therapeutic approaches that can address challenges such as drug resistance and limited bioavailability.^[^ ^292^, ^293^ ^]^ In tandem with heightened interest, substantial investments are being directed toward developing next‐generation drug products harnessing nanotechnology. These investments span various stages of the drug development pipeline, from early‐stage research and preclinical studies to clinical trials and commercialization efforts.^[^ ^294^ ^]^ The infusion of capital into nanomedicine research and development not only accelerates the translation of promising concepts into viable medical interventions but also fosters collaboration between academia, industry, and regulatory agencies to ensure the safety and efficacy of novel nanomedicine products.^[^ ^295^ ^]^

In addition to novel drug development, the emergence and development of generics of existing approved nanomedicine products are poised to contribute significantly to market growth. The availability of generic versions can enhance competition, drive down prices, and improve accessibility to essential nanomedicine treatments, particularly in resource‐constrained healthcare settings.^[^ ^296^ ^]^ Moreover, the evolution of nanomedicine beyond therapeutic applications into diagnostic and imaging modalities further broadens its potential impact across the continuum of healthcare delivery.

**Table** **2** presents a comprehensive synopsis of approved nanomedicine currently available in the market. This tabular exposition is organized according to the categorization of nanomedicine, delineating pertinent details such as formulation type, active pharmaceutical ingredient, and clinical indications.

**Table 2:** Approved and Marketed Nanomedicines for Different Diseases.

Sr. No.	Trade Name	Company	Formulation Type	Active Ingredient(s)	Approval Year	Indications/Clinical Applications	Refs.
**Lipid‐based Nanoparticles**							
1	Abelcet	Leadiant Biosciences	Liposome	Amphotericin B	1995	Treatment of invasive fungal infection in patients who are refractory to conventional amphotericin B therapy	[297]
2	Advate	Baxter	Liposome	Octocog alfa	2003	Replaces clotting factor VIII (antihemophilic factor) in patients with hemophilia A	[298]
3	AmBiosome	Gilead Sciences Inc.	Liposome	Amphotericin B	1997	Treatment of serious fungal infections such as systemic fungal infections, visceral leishmaniasis, and cryptococcal meningitis	[299]
4	Amphotec	Ben Venue Laboratories	Lyophilized lipid‐based powder	Amphotericin B	1996	Treatment of severe fungal infections, particularly in cases where renal impairment precludes the use of other treatments	[300]
5	Arikayce	PARI Pharma GmbH	Liposome	Amikacin	2018	For treating non‐tuberculous mycobacterial lung disease, specifically those caused by *Mycobacterium avium* complex	[301]
6	Comirnaty	Pfizer‐BioNTech	Lipid nanoparticles	Spike protein‐encoding mRNA	2021	Indicated for active immunization to prevent COVID‐19 caused by SARS‐CoV‐2 virus	[302]
7	Curosurf	Chiesi USA Inc.	Lipid nanoparticles	Poractant Alfa	1999	Indicated for the rescue treatment of respiratory distress syndrome in premature infants.	[303]
8	DaunoXome	Gilead Sciences Inc.	Liposome	Daunorubicin	1996	Treatment of AIDS‐related Kaposi's Sarcoma in patients with low CD4 cell counts (<200 cells/mm^3^)	[304]
9	Definity	Lantheus Medical Imaging	Lipid‐coated microbubbles	Perflutren	2001	Primarily employed as an ultrasound contrast agent to enhance the quality of echocardiograms	[305]
10	Depocyt	Pacira BioSciences	Liposome	Cytarabine	1999	Intrathecal treatment of lymphomatous meningitis	[306]
11	DepoDur	Endo Pharmaceuticals	Liposome	Morphine sulfate	2004	Treatment of pain following major surgical procedures, such as cesarean section, orthopedic surgery, and abdominal surgery	[307]
12	Diprivan	Fresenius Kabi	Lipid emulsion	Propofol	1989	It is utilized for the initiation and maintenance of sedation during monitored anesthesia care	[308]
13	Doxil/Caelyx	Sequus Pharmaceuticals	Liposome	Doxorubicin	1995/1996	Treatment of AIDS‐related Kaposi's sarcoma in patients after failure of prior systemic chemotherapy	[309]
14	Exparel	Pacira BioSciences	Liposome	Bupivacaine	2011	Indicated for producing postsurgical local analgesia via infiltration in patients aged six years and older	[310]
15	Inflexal V	Crucell/Berna Biotech Ltd	Liposome	Inactivated viral hemagglutinin	1997	For the active immunization against influenza virus infection in adults and children aged six months and older	[311]
16	Lipodox	Sun Pharma	Liposome	Doxorubicin	2013	It is an approved generic version of Doxil/Caelyx	[312]
17	Lipusu	Luye Pharma	Liposome	Paclitaxel	2003	Treatment of advanced HER2‐negative breast cancer	[313]
18	Marqibo	Acrotech Biopharma Inc.	Liposome	Vincristine	2012	Treatment of adult patients with Philadelphia chromosome‐negative acute lymphoblastic leukemia	[314]
19	Mepact	Takeda Pharmaceuticals	Liposome	Mifamurtide	2009	Treatment of high‐grade non‐metastatic osteosarcoma in patients aged between two and 30 years	[315]
20	Spikevax	Moderna Inc.	Liposome	Spike protein‐encoding mRNA	2020	Indicated for active immunization to prevent COVID‐19 caused by SARS‐CoV‐2 virus	[316]
21	Mosquirix	Glaxo Smith Kline	Lipid nanoparticles	Recombinant protein	2015	For immunization of children (6 weeks ‐ 17 months) against malaria caused by *Plasmodium falciparum*	[317]
22	Myocet	Elan Pharmaceuticals	Liposome	Doxorubicin	2000	First‐line treatment of metastatic breast cancer in combination with cyclophosphamide.	[318]
23	Onivyde	Ipsen Bio‐pharmaceuticals	Liposome	Irinotecan	2015	Treatment of adult patients with metastatic pancreatic (in combination with fluorouracil and leucovorin)	[319]
24	Onpattro	Alnylam Pharmaceuticals	Lipid nanoparticles	siRNA	2018	Treatment of nerve damage caused by hereditary transthyretin amyloidosis	[320]
25	Shingrix	Glaxo Smith Kline	Lipid nanoparticles	Recombinant protein	2017	Indicated for prevention of herpes zoster and post‐herpetic neuralgia in adults 50 years of age or older	[321]
26	Visudyne	Novartis AG	Liposome	Verteporfin	2000	Treatment of patients with predominantly classic subfoveal choroidal neovascularization	[322]
27	Vyxeos	Celator Pharmaceuticals	Liposome	Cytarabine and Daunorubicin	2017	Treatment of adults with acute myeloid leukemia	[323]
**Polymeric Nanoparticles**							
28	Abraxane	Abraxis BioScience	Protein nanoparticles	Paclitaxel	2005	Treatment of advanced breast cancer, non‐small cell lung cancer, and pancreatic cancer	[324]
29	Adynovate	Baxalta	Polymeric conjugate	Recombinant anti‐hemophilic factor VIII	2015	Treatment and control of bleeding in children/adults with congenital Factor VIII deficiency	[325]
30	Apealea	Adienne Pharma & Biotech	Polymeric micelle	Paclitaxel	2022	Treatment of ovarian cancer, specifically in adult patients with first relapse of platinum‐sensitive epithelial ovarian cancer, primary peritoneal cancer, and fallopian tube cancer.	[326]
31	Cimzia	UCB (Union Chimique Belge)	PEGylated antibodies	Certolizumab pegol	2008	Used to reduce the signs/symptoms of moderately to severely active Crohn's disease in adults	[327]
32	Copaxone	Teva Pharmaceuticals	Copolymeric nanosphere	Glatiramer acetate	1996	Treatment of relapsing forms of multiple sclerosis, including clinically isolated syndrome and relapsing‐remitting disease	[328]
33	Eligard	Tolmar Pharmaceuticals	Polymeric nanogel	Leuprolide acetate	2002	It is a sustained‐release depot indicated for the palliative treatment of advanced prostate cancer	[329]
34	Estrasorb	Novavax Inc.	Polymeric micelle	Estradiol	2003	Treatment of vulvar and vaginal atrophy in menopause	[330]
35	Genexol‐PM	Samyang Bio‐pharmaceuticals	Polymeric micelle	Paclitaxel	2007	Treatment of advanced breast cancer, non‐small cell lung cancer, and pancreatic cancer	[331]
36	Kadcyla	Genentech Inc.	Polymeric conjugate	Trastuzumab emtansine	2013	Treatment of early/metastatic HER2‐positive breast cancer	[332]
37	Krystexxa	Horizon Therapeutics	Polymeric conjugate	Pegloticase	2010	Treatment of chronic gout in adult patient refractory to conventional therapy	[333]
38	Macugen	OSI Pharmaceuticals	PEGylated antibodies	Pegaptanib sodium	2000	Treatment of neovascular age‐related macular degeneration	[334]
39	Mircera	Hoffman‐LaRoche	Polymeric conjugate	Methoxy PEG‐epoetin beta	2007	Treatment of anemia associated with chronic kidney disease in adult patients	[335]
40	Nanoxel M	Samyang Bio‐pharmaceuticals	Polymeric micelle	Docetaxel	2012	Treatment of breast cancer, non‐small cell lung cancer, ovarian cancer, and some AIDS‐related cancers	[336]
41	Neulasta	Amgen Inc.	Polymeric conjugate	Pegfilgrastim	2002	Indicated to decrease the incidence of infection manifested by febrile neutropenia	[337]
42	Oncaspar	Enzon Pharmaceuticals/ Baxter BioScienc	Polymeric conjugate	L‐asparaginase	1994	Treatment of acute lymphoblastic leukemia in pediatric patients and adults	[338]
43	Ontak	Eisai	Protein nanoparticles	Denileukin diftitox	1999	Treatment of cutaneous T‐cell lymphoma in patients who have received at least one prior systemic therapy	[339]
44	Paclical	Oasmia Pharmaceutical AB	Polymeric micelle	Paclitaxel	2015	Treatment option of a higher Paclitaxel dose with a shorter infusion time without mandatory premedication	[340]
45	Pegasys	Genentech Inc.	Polymeric conjugate	Interferon alfa‐2a	2002	Treatment of chronic hepatitis B in both HBeAg‐positive and HBeAg‐negative patients	[341]
46	PegIntron	Merck	Polymeric conjugate	Interferon alfa‐2b	2001	Treatment of chronic Hepatitis C in patients with compensated liver disease	[342]
47	Plegridy	Biogen	Polymeric conjugate	Peginterferon beta‐1A	2014	Treatment of relapsing forms of multiple sclerosis in adults, including clinically isolated syndrome and relapsing‐remitting disease	[343]
48	Rebinyn	Novo Nordisk Inc.	Polymeric conjugate	Coagulation Factor IX (Recombinant)	2017	Used to replace clotting Factor IX that is missing in patients with hemophilia B	[344]
49	Restasis	Allergan	Polymeric nano emulsion	Cyclosporin	2002	Treatment of severe keratitis in dry eye patient	[345]
50	Somavert	Pharmacia	Polymeric conjugate	Pegvisomant	2003	Treatment of acromegaly in patients who have had an inadequate response to surgery or radiation therapy	[346]
51	Taxotere	Sanofi Aventis	Polymeric micelle	Docetaxel	1996	Treatment of various types of locally advanced or metastatic cancers	[347]
52	Verelan PM	Schwarz Pharma	Polymeric nanosphere	Verapamil hydrochloride	1998	Treatment of hypertension	[348]
53	Zevalin	Spectrum Pharms	Polymeric conjugate	Ibritumomab tiuxetan	2002	Treatment of adult patients with relapsed or refractory, low‐grade or follicular B‐cell non‐Hodgkin's lymphoma	[349]
54	Zilretta	Flexion Theraps Inc.	Polymeric nanogel	Triamconolone acetonide	2017	Indicated for the management of osteoarthritis pain in the knee	[350]
**Nanocrystals**							
55	Apretude	Viiv Healthcare Co.	Nanocrystal	Cabotegravir	2021	Used as a HIV‐1 pre‐exposure prophylaxis in adults and adolescents who weigh at least 35 kg	[351]
56	Aristada Initio	Alkermes Inc.	Nanocrystal	Aripiprazole lauroxil	2018	Used as a one‐time injection (with oral aripiprazole) to initiate/restart Aristada treatment for schizophrenia	[352]
57	Avinza	King Pharma	Nanocrystal	Morphine sulfate	2002	For the management of moderate to severe pain when continuous and prolonged opioid analgesia is required	[353]
58	Azopt	Novartis	Nanocrystal	Brinzolamide	1998	Treatment of increased intraocular pressure in adults with open‐angle glaucoma or ocular hypertension	[354]
59	Cabenuva	Viiv Healthcare Co.	Nanocrystal	Cabotegravir	2021	Treatment of HIV infection in adults and adolescents aged 12 years and older	[355]
60	Cesamet	Eli Lilly and Co.	Nanocrystal	Nabilone	2009	Management of nausea and vomiting associated with cancer chemotherapy	[356]
61	Elixophyllin	Nostrum Labs Inc.	Nanocrystal	Theophylline	1979	Treatment of reversible airflow obstruction associated with chronic asthma and emphysema	[357]
62	Emend	Merck	Nanocrystal	Aprepitant	2003	Used in the prevention of chemotherapy‐induced nausea and vomiting in cancer patients	[358]
63	Focalin XR	Novartis	Nanocrystal	Dexmethylphenidate hydrochloride	2005	Treatment of attention deficit hyperactivity disorder	[359]
64	Gris‐Peg	Novartis	Nanocrystal	Griseofulvin	1982	Treatment of various ringworm infections, including ringworm of the body and athlete's foot	[360]
65	Invega Hafyera	Janssen Pharmaceuticals Inc.	Nanocrystal	Paliperidone palmitate	2021	Treatment of schizophrenia in adults after they have been adequately treated with a once‐monthly paliperidone palmitate formulation	[361]
66	Invega Sustenna	Janssen Pharmaceuticals Inc.	Nanocrystal	Paliperidone Palmitate	2009	Indicated for both the acute and maintenance treatment of schizophrenia in adults	[362]
67	Invega Trinza	Janssen Pharmaceuticals Inc.	Nanocrystal	Paliperidone palmitate	2015	It is a three‐month injection indicated for the treatment of schizophrenia	[363]
68	IVEmend	Merck	Nanocrystal	Fosaprepitant	2008	Used to prevent acute and delayed nausea and vomiting associated with cancer treatment	[364]
69	Megace ES	Endo Pharms Inc.	Nanocrystal	Megestrol acetate	2005	Treatment of anorexia or cachexia in patients with a diagnosis of AIDS	[365]
70	Naprelan	Wyeth Pharmaceuticals	Nanocrystal	Naproxen sodium	1996	Used to treat pain/inflammation caused by conditions such as arthritis, ankylosing spondylitis, tendinitis, bursitis, and acute gout	[366]
71	Ostim	AAP biomaterials	Nanocrystal	Nanocrystalline hydroxyapatite	2004	Bone substitute for filling in bone defects and fractures	[367]
72	Rapamune	Wyeth Pharmaceuticals	Nanocrystal	Rapamycin/sirolimus	1999	Indicated for the prophylaxis of organ rejection in patients receiving renal transplants	[368]
73	Ritalin LA	Novartis	Nanocrystal	Methylphenidate hydrochloride	2002	Treatment of attention deficit hyperactivity disorder	[369]
74	Ryanodex	Eagle Pharmaceuticals	Nanocrystal	Dantrolene sodium	2014	Treatment of malignant hyperthermia in conjunction with appropriate supportive measures	[370]
75	Tricor	AbbVie pharmaceuticals	Nanocrystal	Fenofibrate	2004	Treatment of Hypercholesterolemia	[371]
76	Triglide	Skyepharma	Nanocrystal	Fenofibrate	2005	Treatment of Hypercholesterolemia	[372]
77	Vitoss	Orthovita	Nanocrystal	Beta‐tricalcium phosphate	2003	Bone substitute for filling in bone defects and fractures	[373]
78	Zanaflex	Covis Pharma	Nanocrystal	Tizanidine hydrochloride	2002	It is a muscle relaxant used to treat muscle spasms by reducing muscle stiffness	[374]
79	Zyprexa	Eli Lilly and Co.	Nanocrystal	Olanzapine	2000	Treatment of certain mental/mood disorders such as schizophrenia, bipolar disorder, and depression	[375]
**Inorganic Nanoparticles**							
80	Dexferrum	American Regent Inc.	Metallic nanoparticles	Iron dextran	1996	Treatment of patients with iron deficiency in cases where oral administration is unsatisfactory	[376]
81	Feraheme	AMAG Pharmaceuticals	Metallic nanoparticles	Ferumoxytol	2009	Treatment of iron deficiency anemia in adult patients with chronic kidney disease	[377]
82	Ferrlecit	Sanofi Aventis	Metallic nanoparticles	Sodium ferric gluconate	1999	Treatment of iron deficiency anemia in adult patients and pediatric patients aged 6 years and older	[378]
83	Hensify	Nanobiotix	Metallic nanoparticles	Hafnium oxide	2019	Interaction of ionizing radiation and hafnium promotes higher energy deposition, thereby enhancing radiotherapy	[378]
84	INFeD	Allergan	Metallic nanoparticles	Iron dextran	1974	Treatment of iron deficiency anemia in patients who are unable to tolerate oral iron supplementation	[379]
85	Injectafer	American Regent Inc.	Metallic nanoparticles	Ferric carboxymaltose	2013	Treatment of iron deficiency anemia in adult patients with chronic kidney disease	[380]
86	Monofer	Pharmacosmos	Metallic nanoparticles	An iron molecule with unbranched carbohydrate iron particles	2010	Treatment of iron deficiency anemia	[381]
87	Nanocis	CIS Bio	Metal‐based colloid	Chloride dehydrates	2000	It is a radiopharmaceutical product for lymphography and gastroesophageal reflux studies	[382]
88	Nanocoll	GE Healthcare	Metal‐based colloid	Albumin and stannous	1995	Bone marrow scintigraphy and inflammation scintigraphy in areas other than the abdomen. It is used as a radiopharmaceutical for sentinel node localization in breast cancer	[383]
89	PolyMem	Ferris Mfg. Corp.	Metallic nanoparticles	Silver	1988	Silver nanoparticles incorporated in dressings are designed to facilitate wound healing, relieve pain, and reduce inflammation	[384]
90	Somatuline Depot	Ipsen Pharma	Nanotube	Lanreotide acetate	2007	Used for the long‐term treatment of patients with acromegaly and gastroenteropancreatic neuroendocrine tumors	[385]
91	Venofer	American Regent Inc.	Metallic nanoparticles	Iron sucrose	2000	Treatment of iron deficiency anemia in adult and pediatric patients (2 years and older) with chronic kidney disease	[386]

## 4 Regulatory Landscape of Nanomedicines

The bench‐to‐bedside development process for nanomedicines encapsulates a series of meticulously orchestrated stages (**Table** **3**). The process begins with the discovery and design phase, where novel nanoparticles are conceptualized and synthesized. This is followed by preclinical evaluation, involving rigorous in vitro and in vivo tests to assess safety and efficacy. Next, manufacturing development scales up production while maintaining strict quality control. The regulatory submission phase then presents comprehensive data to governing bodies, a crucial step before advancing to clinical trials, where the safety and efficacy of the nanomedicine are tested in human subjects. Upon successful trials, regulatory approval is sought, leading to commercialization and, ultimately, patient application, where the nanomedicine reaches those in need.

**Table 3:** Roadmap of nanomedicine development process.

Stage	Key Activities	Outputs/Goals	Key Challenges
1.Discovery and Design	Target identification Nanomaterial design Preliminary in vitro testing Molecular modeling and simulation	Defined therapeutic target Optimized nanoparticle properties Initial proof of concept Validated computational model	Ensuring biocompatibility Achieving optimal drug loading and release Integrating computational predictions with empirical data Early‐stage failures
2. Preclinical Evaluation	Testing in animal models Stability characterization Pharmacokinetics Biodistribution Toxicology evaluation Genetic profiling of disease models	Demonstrated preclinical efficacy and safety Initial pharmacokinetic data Toxicity profile Detailed biodistribution profiles	Translating animal data to humans Managing variability across models Achieving desired drug release in vivo Ethical considerations
3. Manufacturing Development	Process optimization GMP compliance Quality control Real‐time analytics for monitoring during manufacturing	Scalable manufacturing process Clinical‐grade production Regulatory‐compliant quality controls Automated production process	Maintaining product stability Ensuring reproducibility at scale High manufacturing costs Integrating new manufacturing technologies
4. Regulatory Submission	Identify regulatory pathways Submit clinical trial applications Compile dossiers Engage with regulatory agencies	IND or equivalent regulatory approval Regulatory acceptance of preclinical and manufacturing data Streamlined submission process	Navigating complex regulations Adapting to evolving regulatory expectations Delays in approvals Compiling comprehensive data
5. Clinical Trials	Conduct Phase I, II, III trials Utilize adaptive trial designs	Safe dosing profile Proven clinical efficacy Positive safety and side effect profile Faster trial progression	Recruiting suitable patients Managing high costs and time investment Balancing rapid progression with thorough safety assessment Risk of trial failures
6. Regulatory Approval	Submit New Drug Application Gain market approval Develop post‐marketing study commitments	Market authorization Regulatory approval for clinical use Conditional approvals with further study commitments	Managing post‐approval risks Addressing real‐world safety concerns Complying with ongoing regulatory obligations
7. Commercialization	Product launch Marketing, distribution Post‐marketing surveillance Digital marketing strategies Patient education	Successful market entry Real‐world usage in clinical settings Feedback for product improvement Higher market penetration	Competition in the market Scaling production Navigating digital marketing regulations Maintaining patient privacy
8. Patient Application	Treatment implementation Personalized treatment Monitoring and feedback through mobile health apps	Improved patient outcomes Tailored therapy for specific conditions Continuous monitoring and adjustments Real‐time data‐driven refinements	Real‐world implementation challenges Managing data security and patient consent Monitoring for unexpected side effects

Among these stages, regulatory submission stands out as particularly challenging. This phase not only requires an exhaustive compilation of data across multiple developmental facets but also demands precise alignment with the evolving regulatory standards for nanotechnology. The complexity is heightened by the novel properties of nanoparticles, which may behave unpredictably within biological systems, thus necessitating additional scrutiny and sometimes novel regulatory considerations. This stage is pivotal, acting as a gatekeeper that determines whether a promising nanomedicine can transition into clinical use, thereby shaping the trajectory of its clinical and commercial future.

### 4.1 Need for Regulatory Oversight

Nanomedicine holds immense promise for revolutionizing healthcare, yet it grapples with a pressing need for regulatory oversight. Owing to their intricate interactions with genetic materials and biomolecules, they pose inherent risks of genotoxicity and mutagenicity.^[^ ^387^, ^388^ ^]^ These risks stem from the inflammatory responses triggered by nanomedicines, culminating in the production of reactive oxygen and nitrogen species that induce oxidative and nitrosative stress. The resultant accumulation of free radicals can inflict multifaceted damage, ranging from oxidative DNA damage to protein denaturation, lipid peroxidation, and even carcinogenesis and fibrosis.^[^ ^389^ ^]^ Intravenous administration exacerbates these concerns, evidenced by the liver accumulation and translocation of nanoparticles to critical bodily systems like the central nervous, cardiovascular, and renal systems.^[^ ^390^ ^]^ Despite the mounting evidence of potential harm, the lack of standardized regulatory guidance exacerbates the uncertainties surrounding nanomedicines. The intricate interplay between nanoparticle properties and biological systems complicates the assessment of physicochemical and toxicological characteristics, impeding regulatory decision‐making.^[^ ^391^ ^]^ Each nanomedicine's unique properties, including nanoparticle type, surface properties, administration route, and morphology, necessitate tailored regulatory approaches. The caution exercised by regulatory agencies is warranted, underscored by past instances where market‐approved nanoparticles, such as Sinerem and Cliavist, were later withdrawn due to unforeseen adverse events.^[^ ^392^ ^]^

Moreover, the environmental ramifications of nanomedicine utilization demand meticulous scrutiny. Concerns regarding the disposal and production of nanomedicines highlight the need for robust environmental risk assessments.^[^ ^393^ ^]^ Due to their small size and unique chemical properties, nanoparticles may persist in the environment longer than traditional pharmaceuticals, potentially leading to bioaccumulation and ecological disruption.^[^ ^394^ ^]^ However, global regulatory agencies are grappling with formulating comprehensive guidelines despite mounting pressure. The absence of definitive regulatory frameworks not only hampers clarity for manufacturers, healthcare providers, and policymakers but also stifles innovation and public acceptance. Without adequate guidance, uncertainties persist, impeding funding, research, development, and eventual commercialization of nanomedicines. While strides have been made, the path toward comprehensive nanomedicine regulation remains fraught with challenges, underscoring the imperative for concerted global collaboration and regulatory harmonization to safeguard public health and environmental integrity.^[^ ^395^ ^]^ Besides, bridging the gap between scientific advancements and regulatory frameworks necessitates interdisciplinary collaboration. Stakeholders, including scientists, clinicians, regulators, industry representatives, and ethicists, must engage in dialogue to establish transparent and adaptable regulatory pathways.

### 4.2 U.S FDA

The FDA, a federal agency tasked with safeguarding public health, regulates a wide array of products, such as food, drugs, medical devices, vaccines, biologics, and cosmetics. It is the primary authority overseeing the development, evaluation, and approval of nanomedicine products in the United States, ensuring they meet rigorous safety, efficacy, and quality standards before reaching patients and healthcare providers.^[^ ^396^ ^]^ As a global leader in nanotechnology regulation, the FDA collaborates internationally to establish common standards and guidelines. The FDA issues guidance documents and policy statements that provide industry stakeholders with clear recommendations and regulatory expectations for developing and evaluating nanomedicine products. These cover aspects like characterization, safety assessment, manufacturing processes, and regulatory submissions specific to nanotechnology‐based products.^[^ ^397^ ^]^ Adhering to these guidelines promotes transparency, streamlines regulatory processes, and facilitates the timely approval and commercialization of nanomedicine products, thereby advancing patient care and treatment outcomes. The following section delves into crucial insights extracted from the “Nanotechnology Guidance Documents” provided by the US FDA.

- Guidance for Industry: Considering Whether an FDA‐Regulated Product Involves the Application of Nanotechnology^[^ ^398^ ^]^

Issued on June 24, 2014, this guidance document offers recommendations for discerning the use of nanotechnology in medical products. It delineates factors for assessing nanomaterial properties and their regulatory implications. Notably, the FDA underscores that these recommendations are non‐binding and do not establish legal obligations. The document highlights two key considerations: particle dimensions and dimension‐dependent properties. These factors aid in determining if FDA‐regulated products utilize nanotechnology. According to this guidance, the FDA will assess whether an FDA‐regulated product employs nanotechnology by considering two factors:

- Whether the material or end product is designed to have dimensions in the nanoscale range (≈1  –100 nm), either externally, internally, or on its surface.
- Whether the material or end product is engineered to display properties or effects, such as physical, chemical, or biological, that are influenced by its dimensions, even if these dimensions extend beyond the nanoscale range, up to one micrometer (1000 nm).

The term “engineered” distinguishes intentionally manipulated products using nanotechnology from those containing naturally occurring nanoscale materials. FDA's interest in such engineered materials or products with nanoscale dimensions or related properties differs from substances like microorganisms or proteins, which exist naturally at small scales. It also distinguishes deliberately manipulated nanotechnology products from those unintentionally including nanoscale materials. The phrase “material or end product” encompasses various FDA‐regulated articles, including finished products like drug tablets and materials intended for use in them, such as additives during processing. According to Point 1, if a material or end product is designed to have dimensions ranging from 1  to 100 nm, the FDA will assess any unique characteristics or biological effects it may have on safety, effectiveness, public health, or regulatory status. This point also includes any aggregates or agglomerates formed by such nanoscale particles. Additionally, point 1 covers coated, functionalized, or hierarchically‐assembled engineered structures containing discrete and functional nanoscale entities internally or on the surface. These entities, whether embedded or attached to the surface, fall under Point 1. Point 2 sets an upper limit of 1000 nm and involves dimension‐dependent properties or phenomena akin to those observed in materials with nanoscale dimensions. This serves as an initial screening tool to identify materials or products with properties relevant for regulatory review.

This guidance underscores the importance of product‐specific premarket reviews for gaining insight into the characteristics and behavior of nanotechnology‐based products. In cases where such products aren't reviewed before market entry, the FDA encourages industry players to engage with the Agency early in the development phase. This ensures that any concerns regarding regulatory status, safety, effectiveness, or public health impact are addressed effectively.

- Guidance for Industry: Safety of Nanomaterials in Cosmetic Products^[^ ^399^ ^]^

Published on June 15, 2020, this guidance document presents suggestions for assessing the safety of nanomaterials utilized in cosmetics. It delves into considerations regarding the characterization, toxicological assessment, risk evaluation, and labeling of cosmetic products incorporating nanomaterials. The guideline underscores that the safety of typical cosmetic products can be sufficiently validated by drawing upon existing toxicological data on individual ingredients and comparable product formulations, alongside conducting additional tests as necessary. However, it highlights the potential for nanoscale cosmetic materials to exhibit altered physicochemical properties, behaviors, and effects compared to larger‐scale counterparts of the same composition, necessitating further safety inquiries.

In line with the guidelines’ recommendations, ensuring safety entails comprehensive characterization of nanomaterials and examination of various physical and chemical attributes, including impurity content. Notably, particular attention must be paid to agglomeration and size distribution throughout toxicity testing and in the final product. A thorough understanding of nanomaterial toxicology and their behavior in cosmetics mandates scrutiny of exposure routes, uptake, absorption, and toxicity. Furthermore, when assessing toxicity testing methods for cosmetics containing nanomaterials, their unique properties and biological interactions must be considered.

It is imperative to adapt traditional toxicity testing methods or develop novel ones to address key chemical and physical properties that may influence the toxicity profile of nanomaterials and their impact on cosmetic formulation efficacy. Toxicological assessments should encompass ingredient and impurity toxicity, dosimetry for in vitro and in vivo studies if applicable, clinical testing if deemed necessary, and consideration of toxicokinetics and toxicodynamics. The guideline also underscores the importance of addressing in vitro and in vivo toxicological data on nanomaterial ingredients and impurities, as well as conducting studies on dermal penetration, potential inhalation, skin and eye irritation, sensitization, mutagenicity, and genotoxicity.

The guideline outlines testing methodologies considered at the time of publication, which can be further refined for specific nanomaterials to ascertain ingredient safety. These methods include utilizing reconstructed human skin models like Episkin TM and Epiderm TM for skin irritation and corrosion testing, phototoxicity testing via 3T3 NRPT (3T3 fibroblasts neutral red uptake phototoxicity testing) for UV‐absorbing substances, employing human/pig skin in diffusion cells for dermal absorption studies, employing Bovine Corneal Opacity and Permeability (BCOP) and Isolated Chicken Eye (ICE) assays for ocular irritation, and conducting genotoxicity tests encompassing gene mutation and structural and numerical aberrations, while accounting for the nanomaterial's specific properties to understand its genotoxic effects.

- Guidance for Industry: Liposome Drug Products^[^ ^400^ ^]^

Issued in April 2018, this guideline explores the necessary data and details required for potential applicants submitting a new drug application (NDA) or abbreviated new drug application (ANDA) for a liposome‐based medication under review by the Center for Drug Evaluation and Research at the FDA. The FDA underscores that the scientific principles delineated in this guidance might similarly pertain to liposome drug products intended for market under biologics license applications. These principles can serve as guidance throughout drug development, potentially culminating in the submission of an investigational new drug application. The guideline covers the following topics:

i) Chemistry, manufacturing, and controls (CMC): The section provides exhaustive guidance for the development, characterization, and regulation of liposome‐based pharmaceuticals. It mandates a thorough description of the drug product components, including the drug substance, lipids, nonlipid components of the liposome, and non‐liposome inactive ingredients. Additionally, it emphasizes expressing the composition of each component, detailing ranges in composition and attributes based on product development studies. The physicochemical properties critical for characterizing liposome drug formulations are meticulously outlined, encompassing morphology, surface characteristics, net charge, drug encapsulation efficiency, particle size, phase transition temperature, in vitro drug release, leakage rate, and liposome integrity changes. These properties are fundamental in ensuring the quality, stability, and efficacy of liposome drug products.

Furthermore, the guidance identifies critical quality attributes (CQAs) specific to liposome formulations, such as vesicle/particle size, size distribution, and morphology, aligning with international pharmaceutical development standards. In terms of manufacturing, the guidance underscores the importance of providing a detailed description of the manufacturing process, including unit operations, process parameters, and controls. It emphasizes the need for process validation to ensure consistency and reproducibility before commercial distribution, addressing challenges unique to liposome products, such as batch‐size‐related hold times and sterilization. Control of lipid components is also emphasized, with recommendations for characterization, manufacture, and specification of lipid components, covering both synthetic and naturally sourced lipids. Detailed descriptions of lipid composition, manufacturing processes, specifications, and stability assessments are deemed crucial to ensuring product quality and safety.

Recommendations are provided for establishing drug product specifications, encompassing physicochemical parameters, drug content, degradation products, residual solvents, in vitro drug release, sterility, and pyrogenicity. These specifications are vital for maintaining consistency and quality throughout the manufacturing process. The guidance also outlines requirements for stability studies to evaluate the microbiological, physical, and chemical stability of liposome drug products, including liposome integrity, lipid, and drug substance stability. Stress testing and accelerated stability studies are highlighted for assessing degradation profiles and establishing appropriate storage conditions. Lastly, considerations for post‐approval changes in manufacturing, such as formulation modifications, site changes, and equipment upgrades, are discussed, emphasizing the complexity and sensitivity of liposome formulations and the need for prior approval supplements to maintain product quality and efficacy.

ii) Human pharmacokinetics and bioavailability (bioequivalence in the case of an ANDA): This section delves into the intricacies of clinical pharmacology studies, biopharmaceutics, bioanalytical methods, and liposome‐protein interactions, providing a comprehensive framework for the evaluation of liposome‐based pharmaceuticals.

Clinical pharmacology studies play a pivotal role in understanding the pharmacokinetics and dosing regimens of liposome drug products. These studies should encompass various parameters, including area under the plasma concentration‐time curve (AUC), peak plasma concentration, elimination half‐life, and volume of distribution, among others. Recommendations are provided for conducting multiple‐dose studies, dose‐proportionality studies, and exposure‐response studies to gather comprehensive pharmacokinetic data. Additionally, considerations for specific patient populations and potential drug interactions are addressed, ensuring the relevance and applicability of study outcomes. Comparative clinical pharmacology studies with non‐liposome drug products are emphasized to elucidate differences in drug disposition and pharmacokinetics between liposome and non‐liposome formulations. This comparative analysis aims to discern differences in absorption, distribution, metabolism, and excretion (ADME) between the two formulations. Particularly, when a non‐liposome product is approved and available for comparison, conducting these studies provides valuable insights into the bioequivalence and performance of liposome formulations.

Biopharmaceutics considerations focus on establishing the release characteristics of liposome products and correlating in vitro findings with in vivo outcomes. Demonstrating that the release characteristics of the liposome product meet label claims is paramount, along with describing any disparities between liposome and non‐liposome products with the same active ingredient. While establishing a complete in vitro/in vivo correlation (IVIVC) may pose challenges, attempts to establish in vitro/in vivo relationships (IVIVRs) are encouraged to enhance understanding of product behavior.

Bioanalytical methods employed in pharmacokinetic studies should be rigorously validated to ensure accurate quantification of both liposome‐contained and free drug substances. These methods play a crucial role in evaluating the pharmacokinetics and bioavailability of liposome drug products, providing essential data for regulatory submissions. Liposome‐protein interactions are highlighted due to their potential impact on drug release and pharmacological properties in vivo. The guidance suggests that prior studies on protein‐liposome interactions may suffice for new liposome drug products if the lipid composition and physicochemical characteristics align with previously studied formulations.

iii) Labeling in NDAs and ANDAs: This section offers detailed directives on the content of labeling specifically tailored for liposome drug products. This ensures comprehensive and accurate communication of essential information to healthcare professionals and patients alike. FDA underscores the significance of utilizing the established nonproprietary name for liposome drug products, typically derived from the United States Pharmacopeia (USP) drug product monograph title. In instances where a USP monograph is absent, adherence to guidelines outlined in 21 CFR 299.4 and USP Nomenclature Guidelines is mandated. The recommended nonproprietary name should incorporate terminology denoting the product as a liposome or a pegylated liposome, thereby facilitating unambiguous identification. Sequential assignment of types (e.g., Type A, Type B) is advised for subsequent products of the same drug and dosage form.

The guideline advocates the inclusion of a cautionary note, aiming to highlight potential variances in behavior between liposome and non‐liposome drug products, notwithstanding identical active ingredients. Unless determined therapeutically equivalent by the FDA, applicants are encouraged to elucidate such distinctions. Labeling should encompass a statement cautioning against substituting liposome drug products for non‐liposome counterparts or other liposome products containing the same active ingredient, unless therapeutically equivalent as determined by the FDA. Comprehensive instructions for reconstitution, if applicable, and statements delineating the appropriate in‐use period should be provided for both unloaded liposomes and products where the drug substance is loaded during manufacturing. Additionally, instructions for products labeled for mixing with other approved drug products should be included, alongside stipulations regarding storage conditions for reconstituted drugs and considerations for robustness under varied reconstitution conditions.

- Guidance for Industry: Drug Products, Including Biological Products, that Contain Nanomaterials^[^ ^401^ ^]^

Issued recently in April 2022, this guideline provides guidance on the development of human drug products, including those that are biological products, in which a nanomaterial (as explained in this section) is present in the finished dosage form. It discusses both general principles and specific considerations for the development of drug products containing nanomaterials, including considerations for developing products through abbreviated pathways. Considerations for quality, nonclinical, and clinical studies are discussed as they relate to drug products containing nanomaterials throughout product development and production. The guideline covers the following topics:

i) Risk‐based framework (Potential risk factors for products with nanomaterials): This section of the guidance provides a comprehensive examination of the considerations essential for evaluating the risks associated with drug products incorporating nanomaterials. It acknowledges the inherent diversity among such products, encompassing variations in administration routes, therapeutic indications, nanomaterial functions, structural complexities, and technological maturity levels. Nanomaterials exhibit distinct chemical, physical, and biological properties compared to their larger counterparts, potentially influencing the quality, safety, and efficacy of drug products.

For instance, nanomaterials may alter dissolution rates, bioavailability, distribution profiles, and residence times within the body. Their interaction with plasma proteins can lead to the formation of a protein corona, affecting their pharmacokinetics and biodistribution. Nanomaterials can be passively or actively targeted to specific sites in the body, leveraging size, charge, or surface modifications. Passive targeting relies on intrinsic properties, while active targeting involves surface functionalization with specific molecules recognized by receptors. Understanding these interactions is crucial, considering factors such as intrinsic (e.g., disease, age) and extrinsic (e.g., co‐administered drugs) variables that may influence exposure and response.

The FDA recommends a risk‐based approach, emphasizing the need for adequate characterization of nanomaterials and understanding their intended use and application in relation to product quality, safety, and efficacy. The framework outlines several key risk factors for assessment, including the adequacy of nanomaterial characterization, complexity of nanomaterial structure, mechanistic understanding of physicochemical properties and biological effects, predictability of in vivo release, physical and chemical stability, maturity of nanotechnology (including manufacturing and analytical methods), potential impact of manufacturing changes on critical quality attributes (CQAs), and the physical state of the material upon administration and route of administration.

Manufacturers are encouraged to utilize accumulated information over the product lifecycle to minimize residual uncertainty. The level of characterization and understanding should be justified in alignment with the risk‐based approach outlined in the guidance, ensuring comprehensive assessment and mitigation of potential risks associated with drug products containing nanomaterials.

ii) Quality Recommendations: This section provides a detailed framework for ensuring the quality of drug products containing nanomaterials, addressing critical aspects of characterization, manufacturing, stability, and postmarket changes. An exhaustive description of the nanomaterial(s) within the product is paramount, encompassing essential characteristics such as size, charge, morphology, composition, and complexation, tailored to the product's life cycle stage. Notably, mere ingredient lists may fall short in explaining complex nanomaterial structures, necessitating narrative descriptions and structural diagrams to provide a comprehensive understanding. Functionality descriptions are crucial, detailing how nanomaterials are utilized in the product, such as for solubilization, carrier functions, or targeting.

Quality attributes and structural characterization play pivotal roles, with emphasis on determining critical quality attributes (CQAs) relevant to function and product performance. Parameters like chemical composition, particle size, distribution, morphology, and stability are imperative for assessment. Additionally, in vitro release methods should discriminate formulation and manufacturing differences that impact product performance, with validation protocols tailored to the product's characteristics. Manufacturing processes must adhere to current good manufacturing practice (cGMP) standards, considering the complexity and scale dependency of nanomaterial‐containing products. Adequate controls should be established early to prevent cross‐contamination and ensure batch consistency. Changes in analytical methods, process scale, and manufacturing sites may complicate batch bridging, highlighting the need for sample retention and continual process improvement.

Excipients play crucial roles in nanomaterial‐based formulations, impacting product attributes and performance. Their material attributes must be fully characterized, with proper controls defined in the premarket application. Safety assessments are essential, particularly when excipients are deliberately modified into nanomaterials, requiring consultation with regulatory authorities. Stability considerations extend beyond traditional factors, including nanomaterial‐specific risks like changes in size, morphology, and surface properties. In‐use stability studies may be necessary to assess interactions with dilution media and primary packaging. Postmarket changes to nanomaterial‐containing products require careful evaluation, potentially necessitating physicochemical comparisons and in vivo bioequivalence studies.

iii) Nonclinical studies for drug products: This section provides insights into the preclinical assessment of drug products incorporating nanomaterials. It emphasizes aligning with established International Council for Harmonization (ICH) and FDA guidelines while acknowledging the need for tailored approaches due to nanomaterial characteristics. Notably, nonbiodegradable nanomaterials may exhibit prolonged persistence, potentially leading to chronic exposure‐related effects, necessitating thorough absorption, distribution, metabolism, and excretion (ADME) studies. To accurately assess biodistribution, nanomaterial labeling (e.g., radiolabeling, fluorescence) is often required for enhanced in vivo detection.

Moreover, specific administration routes pose unique safety considerations. Topically applied products may enhance skin penetration or distribution to local lymph nodes, requiring meticulous evaluation of exposure and effects. Subcutaneous administration could heighten sensitization potential, while inhalation routes demand scrutiny of local toxicity and lung deposition. Intravenous products may alter tissue distribution and half‐life, necessitating thorough evaluation. Similarly, oral products may require assessment of increased bioavailability and potential tissue accumulation, particularly for insoluble nanomaterials.

Before conducting toxicity studies, ensuring nanomaterial reproducibility and representativeness is paramount. Understanding the factors influencing nanomaterial properties in vitro and in vivo is critical, with analytical methods tailored and validated for nanomaterial characteristics. Additionally, when modifying previously approved drug products to include nanomaterials, ADME and bridging toxicology studies are often necessary. These studies can build upon findings from the original product, incorporating nanomaterial‐related changes and potential toxicity contributions. Overall, the guidance underscores the importance of meticulous nonclinical assessment to ensure regulatory compliance and mitigate potential risks associated with drug products containing nanomaterials.

iv) Clinical development: This section offers detailed insights into the regulatory framework and methodological considerations for evaluating drug products containing nanomaterials in clinical settings, aligning with established guidelines for IND, NDA, ANDA, and BLA submissions. Specifically tailored to products developed through the 505(b)(2), 505(j), or 351(k) pathways, this section emphasizes the crucial role of pharmacokinetic‐pharmacodynamic (PK‐PD) assessments, distinguishing between nanomaterials serving as therapeutic agents or carriers. For 505(b)(2) submissions, the guidance delves into nuanced PK‐PD considerations based on the role of nanomaterials in the product. It underscores the importance of assessing exposure‐response relationships, considering potential disparities in disposition and therapeutic performance compared to conventional products lacking nanomaterials. The guidance advocates for a risk‐based approach to evaluate potential changes in exposure, safety, and effectiveness, with products categorized into low, medium, and high‐risk tiers based on nanomaterial characteristics and administration routes.

Clinical studies for 505(b)(2) submissions are outlined, with low‐risk products potentially relying on comparative plasma PK to establish bioequivalence, while medium and high‐risk products necessitate comprehensive PK, PD, and tolerability assessments. Notably, bridging the performance of proposed products to listed drugs may require additional nonclinical and clinical evidence, particularly for medium and high‐risk categories. Moreover, clinical trials should carefully select appropriate endpoints, considering both therapeutic and toxicity‐related biomarkers, to ascertain exposure‐response relationships accurately. In the context of 505(j) submissions, demonstrating bioequivalence between generic nanomaterial‐containing products and reference‐listed drugs (RLD) poses unique challenges. The guidance underscores the necessity of comprehensive in vivo PK evaluations and in vitro physicochemical characterizations, particularly for nanomaterial‐containing active ingredients, excipients, and complex formulations. Various physicochemical tests, such as particle morphology and drug release profiles, are recommended for robust bioequivalence assessments.

For 351(k) submissions concerning biosimilars, applicants must thoroughly assess the nanomaterial's contributions to product safety, purity, and potency. Early engagement with regulatory authorities is encouraged, with a focus on evaluating immunogenicity risks throughout product development and lifecycle stages. The guidance emphasizes specific assessments regarding nanomaterials' potential adjuvant properties and their implications for immunogenicity. Additionally, bioanalytical methods must effectively differentiate between total, free, and nanomaterial‐associated drug entities, necessitating sensitive and specific assays. In vitro tests with human biomaterials, including stability, biocompatibility, plasma protein binding, and clearance/metabolism evaluations, are crucial for understanding nanomaterial interactions and potential cytotoxicity.

v) Environmental Impact Considerations: This section underscores the regulatory obligations and nuances in evaluating the environmental ramifications of drug products containing nanomaterials. It underscores the National Environmental Policy Act's (NEPA) mandate for Federal agencies to assess the environmental effects of their actions and ensure public awareness. FDA mandates applicants to submit either an Environmental Assessment (EA) or a claim of categorical exclusion when seeking Agency action on premarket applications for drugs or biologics, in accordance with 21 CFR 25.15(a). CDER and CBER adopt a tailored approach, evaluating on a case‐by‐case basis whether drug products with nanomaterials warrant categorical exclusion or necessitate an EA due to evolving scientific understanding.

The evaluation process for an EA involves meticulous scrutiny to verify its accuracy, objectivity, and potential significant impacts on the human environment. If significant effects that require an Environmental Impact Statement (EIS) are identified, FDA prepares an EIS accordingly, adhering to its procedures as outlined in 21 CFR 25.15(b). Conversely, if no significant effects are identified, FDA issues a Finding of No Significant Impact, as prescribed by 21 CFR 25.41.

To streamline decision‐making and minimize late‐cycle requests for information, FDA advises industry stakeholders to notify the agency early in the development process regarding their intent to claim a categorical exclusion or submit an EA. If claiming a categorical exclusion, applicants must provide substantive evidence supporting the selected exclusion criteria and a statement confirming the absence of extraordinary circumstances, as required by 21 CFR 25.15(a). Such evidence may include comprehensive data demonstrating minimal release of nanomaterials into the environment or evidence indicating negligible toxicity to aquatic and terrestrial organisms at expected exposure levels.

In instances where FDA identifies extraordinary circumstances, applicants are compelled to submit at least an EA, which must comprehensively assess the bio‐persistence, exposure, fate, and risk of nanomaterials in the environment, as stipulated by 21 CFR 25.21. Additionally, if the proposed action is deemed to significantly impact the human environment, necessitating an EIS, FDA may request further information from the applicant to facilitate the analysis. It is emphasized that environmental impacts may manifest across various stages of the product life cycle, including manufacturing, storage, patient use, and disposal.

Based on the above‐mentioned discussion on guidelines, it is clear that the FDA employs a nuanced regulatory approach towards products containing nanomaterials, grounded in a science‐based framework tailored to their diverse nature and applications. This approach prioritizes product‐specific evaluations, recognizing the relationship between nanomaterial properties and their mechanical and biological contexts. It acknowledges variations in legal standards across different product classes, where safety remains paramount, but evaluation criteria may vary.^[^ ^402^ ^]^ Premarket review processes are vital for FDA oversight, requiring manufacturers to submit comprehensive data on safety, efficacy, and regulatory status. For products like nanotechnology‐based cosmetics, where premarket review isn't mandatory, the FDA relies on available data sources and postmarket surveillance. Consultations with the FDA prior to market entry are encouraged, particularly for nanotechnology applications, facilitating safety reviews and postmarketing surveillance. Postmarket monitoring is integral, enabling swift action to address emerging safety concerns and ensuring ongoing vigilance. The FDA continuously monitors the marketplace for products containing nanomaterials, intervening when necessary to safeguard consumers.^[^ ^403^ ^]^

The FDA underscores the importance of fostering collaboration with industry stakeholders, thereby promoting early engagement to elucidate regulatory obligations and enhance adherence to compliance standards. Moreover, the FDA actively engages with other governmental bodies and international counterparts to harmonize policies and facilitate the dissemination of regulatory intelligence. Notably, the Emerging Technologies Interagency Policy Coordination Committee serves as a pivotal platform for intergovernmental policy discourse, fostering a unified regulatory framework and fostering the exchange of best practices on a global scale.^[^ ^404^ ^]^ In parallel, specialized institutions, such as the Nanotechnology Characterization Laboratory of the National Cancer Institute (NCL‐NCI), have played a significant role in advancing the regulatory oversight of nanomedicines, leveraging over a decade of dedicated contributions.^[^ ^405^ ^]^

To address the regulatory challenges posed by nanotechnology, the FDA has established the Nanotechnology Task Force and Nanotechnology Interest Group, comprising representatives from various regulatory bodies. Their collective mandate is to address the complexities of nanotechnology regulation on a global scale.^[^ ^406^ ^]^ Through their deliberations, the Task Force has determined that existing regulatory frameworks sufficiently encompass the safety considerations pertinent to nanomedicine production. This determination is predicated on the premise that the current regulatory protocols, including pre‐market testing and approval processes under the NDA framework, are robust enough to identify and mitigate potential toxicities associated with nano‐based products.^[^ ^407^ ^]^ Despite this acknowledgment, the FDA has maintained its regulatory stance, applying existing guidelines designed for conventional pharmaceuticals to nanomedicines without substantive alteration. This apparent inertia in regulatory adaptation has elicited significant criticism directed toward the FDA, particularly in light of the evolving landscape of nanotechnology.

**Table** **4** provides a brief overview of the above‐discussed FDA documents offering key insights into the regulatory considerations for nanotechnology in medical and cosmetic products.

**Table 4:** FDA guidance on nanotechnology applications in regulated products.

Guidance Title	Issued On	Scope	Key Considerations	Recommendations and Implications
Considering Whether an FDA‐Regulated Product Involves the Application of Nanotechnology	June, 2014	This guidance aims to help manufacturers determine if their products can be considered as utilizing nanotechnology according to FDA regulations.	Nanoscale measurements: 1‐100 nm in at least one dimension. Properties or phenomena attributable to dimensions smaller than 1000 nm that affect performance.	Manufacturers should document the presence of nanomaterials and their intended functionalities. The FDA encourages consultations during development to ensure regulatory compliance. While the guidance is non‐binding, using it can aid in passing regulatory review more smoothly, influencing safety, efficacy, and marketability evaluations.
Safety of Nanomaterials in Cosmetic Products	June, 2020	Outlines safe practices for incorporating nanomaterials in cosmetics, covering risk assessment, production, and product labeling.	Comprehensive material characterization. Detailed toxicological profile necessary due to altered properties of materials at the nanoscale. Regular monitoring for stability and agglomeration of particles.	Conduct rigorous safety testing, including repeated exposure assessments and post‐market surveillance. Label products to inform consumers of nanomaterial presence and safety testing. Compliance ensures consumer safety and helps avoid legal challenges or market recalls. It also fosters consumer confidence in product safety.
Liposome Drug Products	April 2018	Provides specific guidelines for the design, manufacture, and approval of liposome‐based drugs, focusing on quality and stability.	Detailed characterization of liposomes and their components. Critical quality attributes must be controlled within specified limits. Emphasis on stability, drug encapsulation, and release profiles.	Strict adherence to specified manufacturing processes and controls. Required comprehensive testing for stability and drug release over intended shelf life. Recommend ongoing process validation to ensure product consistency. Necessary for securing FDA approval. Non‐compliance could result in significant delays in approval or direct refusal.
Drug Products, Including Biological Products, that Contain Nanomaterials	April 2022	Covers a broad range of drug products that incorporate nanomaterials, detailing their development, regulatory considerations, and post‐market expectations.	Risk assessment must consider nanoscale effects on pharmacokinetics and pharmacodynamics. In‐depth study of interactions between nanomaterials and biological systems. Evaluations of long‐term stability and potential toxicity.	Manufacturers should utilize advanced analytical methods to fully characterize nanomaterials. Engagement with FDA early in the development process is recommended to ensure alignment with regulatory expectations. Ensures comprehensive assessment of safety and efficacy, directly affecting the product's market success and compliance with health regulations.

### 4.3 Other Global Regulatory Bodies

Apart from the FDA, numerous regulatory bodies, predominantly located in developed economies, have assumed the responsibility of formulating regulatory frameworks for nanomedicine.

#### 4.3.1 European Medicines Agency

EMA, based in Amsterdam, is an integral arm of the European Union (EU), entrusted with evaluating, supervising, and ensuring the safety of medicines intended for use within the EU. Its responsibilities encompass scrutinizing applications for marketing authorization of medicines, offering scientific guidance to pharmaceutical firms, and overseeing pharmacovigilance efforts to monitor the safety of medicines post‐market release.^[^ ^408^ ^]^ Acknowledging the distinct opportunities and challenges posed by nanomedicines due to their innovative characteristics, the EMA has repeatedly recognized the growing significance of nanotechnology in healthcare.^[^ ^409^ ^]^ Consequently, it has devised scientific guidelines (published as “reflection papers”) to assist medicine developers in preparing marketing authorization applications for human medicines. These guidelines have undergone periodic updates to incorporate fresh insights, thereby refining their objectives. Below, we delve into the latest iterations of these guidelines:

- Data requirements for intravenous liposomal products developed with reference to an innovator liposomal product^[^ ^410^ ^]^: This document serves as a guideline for generating essential data to support the marketing authorization of intravenous liposomal products. It addresses critical aspects such as pharmaceutical quality, non‐clinical evaluation, and clinical studies necessary to establish comparability with innovator products.

Of paramount importance is the pharmaceutical comparability between the applicant's formulation and its innovator counterpart, necessitating a nuanced comprehension of liposomal formulations. These formulations exhibit intricate behaviors owing to interactions among lipids, drug substances, and physiological milieus. The identification of key parameters for quality characterization, including liposome morphology, mean size, size distribution, and encapsulation efficiency, assumes significance. Additionally, stability studies under proposed in‐use conditions are imperative to ensure batch‐to‐batch consistency, encompassing assessments of lipidic component characteristics, impurity profiles, and stability under diverse storage conditions. Methodologies for quality characterization must adhere to regulatory directives, ensuring robustness and reproducibility.

EMA mandates comparative investigations of pharmacokinetics, pharmacodynamics, and toxicity to establish equivalence with the innovator product. Particular emphasis must be placed on the selection of suitable animal models and analytical techniques for quantifying encapsulated and unencapsulated drug levels in plasma and tissues. Non‐clinical pharmacokinetic studies necessitate evaluating drug release kinetics from liposomes and their impact on systemic exposure and tissue distribution. Rigorous attention to sample sizes, study durations, and endpoints is indispensable for robust clinical evaluation. Analytical methodologies play a pivotal role in quantifying drug levels in liposomal products, commonly employing high‐performance liquid chromatography coupled with mass spectrometry for this purpose. Thorough method validation is requisite to ensure accuracy and reliability while being sensitive to changes in liposomal attributes and drug release rates under varying physiological conditions.

To establish pharmaceutical comparability, comparative stress tests are recommended to be conducted to assess physical and chemical degradation under diverse conditions, ensuring alignment of critical quality attributes with the innovator product. Toxicological evaluations are crucial for delineating the safety profile of liposomal products, necessitating meticulous assessment to mitigate risks associated with acute infusion reactions. In vitro and in vivo immune reactogenicity assays are recommended to gauge potential adverse events, alongside organ function tests to evaluate target organ toxicity. A comprehensive evaluation of safety profiles, encompassing acute and chronic toxicity assessments, immunotoxicity, and potential organ‐specific adverse effects, is advised.

Clinical studies constitute an indispensable component for assessing efficacy and safety in human subjects. The document recommends comparative pharmacokinetic assessments that establish equivalence in drug exposure, distribution, and elimination profiles, while efficacy trials should be implemented to validate therapeutic equivalence through randomized controlled trials (with judicious consideration for dose selection and study design). Lastly, safety evaluations that vigilantly monitor acute infusion reactions and other adverse events will be applied. Post‐marketing surveillance and pharmacovigilance activities are indispensable for continual monitoring of safety and effectiveness in real‐world settings.

- General issues for consideration regarding parenteral administration of coated nanomedicine products^[^ ^411^ ^]^: This paper delivers crucial insights for the development and regulatory oversight of nanomedicine formulations endowed with coatings tailored for parenteral administration. These coatings represent a critical interface between engineered nanomedicine surfaces and the biological milieu, exerting profound influences on their behavior, therapeutic efficacy, and safety profile post‐administration. Noteworthy is the multifaceted functionality of these coatings, whether non‐covalently adhered or covalently bound, ranging from enhancing stability and mitigating aggregation to prolonging circulation time and facilitating disease‐specific targeting. However, their presence introduces complexities and considerations necessitating meticulous assessment. Through a thorough understanding of coating effects and adherence to these guidelines, stakeholders can ensure the safety, efficacy, and regulatory compliance of coated nanomedicine products throughout their lifecycle.

The EMA forwards the following directives for the evaluation of coated nanomedicine:

- Thorough characterization of the coating material encompassing its composition and quality control measures;
- Additional characterization for ensuring consistency and reproducibility, especially if the coating agent or the addition of a targeting ligand entails a complex molecule (e.g., protein or antibody);
- Rigorous validation of the coating process, delineating the chemistry underpinning the adherence of non‐covalent coatings or the conjugation of covalently bound coating materials;
- Evaluation of the potential impact of surface coverage heterogeneity on the safety and efficacy of the product;
- Accurate depiction of the orientation and conformational state of any ligand, particularly in products featuring an active targeting moiety on the surface;
- Comprehensive assessment of the coating's stability during storage and upon application;
- In vitro appraisal of the physicochemical stability of the coating concerning its intended use under conditions reflective of the route of administration, pharmacokinetics, bio‐distribution, and target pathology;
- Examination of potential ramifications on efficacy and safety in the event of premature detachment or degradation of the coating, leading to exposure of new functional groups on the nanomedicine surface;
- Consideration of the in vivo repercussions of different coating materials and surface coverage on pharmacokinetics and bio‐distribution;
- Assessment of the bio‐distribution and metabolic fate of released coating material.

- Development of block copolymer micelle medicinal products:^[^ ^412^ ^]^ This paper provides insights into the pharmaceutical development, non‐clinical evaluations, and initial clinical assessments of block‐copolymer micelle drug formulations. The active agent may comprise a diverse range, spanning low molecular weight compounds, nucleic acids, or entities of biological or biotechnological origin, such as peptides and proteins. Given the intricate nature of these systems, particularly regarding the chemical binding of the active substance and the potential use of additional stabilizers, the EMA advocates for early engagement with developers to discuss the critical attributes specific to each block copolymer micelle product.

In accordance with this guideline, pivotal regulatory requisites encompass the thorough characterization of block copolymer micelle formulations, validation of manufacturing processes, and demonstration of product stability throughout its shelf life. Additionally, regulatory submissions mandate detailed documentation of analytical methodologies employed for quality control and batch‐to‐batch consistency assessment. The critical quality attributes of block copolymer micelle products encompass diverse parameters pertaining to their composition, morphology, stability, and drug loading, necessitating meticulous characterization through a repertoire of analytical techniques, including DLS, transmission electron microscopy, zeta potential measurement, and spectroscopic methods. Furthermore, validation of these analytical methodologies is imperative to ascertain their precision, accuracy, and reproducibility.

This document underscores the importance of formulating a well‐defined manufacturing process alongside requisite process controls to ensure the consistent production of acceptable products. Manufacturers are required to furnish comprehensive documentation pertaining to the synthetic pathway, purification procedures, and specifications for starting materials. Identification and control of critical process parameters such as temperature, pH, and mixing speed are crucial for optimizing micelle formation and product quality. Any alterations in critical manufacturing process parameters or equipment necessitate a thorough characterization of the block copolymer micelle product on a case‐by‐case basis.

Non‐clinical pharmacokinetic investigations mandate the development of validated analytical techniques capable of accurately quantifying both total and free active substance concentrations across diverse biological matrices and organs. In vivo pharmacokinetic assessments should encompass parameters including Cmax, half‐life, and AUC for both total and free active substances, considering the influence of physicochemical attributes such as micelle size, surface charge, and morphology on distribution. Moreover, elucidating the fate of micelle products and their constituents, including metabolites, is imperative for a comprehensive understanding of their pharmacokinetic behavior. Concurrently, non‐clinical pharmacodynamic appraisals should utilize both in vitro and in vivo models to delineate pharmacological responses, taking into account the impact of micelle properties on the fate and distribution of the active substance. Adherence to established safety pharmacology guidelines is pivotal for identifying potential toxicities and interactions, while toxicology assessments should comprehensively evaluate the toxicological profile and exposure‐response relationships. Organ‐specific evaluations may be warranted based on the physicochemical and pharmacokinetic characteristics of the micelle product and its constituents.

- Data requirements for intravenous iron‐based nano‐colloidal products developed with reference to an innovator medicinal product^[^ ^413^ ^]^: This document provides a thorough outline of the necessary data for regulatory approval of intravenous iron‐based nano‐colloidal products. Like its liposome counterpart, it emphasizes the significance of demonstrating comparability with an innovator product through a comprehensive assessment of quality, non‐clinical, and clinical data. Intravenous iron‐based nano‐colloidal products represent a significant advancement in the treatment of iron deficiency. However, ensuring their similarity to an innovator product requires a robust “weight of evidence” approach, incorporating data from multiple sources.

Parameters such as stability of the iron‐carbohydrate complex and physicochemical properties of the carbohydrate matrix are deemed critical for efficacy and safety. Specific quality standards and analytical methods for characterizing these products are outlined, including considerations for starting materials and degradation kinetics. Analytical methods for quantifying analytes in blood/plasma and tissue are deemed necessary, with a focus on bio‐distribution studies to evaluate comparability. Studies should be conducted in compliance with Good Laboratory Practice (GLP) and include assessments of distribution, metabolism, and excretion in relevant animal models.

Pharmacokinetic studies comparing the test product with the innovator's product are recommended, utilizing single‐dose parallel or crossover designs. Primary endpoints include AUC and Cmax of total‐ and transferrin‐bound iron, with baseline correction to decrease variability. The guideline states that further therapeutic equivalence studies may not be necessary if data from quality, non‐clinical, and pharmacokinetic studies demonstrate similarity. Safety endpoints, including hypersensitivity reactions and markers of iron overload, should be monitored, and risk management plans should be implemented.

In EU, significant strides have been undertaken beyond the examination of reflection papers. Task forces and consortiums have been mobilized to delineate the precise definition of “nanomaterial,” supplemented by a plethora of reports and recommendations aimed at harmonizing the regulation of nanomedicine.^[^ ^414^ ^]^ Noteworthy endeavors include the establishment of initiatives such as the European Nanomedicine Characterization Laboratory (EU‐NCL), in collaboration with the United Kingdom, and the REFINE project, both of which have received governmental funding to propel advancements in this domain.^[^ ^415^ ^]^ Nevertheless, it is crucial to acknowledge that these initiatives are currently situated within the realm of public consultation. At present, no formal regulatory guidance has been implemented.

#### 4.3.2 Other Regulatory Bodies

Health Canada has established a comprehensive Working Definition of Nanomaterials, which delineates its classification criteria based on size and associated phenomena. Specifically, a material is deemed a nanomaterial if it falls within the nanoscale range (1–100 nm) in at least one dimension or if it exhibits nanoscale properties despite being larger or smaller in all dimensions.^[^ ^416^ ^]^ In the context of regulatory approval for nanotechnology products, Canada relies upon pre‐existing regulatory frameworks. Health Canada strongly advises manufacturers to engage with the appropriate regulatory authorities early in the development process to evaluate the risks and properties of their products thoroughly. To facilitate this process, the Health Portfolio Nanotechnology Working Group has been established, comprising representatives from key regulatory bodies such as Health Canada and the Canadian Institutes of Health Research. Furthermore, Health Canada has issued general guidance concerning nanotechnology‐based health products and food items. To effectively assess the potential risks and benefits of nanotechnology, manufacturers are urged to request pre‐submission meetings with regulatory authorities.^[^ ^417^ ^]^ These discussions aim to elucidate the necessary information for safety assessments, which may include, but are not limited to: the intended application of the nanomaterial (encompassing its utilization in end products); details regarding manufacturing methods employed; comprehensive characterization of the nanomaterial (including its identity, composition, and purity); toxicological, eco‐toxicological, metabolic, and environmental fate data, which may pertain either generally or specifically to the nanomaterial; and strategies for risk assessment and management, where applicable or implemented.^[^ ^418^ ^]^

In Japan, medicinal products are subject to regulation overseen by the Ministry of Health, Labor and Welfare (MHLW) and the PMDA. Within this regulatory framework, nanomedicines are notably governed under the broader auspices of the pharmaceutical affairs law, lacking a defined or specific framework tailored explicitly for them. Their evaluation appears to follow a case‐by‐case methodology. Despite the absence of a formalized definition or framework, Japan has established a Nanomedicine Initiative Working Group tasked with deliberating on the regulatory requirements pertinent to nanomedicine advancement.^[^ ^419^ ^]^ Collaboration between PMDA and MHLW is evident in the development of regulatory documents. For instance, they jointly produced a reflection paper on block copolymer micelle medicinal products. Furthermore, PMDA contributed to the creation of management guidance documents related to clinical trial notifications for specific nanotechnology‐based medicines. In 2016, MHLW issued several key documents aimed at providing guidance in the development of nanotechnology‐based drug products. These include a reflection paper concerning nucleic acids (siRNA)‐loaded nanotechnology‐based drug products and a guideline for the development of liposome drug products.^[^ ^420^, ^421^ ^]^ Such initiatives signify Japan's proactive stance in addressing the regulatory considerations inherent in the burgeoning field of nanomedicine.

In various Asian regions, notably India, China, and Thailand, the governance and regulatory frameworks concerning nanotechnology remain nascent. However, these countries are actively engaged in formulating policies to address the burgeoning concerns associated with this field. In India, for instance, the Department of Biotechnology unveiled guidelines in 2019 aimed at evaluating nanomedicine.^[^ ^422^ ^]^ These guidelines endeavor to categorize nanomedicine based on factors such as degradability and the inherent properties of the nanomaterial or the nanoform of the active ingredient. Moreover, the guidelines delineate a comprehensive set of data requirements to be submitted to regulatory agencies when seeking approval for clinical trials. Specifically, the guidelines mandate the submission of physicochemical characterization data for nanomedicine, with certain parameters designated as Critical Quality Attributes (CQAs), which include criteria for unique identification, impurity profiling, and stability assessment. The guidelines also emphasize the necessity of furnishing in vitro and in vivo release kinetics of the active drug or ingredient, as well as degradation kinetics of the nanomedicine across various simulated environments. This approach offers a valuable framework for ensuring the rigorous evaluation of nanomedicine and nanocarriers in clinical trials. Nevertheless, it is acknowledged that the specific data requirements for nanomedicine evaluation may vary on a case‐by‐case basis, aligning with the evolving regulatory landscape and scientific understanding of this domain.

### 4.4 Potential Impact of Nanomedicine Attributes on Regulatory Decision‐Making

The lack of comprehensive guidelines in the realm of nanomedicine is unmistakable, with existing directives often found to be rudimentary and inadequate in addressing the intricate challenges inherent in regulating nanomedicines, especially when contrasted with conventional drug formulations. However, amid this deficit, it is noteworthy that the guidelines discussed in the preceding section underscore a paramount focus on defining and elucidating Critical Quality Attributes (CQAs).

From a technical standpoint, CQAs encompass distinct physical, chemical, biological, or microbiological traits or parameters crucial for guaranteeing the quality of pharmaceuticals, biotechnological products, or other regulated items. Their significance lies in their direct influence on the safety, effectiveness, and overall performance of the product. Identifying CQAs involves a meticulous examination of the product and its manufacturing process, typically conducted during the developmental and validation phases.^[^ ^423^ ^]^ These attributes play a fundamental role in establishing the quality benchmarks necessary for ensuring product consistency and regulatory adherence. A paramount aspect of CQAs is their unequivocal definition and measurability, which ensures they accurately represent the indispensable characteristics of the product without ambiguity or subjectivity.^[^ ^424^ ^]^ This precision is vital in ensuring that each attribute faithfully reflects the intended quality aspect of the product. Failure to control CQAs within defined parameters could lead to a product that either fails to fulfill its intended function or poses risks to patient well‐being. Moreover, it's important to note that CQAs are often influenced by Critical Process Parameters (CPPs), further stressing their interconnection within the manufacturing framework.^[^ ^425^ ^]^

Recent efforts have been directed toward identifying regulatory imperatives and optimizing static communication in the realm of nanomedicine. A survey was conducted to pinpoint regulatory experiences with nanomedicines, discern regulators' informational requirements for nanomaterial categorization and characterization, and outline future strategies conducive to the acceptance of nanotechnology‐driven products.^[^ ^426^ ^]^ The findings underscored regional disparities in nanomedicine marketing expertise, emphasizing the necessity for inter‐agency knowledge exchange and collaboration to effectively navigate the burgeoning landscape of nanotechnology applications. However, embarking on such a collaborative trajectory necessitates a foundational framework of consistent terminology and nanomedicine categorization, essential for fostering seamless communication and cooperation among regulatory entities. Despite proposals for nano‐specific characteristic lists to guide clinical trial approvals, there remains a conspicuous lack of alignment among regulatory bodies regarding the identification of pivotal characteristics. This discordance poses challenges for sponsors striving to compile comprehensive datasets for international clinical trial submissions, impeding progress in multi‐regional approval processes.^[^ ^427^ ^]^ The overarching conclusion is clear: an urgent imperative exists for precisely characterizing physicochemical properties through appropriate analytical methodologies for toxicity assessments, encompassing both in vitro and in vivo testing. Paramount to ensuring the quality of nanomedicines is a concerted effort towards elucidating CQAs and enhancing understanding thereof.

Nanomedicine necessitates a nuanced approach toward CQAs, which can vary depending on factors like nanomaterial type, delivery mechanism, therapeutic goal, and intended application. Yet, amidst this diversity, certain fundamental CQAs consistently emerge as pivotal benchmarks for evaluating nanomedicine efficacy and safety. These encompass particle size distribution, surface charge, surface functionalization, drug loading and release dynamics, biocompatibility and toxicity profiles, stability and shelf life, targeting precision, manufacturing process parameters, and in vivo performance metrics.^[^ ^428^ ^]^ In the intricate landscape of regulatory scrutiny surrounding nanomedicine products, CQAs play a central role, guiding pivotal decisions across the product lifecycle. Regulatory agencies undertake rigorous risk assessments to discern potential hazards associated with nanomedicine, with CQAs serving as indispensable navigational aids in this process. Specifically, they furnish comprehensive insights into the physicochemical characteristics and biological interactions of nanoparticles. For instance, data on particle size distribution, surface charge, and functionalization offer crucial clues regarding potential toxicity, immunogenicity, and off‐target effects.^[^ ^429^ ^]^ This deep comprehension of risks empowers regulatory bodies to delineate robust risk management strategies, be it by establishing acceptable exposure thresholds or mandating supplementary safety evaluations to counter identified hazards.

Establishing robust quality control standards rooted in CQAs stands as a cornerstone in ensuring the unwavering quality and performance of products. By instituting rigorous quality control measures, developers can effectively mitigate variations in product attributes, thereby guaranteeing uniformity across batches.^[^ ^430^ ^]^ Regulatory bodies meticulously evaluate these standards to ascertain that nanomedicine products adhere to predefined quality benchmarks, thus ensuring minimal risks to patient safety and efficacy. A thorough characterization of nanomedicine formulations emerges as a foundational prerequisite for regulatory sanction. CQAs act as pivotal markers of both product quality and performance, furnishing regulators with indispensable data to gauge safety and efficacy.^[^ ^431^ ^]^ For instance, an exhaustive examination of particle size distribution, surface charge, drug loading, and release kinetics empowers regulators to assess the physicochemical properties, stability, and therapeutic potential of the product.^[^ ^432^ ^]^ A robust characterization underlines the developer's adept comprehension of CQAs and their proficiency in manufacturing a product that is consistent and of superior quality.

Modifications in manufacturing protocols, formulation components, or manufacturing sites carry the potential to significantly influence CQAs and subsequent product performance. Regulatory bodies mandate thorough comparability assessments by developers to evaluate the ramifications of such alterations on product quality and safety.^[^ ^433^ ^]^ These assessments necessitate careful scrutiny of CQAs, both pre‐ and post‐changes, to substantiate product consistency and equivalence. Regulatory bodies ascertain the product's sustained safety and efficacy profile across its lifespan by inspecting pivotal attributes such as particle dimensions, surface features, and drug release kinetics.^[^ ^434^ ^]^ In clinical development, understanding CQAs is crucial for designing well‐controlled studies that evaluate the safety and efficacy of nanomedicine products. Developers must incorporate CQAs into study protocols to ensure reliable and interpretable results. Robust characterization of CQAs enhances the scientific rigor and validity of clinical trial data, facilitating regulatory decision‐making^[^ ^435^ ^]^

Thorough characterization of CQAs stands as a cornerstone in the submission of regulatory dossiers. A thorough exposition of essential characteristics, underpinned by rigorous characterization studies, empowers regulators to gauge a product's adherence to regulatory standards and its eligibility for market approval.^[^ ^436^ ^]^ The comprehensive documentation of CQAs not only facilitates the regulatory evaluation process but also accelerates market entry for developers.^[^ ^437^, ^438^ ^]^ Lastly, sustained vigilance over CQAs through post‐market surveillance activities proves indispensable in ensuring the enduring safety of nanomedicine formulations. Regulatory bodies may mandate the establishment of pharmacovigilance programs by developers to promptly detect and evaluate alterations in critical attributes or unforeseen adverse events linked to the product.^[^ ^439^ ^]^ The continuous monitoring of CQAs equips regulators to pinpoint emerging safety issues, evaluate product performance under real‐world conditions, and enact appropriate regulatory measures to safeguard public health.^[^ ^440^ ^]^

## 5 Recommendations for Better Clinical Translation

### 5.1 Preclinical Evaluation

#### 5.1.1 In Vitro Evaluation of Nanoparticles

In vitro release testing is vital for quality control and predicting the in vivo performance of drug delivery systems. Although it has been a standard quality control test since its adoption by the United States Pharmacopeia in 1970, no standardized in vitro dissolution or release test currently exists for nanoparticulate systems. Developing suitable testing methods for these systems is challenging due to difficulties in rapidly and efficiently separating nanoparticles from the release medium, the complexity of the drug release mechanisms, and the diverse nature of nanoparticulate systems, some of which are designed to release the drug only after uptake into target cells.^[^ ^441^ ^]^ Achieving precise control over the drug release kinetics of nanoparticles is critical for maximizing therapeutic efficacy while minimizing toxic side effects. However, current techniques, such as UV–vis spectrophotometry, cannot directly measure drug release from nanoparticles due to technical challenges in distinguishing between the released drug in the medium and the drug retained within the nanoparticles. To address this, various methods have been employed, categorized into discontinued methods, such as the sample and separate method, and continuous methods, like membrane diffusion methods.^[^ ^442^, ^443^ ^]^ The sample and separate method involves sampling at predetermined times, separating the released drug from the particles through filtration or centrifugation, and then assaying the drug using conventional techniques.^[^ ^444^ ^]^ However, these methods can disrupt sensitive nanoparticles, cause continued drug release during separation, and face issues like filter clogging or breakage. Tangential flow filtration (TFF) minimizes these problems by reducing membrane clogging and shear stress, making it an efficient option for nanoparticle purification and drug separation.^[^ ^445^ ^]^ The membrane diffusion method, which uses a dialysis membrane to isolate particles from the release medium, has been widely adopted.^[^ ^446^, ^447^, ^448^ ^]^ This method typically involves two compartments‐ a donor compartment containing the nanoparticle formulation and a receiver compartment with a large volume of release medium, separated by a dialysis membrane. The measured drug release kinetics reflect a combination of the drug's release from nanoparticles into the donor medium and its subsequent diffusion across the membrane into the receiver.^[^ ^449^ ^]^ However, standardizing this method is challenging due to variations in laboratory practices, dialysis membrane characteristics, and testing conditions like volume of release media, stirring rate, and flow rate. Moreover, a mathematical tool is still required to separate and analyze the two‐step drug transport process to accurately evaluate the actual drug release kinetics of nanoparticles within dialysis devices.^[^ ^450^ ^]^

Recent advancements have significantly enhanced the methods used to analyze drug release from nanoformulations. For example, a study demonstrated the efficacy of the NanoDis System in characterizing the release profile of poly(lactic‐co‐glycolic) (PLGA) nanocapsules loaded with all‐trans retinoic acid. The NanoDis System integrates a USP II dissolution apparatus (paddle) with an autosampler and uses tangential flow filtration (TFF) to separate nanoparticles from the dissolution medium. The system pumps the dissolution medium through TFF filters, which retain the nanoparticles while allowing the filtrate, free from particles, to be collected by the autosampler. This method effectively separates nanoparticles from dissolved active pharmaceutical ingredients (APIs) within minutes, irrespective of particle size, due to the precise molecular weight cut‐off of the filters. The NanoDis System demonstrated superiority over the traditional dialysis method by accurately characterizing the burst release from the nanocapsules and distinguishing between different nanoparticle formulations of all‐trans retinoic acid.^[^ ^445^ ^]^ A recent study introduced a novel sample and separate method by combining the USP apparatus II (paddle) with centrifugal ultrafiltration. This approach efficiently separated free drug from nanoparticles, providing accurate and repeatable in vitro release kinetics. Compared to traditional methods, such as the dialysis membrane method and sample and separate methods using syringe filters, the sample and separate + centrifugal ultrafiltration technique proved superior. The dialysis membrane method was found to misestimate release kinetics due to factors like nanoparticle stability, surface properties, and the release medium. Similarly, the syringe filter‐based sample and separate method failed to adequately separate free drug from nanoparticles, resulting in a pseudo‐burst release effect.^[^ ^451^ ^]^ Several alternative approaches have been explored for in vitro release testing. Electrochemical methods allow for rapid in situ measurements and help avoid interference from undissolved dosage forms. Differential pulse polarography has been reported as effective for assessing electroactive drugs. In addition to these, several non‐electrochemical techniques have been investigated, including calorimetry, turbidimetry, and laser diffraction, each providing valuable insights into drug release profiles. Additionally, methods like microdialysis, vertical diffusion Franz cells, and in vitro lipolysis models have been developed to enhance the understanding of drug release from complex formulations.^[^ ^452^ ^]^

Mathematical modeling and numerical simulations have been explored to predict drug release kinetics in systems with multiple transport resistances, such as nanoparticles in dialysis bags or sphere assemblies in confined spaces with limited medium volume. While these sophisticated methods can be performed by specialized laboratories, they are not easily applicable for routine use due to the complexity of the required release mechanisms and model parameters. Zhou et al. proposed a novel method utilizing numerical deconvolution to evaluate the actual drug release kinetics of nanoparticles within the donor compartment. This approach relied on experimental release profiles of nanoparticles and free drug solutions in the receptor, as determined by existing dialysis tests. For the first time, this simple and efficient mathematical tool based on numerical deconvolution was used to accurately quantify drug release from nanoparticles inside the donor compartment.^[^ ^450^ ^]^

#### 5.1.2 Stability

Nanoparticles are defined as materials with at least one dimension in the range of 1–100 nm.^[^ ^453^, ^454^ ^]^ This size scale imparts unique properties that differ from bulk materials. As materials transition from bulk to nanoscale, their surface area‐to‐volume ratio and surface energy increase significantly. Due to these high surface energies, nanoparticles are in a metastable state, less stable compared to bulk materials under standard conditions of room temperature and pressure.^[^ ^455^ ^]^ This metastability is a key factor in nanoparticle behaviour and is central to discussions on nanoparticle stability and size‐dependent properties. Research into these phenomena is extensive and continues to grow, highlighting the importance of understanding nanoparticle stability in various applications.^[^ ^456^ ^]^ Stabilizing these particles often requires surface modifications, such as PEGylation, to prevent aggregation and ensure consistent therapeutic outcomes. As nanoparticle formulations become more complex, ensuring their stability during both storage and administration becomes critical for maintaining their efficacy.

#### 5.1.3 Biocompatibility and Toxicity

Nanoparticles interacting with cells and the extracellular environment can induce a range of biological effects, influenced by their dynamic physicochemical properties. Characteristics, such as size, chemical composition, shape, and surface coating, significantly impact the biocompatibility and efficacy of nanoparticles, especially in drug delivery systems.^[^ ^457^ ^]^ The uptake of nanoparticles dictates their internalization route and subsequent fate in the body. High biocompatibility is achieved when nanoparticles interact with biological systems without eliciting toxic, immunogenic, thrombogenic, or carcinogenic responses, making this a key design consideration in drug delivery applications.^[^ ^458^ ^]^ Given the diverse preparation methods and resulting properties of nanoparticles, their interactions with cells and tissues can vary widely. Nanoparticles can permeate cell membranes, traverse nerve cell synapses, and spread through blood vessels and lymphatic systems. They may also selectively accumulate in certain cell types or cellular structures, which can enhance drug delivery efficacy but also pose potential health risks.^[^ ^459^ ^]^ Currently, there are no standardized biocompatibility evaluation criteria specifically for nanoparticles, necessitating a case‐by‐case assessment specific to the tissue type and intended application. Therefore, a thorough evaluation of the factors influencing nanoparticle biocompatibility and toxicity is essential for their safe and sustainable development.

Studies have focused on evaluating the blood compatibility of nanoparticles through various in vitro experiments, including blood cell aggregation, hemolysis, and coagulation behaviour assessments. Among these, hemolysis is considered a simple and reliable measure for estimating the blood compatibility of materials.^[^ ^459^ ^]^ This approach involves analyzing the extent of red blood cell destruction caused by nanoparticles, which helps determine their potential impact on blood components and overall biocompatibility.^[^ ^460^, ^461^, ^462^ ^]^

Although nanoparticulate systems offer considerable promise for treating various human diseases, they can pose serious risks if there are unanticipated changes in product quality or performance, potentially leading to toxicity or altered efficacy. Consequently, developing standardized testing methods for these novel drug delivery systems is crucial. This is especially important given the rapidly increasing number of nanotechnology‐driven products entering the market and the drug development pipelines.^[^ ^441^ ^]^ Ensuring consistent product performance and quality is essential to fully realize the benefits of these advanced therapies.

The absence of preclinical models that accurately represent human biology has led to a high rate of therapeutic failures in clinical trials, contributing to escalating healthcare costs and fewer effective drugs reaching patients.^[^ ^463^ ^]^ Additionally, increasing concerns about animal consciousness have intensified the debate over animal testing. To address these ethical issues, the principle of the 3Rs (reduction, refinement, and replacement) is used to promote more humane animal research.^[^ ^464^ ^]^ Both societal and governmental pressures are mounting to reduce animal testing. This shift is exemplified by the recent FDA Modernization Act 2.0, a landmark legislation that enables the approval of new therapies without the obligatory use of animals in safety and toxicology assessments.^[^ ^465^ ^]^ To avoid unethical practices, alternative strategies to animal testing have been implemented to address the limitations of traditional animal experiments.

Current alternatives to animal testing include cell‐based assays, tissue engineering, and computational methods for assessing nanotoxicity. Cell‐based assays and tissue engineering are in vitro techniques that enable the investigation of toxic effects within specific cellular environments through cell viability assays. While these methods are valuable for identifying potential toxicity, they do not fully replace the need for in vivo assessments. In silico approaches, such as molecular docking, quantitative structure‐activity relationship (QSAR) models, and molecular dynamics simulations, offer predictive tools for nanotoxicity studies. These methods explore the interactions between chemicals and biological systems, providing insights into the potential toxicological impacts of substances. Computational tools can predict key pharmacokinetic properties‐ such as absorption, distribution, metabolism, and excretion (ADME)‐ based on chemical structures. Despite their usefulness, in silico methods have limitations in accurately mimicking human physiology.^[^ ^464^ ^]^ There is an ongoing need for the development of more physiologically relevant models that can provide a comprehensive and systematic evaluation of nanoparticle toxicity. Enhanced predictive platforms are crucial for improving the accuracy of toxicity assessments and connecting the gap between in vitro and in vivo studies. Advances in these models could lead to more reliable safety evaluations and reduce the reliance on animal testing.

It has become increasingly evident that 3D cell culture systems offer a more accurate representation of in vivo cellular functions compared to traditional 2D monolayer cultures. This advantage is primarily attributed to the enhanced cell–cell and cell‐matrix interactions that 3D environments facilitate, closely mimicking the natural cellular microenvironment. These interactions significantly influence the physiological behavior and phenotype of individual cells, allowing them to exhibit more realistic proliferation rates, regain functions lost in monolayer cultures, and develop greater resistance to xenobiotic treatments. As a result, 3D cultures offer a more precise reflection of cellular responses, particularly in chronic toxicity studies, where their long‐term stability proves beneficial.^[^ ^466^, ^467^, ^468^ ^]^ Despite their evident superiority, 3D models are often more expensive, time‐consuming, and technically challenging, especially in data interpretation and experimental reproducibility.^[^ ^469^ ^]^ 3D cell cultures are broadly categorized into spheroids and organoids. Spheroids consist of clusters of differentiated cells that aggregate into tissue‐like structures, while organoids are multicellular constructs that mimic the structure and function of specific organs in vivo. Organoids, which offer a more dynamic and physiologically relevant in vitro model than monolayer cultures, have also gained prominence in nanoparticle toxicology.^[^ ^470^ ^]^ These miniaturized organs simulate the mechanical forces and environments that tissues and stem cells experience in vivo, providing a platform for more accurate toxicity assessments. The advantages of organoids include easier sample access and the elimination of ethical concerns associated with animal models.^[^ ^471^ ^]^ As a result, organoids have the potential to replace ex vivo models in specific scenarios, contributing to a broader understanding of human biology and addressing some limitations of animal models.

A comparative study on the toxicity of 20 nm SiO2 nanoparticles in 2D and 3D cell cultures revealed that while the nanoparticles induced toxicity in 2D cultures, this effect was not observed in 3D cultures, which more closely replicate in vivo conditions.^[^ ^469^ ^]^ Using a micropatterned agarose hydrogel platform, the effects of 25 nm ZnO nanoparticles on 3D colon cell spheroids were studied, revealing that cell dimensionality significantly influences outcomes such as inflammatory response and cytotoxicity. Notably, ZnO nanoparticles triggered different modes of cell death in 2D versus 3D cultures. In the 3D model, the outer cell layers provided only temporary protection to the inner cells before sloughing off. These findings suggest that 2D cell models may overestimate ZnO nanoparticle toxicity, while the 3D spheroid model offers a more accurate and reproducible platform for studying nanoparticle‐induced toxicity and mechanisms.^[^ ^472^ ^]^ The HepG2 spheroid model has proven effective for assessing nanoparticle (NP)‐induced cytotoxic and genotoxic effects, demonstrating that toxic responses can vary between 2D and 3D cultures. For instance, 3D cultures showed greater resistance to Ag‐NPs, whereas both 2D and 3D models exhibited strong cytotoxic effects with ZnO‐NPs and no effects with TiO2‐NPs. While 2D cultures provided clearer concentration‐dependent responses, 3D cultures, due to their complexity, had higher variability, making it more challenging to achieve statistically significant results. Nevertheless, the HepG2 spheroid model offers a more realistic representation of the human liver, given its more complex cell arrangements and exposure scenarios, making it a promising tool in nanotoxicology.^[^ ^473^ ^]^ Moreover, 2D cultures fall short in accurately representing the functions of 3D tissues, particularly regarding cell–cell and cell‐matrix interactions and diffusion/transport conditions. Consequently, cytotoxicity testing in 2D cultures may not fully capture the toxicity of nanoparticles and other nanostructures as they would manifest in the body.^[^ ^30^ ^]^ This limitation was further illustrated in a comparative nanotoxicological study where fibroblasts (L929) and melanoma (B16‐F10) cells were grown in both 2D and 3D arrangements. The study assessed cytotoxicity, reactive oxygen species (ROS) production, genotoxicity, cell morphology complexity, and the uptake of silver nanoparticles (AgNPs) and folic acid‐functionalized upconversion nanoparticles (FA‐UCNPs). It was found that AgNP cytotoxicity was higher in spheroids than in monolayer cultures, with elevated apoptotic cell percentages and ROS production in 3D cultures. Moreover, 2D cultures required twice the concentration of AgNPs to reach a considerable DNA damage index compared to 3D models, indicating a higher sensitivity in spheroids to genotoxic effects. FA‐UCNPs exhibited negligible toxicity in both 2D and 3D models. Additionally, AgNPs caused disaggregation and downsizing of spheroids in a concentration‐dependent manner, with 20% higher internalization of FA‐UCNPs observed in spheroids compared to 2D cell arrangements. These findings underscore the sensitivity of spheroids as a more accurate model for assessing nanoparticle biocompatibility and internalization.^[^ ^474^ ^]^

The absence of certain in vivo components, such as vasculature, in 3D cell culture models has driven the development of microphysiological systems, commonly referred to as organ‐on‐a‐chip technology.^[^ ^30^ ^]^ These systems integrate in vitro models with micro‐engineered environments and perfusion, creating controlled microenvironmental niches that enhance the physiological relevance of the models. The advent of organ‐on‐a‐chip has marked a significant advancement in preclinical assessment, enabling researchers to replicate complex biological interactions more accurately.^[^ ^475^, ^476^ ^]^ For instance, A novel 3D human lung‐on‐a‐chip model was developed to simulate the alveolar‐capillary barrier by co‐culturing endothelial and epithelial cells on a Matrigel membrane. This model enabled the evaluation of nanoparticle toxicity, including TiO2 and ZnO, through assessments of cellular morphology, junction protein expression, and ROS production. It provided a robust platform for nanotoxicity assessment and offered potential for future expansion into more complex, multi‐organ systems.^[^ ^477^ ^]^ However, these systems are not without limitations, particularly the absence of inter‐organ crosstalk present in the human body. To mitigate these challenges, multi‐organ‐on‐a‐chip devices have been developed, allowing multiple organs to be connected on a single chip via microfluidic channels, thus enabling more accurate simulations of physiological processes.^[^ ^478^ ^]^ Body‐on‐a‐chip systems, designed to replicate human physiological responses to drugs, offer the potential to assess both drug efficacy and toxicity across multiple organs.^[^ ^479^ ^]^ Using a microscale body‐on‐a‐chip system, researchers simulated the oral uptake of 50 nm carboxylated polystyrene nanoparticles, revealing liver injury at lower concentrations than single‐tissue models predicted. The multi‐tissue system demonstrated that nanoparticles could cross the intestinal barrier and induce liver damage, highlighting the model's relevance for evaluating nanoparticle effects on human tissues.^[^ ^480^ ^]^

Despite the promise these systems hold, their application in nanotoxicology is still in its early stages, with most studies remaining at the proof‐of‐concept level. The slow progress in this area can be attributed to several challenges, including the lack of standardized protocols for nanoparticle dosage, characterization, and analysis. These inconsistencies hinder the ability to compare results across different studies and platforms, limiting the broader adoption of organ‐on‐a‐chip systems in toxicology research.^[^ ^481^ ^]^

#### 5.1.4 In Vivo Preclinical Studies

Various nanodrug systems, including dendrimers, liposomes, micelles, and polymeric nanoparticles, are designed to encapsulate drugs, offering several pharmacokinetic benefits such as targeted delivery, enhanced metabolic stability, increased membrane permeability, improved bioavailability, and prolonged action.^[^ ^65^, ^482^, ^483^, ^484^, ^485^, ^486^ ^]^ The pharmacokinetics (PK) and tissue distribution of nanoparticles are key determinants of therapeutic efficacy and potential toxicity of nanoparticles. Critical factors such as nanoparticle size, surface charge, and surface chemistry play a significant role in influencing these PK and biodistribution profiles.^[^ ^487^ ^]^ Smaller nanodrugs tend to have higher transcellular uptake through follicle‐associated epithelia compared to larger ones. Nanoparticles typically enter cells via endocytotic mechanisms, including caveolar‐ and clathrin‐mediated endocytosis, and potocytosis. Larger particles, however, are more rapidly opsonized and cleared by the reticuloendothelial system (RES). Modifying the nanoparticle surface with hydrophilic polymers or surfactants, such as PEG or polysorbate 80 (Tween 80) to enhance circulation time and reduce opsonization. Nanoparticles around 100 nm in size typically exhibit extended circulation and reduced MPS uptake, while neutral nanoparticles tend to have even longer circulation times compared to those with a charge.^[^ ^488^ ^]^ Nanodrugs with positive surface charges can interact with negatively charged mucin, improving mucus penetration and epithelial cell internalization. Functionalization with membrane permeation enhancers or ligands can further facilitate transcellular transport. Additionally, bioadhesive polymers or chelators can be used to enhance paracellular transport by modulating tight junctions. Surface modification with specific proteins, antibodies, or biomolecules allows for targeted drug delivery, potentially improving therapeutic efficacy and reducing side effects, particularly in anticancer therapies.^[^ ^489^ ^]^ These characteristics collectively enhance the ability of nanoparticles to reach and remain in target tissues, thereby optimizing their therapeutic potential. Arvizo et al. investigated how the surface charge of gold nanoparticles influences pharmacokinetics, tumor uptake, and biodistribution of gold nanoparticles with various charges (neutral, zwitterionic, negative, and positive) and a consistent core size of 2 nm and overall diameter of 10 nm. It was observed that high systemic exposure and low clearance were provided by neutral and zwitterionic particles when administered intravenously, and rapid absorption into circulation was noted after intraperitoneal administration. Conversely, moderate systemic exposure was provided by negative particles, while positive particles were rapidly cleared and poorly absorbed intraperitoneally. This resulted in decreased tumor uptake for positive and negative particles in a xenograft model of ovarian cancer. Biodistribution studies in different mouse strains demonstrated that surface charge and administration route significantly influenced pharmacokinetics, organ distribution, and tumor targeting.^[^ ^490^ ^]^

Recent advancements in monitoring the biodistribution, pharmacokinetics, and pharmacodynamics of nanotherapeutics have significantly enhanced our understanding of their therapeutic efficacy and safety. Further, advanced imaging techniques, such as PET, MRI, and fluorescence imaging, allow for precise in vivo tracking of nanotherapeutics, providing detailed insights into their distribution and accumulation in tissues.^[^ ^491^, ^492^, ^493^ ^]^ Magnetic iron oxide nanoparticles, including Gd‐doped iron oxide nanoparticles (GdIONPs) with a 4 nm core size, have been used as T1‐weighted MRI contrast agents to quantify iron content in vivo. The study demonstrated that MRI T1 relaxivity of GdIONPs effectively matched in vitro relaxivity and ICP‐MS results, allowing accurate real‐time monitoring of iron levels at tumor sites.^[^ ^494^ ^]^ Ultrafine ORMOSIL nanoparticles (−20 nm) conjugated with a near‐infrared (NIR) fluorophore were synthesized by Kumar et al. for optical bioimaging, providing enhanced tissue penetration and reduced background signal. Additionally, Iodine‐124 was conjugated to the nanoparticles for positron emission tomography (PET), allowing bioimaging independent of tissue depth and accurate quantification of nanoparticle accumulation in major organs in vivo. Systemic injection in mice and subsequent biodistribution studies using fluorescence and gamma emission demonstrated that the nanoparticles were slowly cleared via hepatobiliary excretion without causing significant toxicity.^[^ ^495^ ^]^

Mass spectrometry‐based techniques, such as inductively coupled plasma mass spectrometry (ICP‐MS), matrix‐assisted laser desorption/ionization (MALDI)‐MS, nanoparticle‐assisted laser desorption/ionization mass spectrometry (LDI‐MS), and surface‐assisted LDI (SALDI)‐MS, offer high sensitivity and specificity for quantifying nanotherapeutics and their metabolites in biological samples, making them invaluable in pharmacokinetic studies. These techniques have been employed to investigate the pharmacokinetics and tissue distribution of various nanoparticles. For example, one study evaluated the pharmacokinetics and tissue distribution of magnetic iron oxide nanoparticles (Fe3O4 MNPs) in ICR mice using atomic absorption spectrophotometry, revealing widespread distribution with significant accumulation in the liver, spleen, and brain, indicating blood‐brain barrier penetration.^[^ ^496^ ^]^ Another study used inductively coupled plasma‐atomic emission spectroscopy (ICP‐AES) to assess the absorption, tissue distribution, and excretion of orally administered ZnO nanoparticles of different sizes in rats. The findings showed that ZnO nanoparticles were distributed mainly to the liver, lung, and kidney within 72 h, with most of the nanoparticles excreted via feces, and no significant size‐dependent differences were observed in distribution or excretion.^[^ ^497^ ^]^ Additionally, the blood kinetics and tissue distribution of silver nanoparticles of varying sizes (20, 80, and 110 nm) were investigated using ICP‐MS in rats following intravenous administration. The results indicated rapid clearance from the blood and widespread distribution across various organs, with size‐dependent accumulation patterns observed in the liver, lung, and spleen. Repeated administration led to significant accumulation in these organs, suggesting potential target sites for toxicity.^[^ ^498^ ^]^ ICP‐MS has also been used to assess the biodistribution and toxicity of dendrimer‐coated iron oxide nanoparticles (G4@IONPs).^[^ ^499^ ^]^

Moreover, molecular and cellular imaging, including single‐cell analysis, enhances the resolution and understanding of cellular mechanisms and behaviours in nano‐bio interactions. It enables detailed distribution analysis to assess how nanoparticles interact with various cells and identifies those without nanoparticles after treatment. In complex samples like blood or tissue, this approach can quantify and categorize different cell types and their specific interactions with nanoparticles, guiding design strategies for improved nanomedicine safety and efficacy. Additionally, imaging during single‐cell analysis adds value by correlating nanoparticle quantity with sub‐cellular accumulation.^[^ ^500^ ^]^ Additionally, live cell imaging has been utilized to assess whether nanoparticles are internalized and directed to specific cellular compartments. It also provides insights into the kinetics of these processes, examining variations in individual cells and how these variations relate to nanoparticle properties such as size.^[^ ^501^ ^]^ Computational modeling, such as physiologically based pharmacokinetic models, allows researchers to predict and analyze the systemic localization, target exposure, efficacy, and toxicity of nanoparticles in the body. These models simulate how nanoparticles distribute across different organs and tissues by dividing the body into compartments and applying mathematical equations to describe the transport processes. PBPK models help in understanding the interactions of nanoparticles with biological barriers, their passage through various compartments, and their eventual accumulation in target tissues.^[^ ^502^, ^503^ ^]^

As previously discussed, various approaches to studying nanotoxicity include 2D and 3D cell culture models, organ‐on‐a‐chip systems, organ cultures, and animal models. As the complexity of these models increases, they more accurately reflect human biology but also introduce challenges in assessing their state and managing numerous biological variables. Among these, 3D cell culture and organ‐on‐a‐chip models are particularly promising due to their ability to incorporate relevant cellular interactions and human genetics, though they are still in early development and not yet ready for clinical use. Traditional in vitro tests using immortalized cell lines often fail to mimic in vivo conditions, as these cells are cultured on plastic surfaces under conditions that do not replicate the physiological environment, leading to altered cell functions. Animal models remain the gold standard for toxicity testing, with various species such as mice, rats, zebrafish, rabbits, and Caenorhabditis elegans being used. However, even with diverse animal models, the understanding of nanotoxicity often remains superficial, as nanoparticles can accumulate in organs like the liver and spleen without causing immediate morphological changes. Additionally, the elimination of nanoparticles can be prolonged, sometimes extending up to 90 days. Moreover, research has shown that animal models with chronic diseases, such as bronchial asthma, may be more susceptible to nanoparticles, altering their distribution and increasing susceptibility due to the interplay between nanotoxicity and the disease's pathological mechanisms.^[^ ^504^ ^]^

### 5.2 GMP and Scale‐Up Considerations

The production of nanomaterials, particularly pharmaceutical‐grade nanotherapeutics, presents significant challenges in reproducibility and quality control. While there has been progress in the chemistry, manufacturing, and controls (CMC) of traditional nanoformulations like liposomes, emerging nanotherapeutics demand more extensive basic and translational research. Key considerations include selecting appropriate raw materials, developing standardized and scalable synthesis protocols, designing suitable batch sizes, performing stability checks, and employing analytical methods to assess physicochemical properties. Proper documentation is essential for maintaining quality across different chemical compositions.^[^ ^505^ ^]^

All drug products containing nanomaterials must comply with Current Good Manufacturing Practice (CGMP) as outlined in section 501(a)(2)(B) of the Federal Food, Drug, and Cosmetic (FD&C) Act. This includes adherence to CGMP regulations in 21 CFR parts 210, 211, and 212, and the applicable regulations in 21 CFR parts 600–680 for finished drug products, including over‐the‐counter (OTC) monograph drugs. As the use of nanomaterials in drug products expands, understanding their attributes and their impact on drug quality and manufacturing processes remains limited. Building a comprehensive knowledge base is crucial to understanding potential risks to product safety, identity, strength, quality, and purity. To develop robust control strategies and effective process validation protocols, continuous enhancement of the manufacturing process and control strategies is necessary. Leveraging manufacturing experience and insights into potential risks is vital for improving both processes and controls over time, ensuring the safety and efficacy of nanomaterial‐containing drug products. Nanomaterials, designed to achieve specific properties and clinical outcomes, are highly sensitive to production conditions and scales. Early establishment of controls is essential to prevent cross‐contamination and manage risks. Identifying Critical Quality Attributes (CQAs) early enables the design of effective in‐process controls. Manufacturing consistency is particularly critical in nanomedicine because even minor deviations in particle size distribution, drug loading, or surface properties can lead to significant variability in pharmacokinetics, efficacy, and immunogenicity. Inconsistent manufacturing may result in altered biodistribution profiles or therapeutic failure, necessitating stringent quality assurance at every step of the scale‐up. Variations in nanomaterial characteristics or impurities, such as incomplete surface coatings, can impact product consistency. Retaining samples from all batches is important for bridging developmental and commercial scales, ensuring that future analyses can confirm product safety and efficacy as methods and scales evolve^[^ ^506^ ^]^

Various methods are available for producing nanomedicines, with the emulsion‐based and nanoprecipitation methods being the most prominent for large‐scale production.^[^ ^507^, ^508^, ^509^ ^]^ These methods involve similar technical steps, such as introducing organic solvents into aqueous systems, solvent evaporation, stirring, and size reduction, which are also applicable to other fabrication techniques like solvent evaporation, gelation, and emulsion polymerization. Scaling up nanomedicine production involves integrating methods and transferring technology to industrial scales. Challenges include translating small‐scale processes to large‐scale production while ensuring quality, cost‐effectiveness, and timely product launches. Key challenges in scaling up include maintaining nanoparticle characteristics such as size, drug encapsulation, residual materials, colloidal stability, and surface morphology. Maintaining these parameters across multiple batches is essential for ensuring reproducibility and regulatory approval. Failure to uphold manufacturing consistency can result in clinical trial setbacks, increased regulatory scrutiny, and costly reformulations.

Process limitations also involve the stability of materials used and the presence of toxic solvents like chloroform or dichloromethane. Due to the harmful effects of these solvents, the pharmaceutical industry is moving away from their use, opting instead for aqueous coating methods. Therefore, developing novel methods using aqueous or less toxic solvents is crucial for producing nanomedicines.^[^ ^510^ ^]^ Continuous processing is becoming more common in the pharmaceutical industry to reduce batch‐to‐batch differences.^[^ ^511^ ^]^ Continuous synthesis has significant potential to produce nanoparticles with consistent and well‐controlled physicochemical properties. The FDA has requested pharmaceutical manufacturers to transition from batch to continuous processing by 2026. As a result, continuous chemical flow processes are gradually becoming the norm in pharmaceutical manufacturing, and this will likely set the new standard for large‐scale nanoparticle production.^[^ ^512^, ^513^, ^514^ ^]^

Continuous flow synthesis is increasingly used for commercial nanoparticle production in drug and vaccine delivery as an alternative to traditional batch manufacturing. This method offers numerous benefits, including shorter processing times, precise control over particle size and distribution, increased productivity, safer and more controlled reactions, and the ability to conduct complex chemistry that is difficult or impossible in batch processes.^[^ ^515^ ^]^ A microfluidic method has been developed for the controlled production of cadmium sulfide (CdS) nanoparticles. This approach offers advantages such as improved control over reaction conditions, leading to a reduction in nanoparticle polydispersity. The study demonstrates that by employing a continuous flow micromixer, nanoparticles with more uniform sizes can be synthesized efficiently, reducing the need for post‐synthesis processing like recrystallization. The findings suggest that microfluidic synthesis can be an effective alternative to bulk methods for the large‐scale production of nanoparticles with precise size control.^[^ ^516^ ^]^ Similarly, a flow manufacturing process has been reported for synthesizing Pd@Pt core‐shell nanoparticles, achieving high productivity and precise structural control.^[^ ^517^ ^]^ Further, a static microchannel T‐mixer was utilized for the continuous synthesis of nickel nanoparticles, resulting in smooth, spherical particles, with a focus on ensuring consistent quality and scalability for industrial applications.^[^ ^518^ ^]^

Importantly, maintaining manufacturing consistency throughout these processes requires robust real‐time analytical methods to monitor critical process parameters and ensure batch homogeneity. The integration of PAT tools allows for real‐time monitoring of critical parameters during production. This enables more precise control and immediate adjustments to maintain product quality throughout the manufacturing process. For instance, an inline size PAT tool, the NanoFlowSizer (patent pending), provided real‐time, non‐invasive measurement of nanoparticle size and particle size distribution. Utilizing Fourier Domain low coherence interferometry, it effectively addressed the limitations of traditional DLS by managing high turbidities and flow conditions, proving ideal for continuous monitoring in various production environments.^[^ ^519^ ^]^ Likewise, UV–vis spectroscopy was used in the Controlled Expansion of Supercritical Solutions (CESS) process to monitor API concentration in supercritical carbon dioxide (scCO2). This PAT tool, with a custom pressure‐rated flow‐through cell, enabled real‐time API quantification, improved process control, and supported Quality by Design (QbD) by optimizing solubilization parameters.^[^ ^520^ ^]^ Applying QbD principles involving the identification of CQAs early in the development process and designing robust processes with built‐in controls is useful. This approach enhances product quality and consistency by anticipating potential issues and addressing them proactively. Moreover, artificial intelligence and machine learning technologies are increasingly used to optimize manufacturing processes, predict potential issues, and improve the efficiency of scale‐up and quality control efforts. In nanomedicine, these advancements enhance drug delivery through nanoparticles and combinatorial nano‐therapy, while AI‐driven tools predict nanoparticle behavior and toxicity, aiming to revolutionize treatments with personalized approaches and reduce reliance on animal testing.^[^ ^521^, ^522^ ^]^

Overcoming the challenges of producing pharmaceutical‐grade nanotherapeutics requires a thoughtful approach that combines advances in manufacturing techniques with strong quality control measures. Adopting continuous flow synthesis and microfluidic methods represents a significant improvement over traditional batch processes. Incorporating automated, high‐precision monitoring systems, AI‐based predictive controls, and adaptive manufacturing platforms will be key to achieving consistent, reproducible, and scalable production of nanomedicines. By implementing effective control strategies, using safer solvents, and continuously refining production practices, the pharmaceutical industry can enhance the quality and effectiveness of nanotherapeutics.

### 5.3 Clinical Trials Design

As the field of nanomedicine progresses, the design of robust and well‐structured clinical trials becomes essential to ensure both safety and efficacy. A total of 409 clinical trials centered on therapies and diagnostics using nanomedicines have been identified. Since the beginning of 2018, over 247 new clinical trials (currently active or recruiting) have commenced.^[^ ^523^ ^]^ However, designing robust clinical trials tailored for nanomedicines requires careful consideration of their unique pharmacokinetic (PK) and pharmacodynamic (PD) profiles, as well as their complex interaction with biological systems. Unlike conventional drugs, nanomedicines often exhibit distinct distribution and clearance mechanisms, which may result in longer circulation times and the potential for bioaccumulation. This necessitates the development of tailored dosing regimens and delivery mechanisms to optimize therapeutic efficacy while minimizing side effects. Comprehensive safety assessments are particularly important in nanomedicine trials, as these therapies can introduce risks such as immunogenicity, genotoxicity, and unintended biodistribution to sensitive organs like the liver and brain. Including long‐term safety data to monitor delayed effects is essential for a thorough evaluation of these novel treatments.

Despite promising preclinical outcomes, a significant proportion of nanomedicine candidates fail during clinical trials due to a lack of in vivo efficacy. This disconnect between preclinical and clinical performance, often referred to as the “preclinical hype” problem, stems from limitations in current preclinical models that inadequately represent human physiological and pathological conditions. Nanoparticles that demonstrate tumor accumulation or cellular uptake in animal models frequently fail to replicate such effects in humans due to interspecies differences in immune response, tumor microenvironment, and vascular architecture.

Patient stratification is another critical aspect of trial design for nanomedicines. Biomarkers are increasingly being used to stratify patients based on genetic, metabolic, and immune profiles, enabling a more personalized approach to treatment. Identifying subgroups with specific susceptibility to nanoformulations, such as those with renal or hepatic impairment or a particular tumor microenvironment, can significantly improve treatment outcomes and safety. This approach aligns with the growing trend toward personalized medicine, where treatment plans are tailored to individual patient profiles. In such trials, real‐time patient monitoring and adaptive dosing strategies can help reduce toxicity while improving therapeutic efficacy, ensuring that the right dose is delivered to the right patient at the right time. Incorporating adaptive designs allows real‐time modifications of trials based on accumulating data. This flexibility enhances trial efficiency, making it possible to adjust sample sizes, terminate ineffective treatments early, or select promising subgroups.^[^ ^8^ ^]^ Adaptive trials are particularly useful in nanomedicine due to the variability in how different patients respond to nanoparticle‐based therapies. Examples include group sequential monitoring (allowing for early efficacy or futility stopping) and adaptive randomization, where patients are preferentially assigned to treatment arms showing better responses.^[^ ^524^ ^]^

Biomarker‐driven trials focus on selecting and enriching trial cohorts based on molecular profiles, with the aim of maximizing the likelihood that the selected patients will respond to therapy. Rapid advancements in molecular profiling (e.g., next‐generation sequencing) and drug discovery have created a demand for novel trial designs to evaluate biomarker‐defined populations efficiently. Biomarkers can be prognostic (related to disease outcome independent of treatment) or predictive (indicating response to specific treatments). Their integration into trials allows for personalized treatment strategies.^[^ ^525^ ^]^ The Biomarker Task Force, assigned by the National Cancer Institute (NCI) Investigational Drug Steering Committee (IDSC), was tasked with creating recommendations to enhance decision‐making regarding the inclusion of biomarker studies in early investigational drug trials.^[^ ^526^ ^]^ Integral biomarkers are essential for trial design and are typically required for patient enrollment or stratification. Integrated biomarkers are used to explore specific hypotheses but are not necessarily part of the trial's core eligibility criteria. Trials may involve a single biomarker that defines patient subgroups or multiple biomarkers, each corresponding to different therapeutic agents.^[^ ^527^ ^]^ Trials may involve a single biomarker that defines patient subgroups or multiple biomarkers, each corresponding to different therapeutic agents. Single‐integral biomarker designs include Enrichment Designs, which focus only on biomarker‐positive patients, Biomarker‐Stratified Designs, where both positive and negative patients are enrolled and randomized, and Biomarker‐Strategy Designs, which compare biomarker‐based treatments with standard care.^[^ ^528^ ^]^ For multiple biomarkers and disease types, Basket Trials test a single therapy across cancers with a common biomarker, while Umbrella Trials evaluate multiple therapies within a single cancer type, stratifying patients by biomarkers. Both are commonly used in master protocols for efficiency.^[^ ^529^, ^530^ ^]^

Real‐world evidence (RWE) can complement clinical trial data in health technology assessments and is becoming increasingly important in nanomedicine trials. While randomized clinical trials (RCTs) remain the gold standard for evaluating safety and efficacy, their strict criteria often limit the representativeness of patient populations. In contrast, real‐world studies using data from electronic health records and claims databases capture more diverse populations and provide long‐term safety and effectiveness data, especially for rare events. By supplementing traditional clinical trials, RWE offers a broader view of treatment effectiveness, utilization patterns, and health and economic outcomes in real‐world practice. RWE can provide valuable insights into how nanomedicine treatments perform across diverse patient populations outside the controlled environment of clinical trials.^[^ ^531^, ^532^ ^]^ Furthermore, implementing post‐market surveillance ensures the long‐term monitoring of nanomedicine safety and efficacy, offering a broader understanding of how these therapies behave in real‐world conditions.^[^ ^533^ ^]^ **Table** **5** outlines key recommendations for designing nanomedicine clinical trials, focusing on personalized approaches, safety, adaptive designs, and real‐world applicability.

**Table 5:** Key clinical trial design considerations for nanomedicines.

Clinical trial design aspect	Recommendations for nanomedicines
Unique PK/PD profiles	– Consider unique properties of nanomaterials, including altered distribution and clearance mechanisms. – Tailor dosing and delivery mechanisms to reflect longer circulation times and potential for bioaccumulation
Patient stratification	– Use biomarkers to stratify patients based on their genetic, metabolic, and immune profiles. – Identify subgroups with specific susceptibility to nanoformulations (e.g., renal, hepatic function, or tumor microenvironment)​.
Personalized medicine	– Tailor treatment plans to individual patient PK profiles to enhance therapeutic outcomes. – Utilize real‐time patient monitoring and adaptive dosing strategies to reduce toxicity while improving efficacy
Safety & efficacy endpoints	– Comprehensive safety assessment with endpoints like immunogenicity, genotoxicity, and biodistribution. – Include long‐term safety data to monitor potential delayed effects of nanomaterials on organs like liver and brain.
Biomarkers for monitoring	– Develop and validate specific biomarkers for early detection of treatment response and adverse reactions. – Utilize imaging and blood‐based markers to track nanoparticle localization and activity.
Adaptive trial designs	– Employ adaptive designs that allow for modifications based on interim results (e.g., dose adjustments or patient population refinement). – Integrate phase II/III trials to accelerate translation and reduce time to approval.
Real‐world evidence (RWE)	– Use RWE to supplement trial data, improving the relevance and applicability of clinical findings. – Implement post‐market surveillance to track the real‐world safety and efficacy of nanomedicine treatments.
Biological challenges	– Address biological barriers such as cellular uptake, nanoparticle aggregation, and immune system interactions. – Design trials to account for potential differences in nanoparticle behaviour in various disease conditions.

Addressing biological challenges, such as cellular uptake, nanoparticle aggregation, and immune system interactions, is crucial when designing trials for nanomedicines. These biological barriers can significantly influence the efficacy and safety of nanoparticle‐based therapies. Additionally, variations in nanoparticle behavior in different disease conditions, such as cancer or inflammatory diseases, must be taken into account when designing clinical trials. The inclusion of comprehensive safety and efficacy endpoints, as well as biomarkers for monitoring treatment response, helps to ensure the successful clinical translation of nanomedicines.

Notable case studies underscore these challenges:

- BIND‐014, a PSMA‐targeted docetaxel nanoparticle developed by BIND Therapeutics, showed promise in preclinical models due to its enhanced tumor accumulation via active targeting. However, Phase II trials revealed only modest improvements in efficacy over conventional docetaxel, leading to its eventual discontinuation and the company's bankruptcy. This highlights the limitation of relying solely on preclinical tumor models that may not reflect human tumor heterogeneity and microenvironment.
- CRLX101, a camptothecin‐loaded cyclodextrin‐based nanoparticle, initially demonstrated strong pharmacokinetic advantages in animal studies and early‐phase trials. Nevertheless, it failed to show statistically significant efficacy in later‐phase trials for renal and ovarian cancers, illustrating how promising drug release profiles and tumor penetration in preclinical settings do not always correlate with therapeutic success in diverse human populations.
- SGT‐53, a liposome‐based nanoparticle encapsulating a p53 gene construct, struggled with endosomal escape and effective gene transfection in humans despite compelling in vitro data. The inability to achieve sustained therapeutic levels in target tissues led to the early termination of trials.

These examples reinforce the need for improved preclinical models, better patient selection criteria, and adaptive trial designs that accommodate the complexities of nanoparticle behavior in vivo. Incorporating organ‐on‐a‐chip technologies, humanized models, and AI‐driven pharmacokinetic simulations may offer promising avenues to bridge the gap between preclinical hype and clinical performance. In summary, the unique challenges posed by nanomedicines require carefully structured clinical trials that incorporate personalized approaches, adaptive designs, and real‐world data to ensure safe and effective patient outcomes. Addressing the translational gap between laboratory findings and human biology is essential for improving the success rate of nanomedicine candidates in the clinic.

## 6 Regulatory Challenges and Gaps

As the field of nanomedicine continues to expand, it encounters a complex landscape of regulatory challenges that hinder its progress and potential impact on healthcare. These challenges arise from the rapidly evolving nature of nanotechnologies, which often outpace the current frameworks established by regulatory bodies worldwide. This section will explore the significant regulatory gaps and obstacles that must be addressed to facilitate the safe, effective, and accessible deployment of nanomedicine‐based products.

### 6.1 Lack of a Unified Set of Global Regulations

The primary regulatory challenge today stems from the absence of global standards for nanomedicine. Countries and regions develop their own sets of rules, creating a varied and often contradictory landscape of requirements. For example, while the FDA may focus on certain types of toxicological assessments, the EMA and regulators in Asia might prioritize different aspects.^[^ ^534^, ^535^ ^]^ This inconsistency forces companies to navigate a complex maze of differing regulatory processes and standards. Such variations not only complicate clinical trials and product approvals but also delay the introduction of new therapies as companies must satisfy each region's unique requirements, often duplicating efforts in studies and documentation.^[^ ^536^ ^]^ Moreover, the cost of adhering to multiple regulatory frameworks can be prohibitive, particularly for smaller entities or academic institutions with fewer resources. This regulatory fragmentation can discourage innovation, with developers potentially shying away from exploring valuable nanomedical advancements due to uncertain regulatory landscapes.^[^ ^537^ ^]^

Adopting a unified international regulatory framework would greatly streamline the development of nanomedicines as it would allow developers to follow a single, comprehensive set of standards across all stages of development, from clinical trials to manufacturing and quality control.^[^ ^538^ ^]^ This would not only speed up the review and approval processes, providing quicker access to new treatments for patients, but also enhance the global distribution of these therapies. With more predictable regulatory requirements, companies might be more inclined to invest in pioneering nanotechnologies, possibly leading to significant advances in treatment effectiveness and safety.^[^ ^539^ ^]^

### 6.2 Environmental Concerns

The rapid development and widespread commercialization of nanomedicines have heralded notable advances in precise drug targeting and minimized systemic toxicity. However, the environmental consequences associated with these technologies during their lifecycle remain underexplored and inadequately addressed by existing regulations.^[^ ^540^ ^]^ This gap is evident from production to disposal, where environmental impacts are often overlooked.

Nanoparticle production frequently utilizes chemicals and processes detrimental to the environment. Current regulations fall short in managing the release of these substances into air, water, and soil. For example, the prevalent use of solvents and volatile organic compounds (VOCs) in nanoparticle synthesis sees a lack of consistent emission controls and waste management standards across different regions.^[^ ^541^ ^]^ Once administered, nanomedicines can enter the environment through patient excreta and other waste, yet there is limited guidance on their nano‐scale interactions with ecosystems, where they may disrupt natural processes in ways traditional toxicity tests do not capture. The disposal of nanomedicines also presents considerable challenges. Standard disposal methods for medical waste might not be appropriate for materials containing nanoparticles, which tend to persist and accumulate in the environment. The absence of specific disposal regulations for nanomedical waste can lead to environmental pollution.^[^ ^542^ ^]^

To bridge these regulatory gaps, it is essential for regulatory bodies to implement environmental risk assessments tailored to nanomedicines. There is a need for comprehensive studies that span the entire lifecycle of nanomedicines, pinpointing key intervention points to mitigate environmental damage.^[^ ^543^ ^]^ Developing and standardizing nano‐specific toxicology protocols is also crucial. These protocols, which should cover various environmental domains such as aquatic, terrestrial, and atmospheric, will aid in understanding the specific impacts of nanoparticles. Moreover, mandating regular environmental monitoring and reporting for nanomedicine manufacturers and users will enable continuous tracking of potential environmental releases and their effects, promoting responsible management throughout the lifecycle of these products.^[^ ^544^ ^]^

Incorporating green chemistry and green engineering principles into the design and manufacturing of nanomedicines can significantly mitigate their environmental impacts. These principles advocate for the minimization of hazardous substances by using safer materials and solvents during the synthesis of nanoparticles, which reduces toxicity and environmental harm. Additionally, optimizing manufacturing processes to be energy‐efficient helps minimize waste production.^[^ ^545^ ^]^ Another crucial aspect is sustainable lifecycle management, which involves designing nanomedicines and their packaging to facilitate easier degradation or recycling. This approach not only reduces long‐term environmental impacts but also aligns with broader sustainability goals, enhancing the ecological compatibility of nanomedical technologies.^[^ ^546^ ^]^

### 6.3 Intellectual Property Issues

Intellectual property (IP) protection plays a pivotal role in the field of nanomedicine, serving both as an incentive for innovation and as a potential barrier to entry. Given the wide‐ranging and interdisciplinary nature of nanotechnologies, many patents often overlap in scope, creating a “patent thicket.” This complex web of interlocking patents can obstruct new entrants, hampering innovation by complicating the navigation of the IP landscape without violating existing patents.^[^ ^547^ ^]^ Patents in nanotechnology are frequently drafted with broad claims encompassing various uses, compositions, and formulations, which can be overly expansive, sometimes even covering whole research or application domains, thus deterring investment in technologies that could potentially infringe these extensive claims.^[^ ^548^ ^]^

To effectively address the challenges within the IP landscape of nanomedicine and promote innovation, several strategic solutions can be implemented. One such strategy is the formation of patent pools, which involve multiple patent holders agreeing to cross‐license patents related to a specific technology.^[^ ^549^, ^550^ ^]^ This method is particularly beneficial in fields with prevalent overlapping technologies, such as nanoparticle‐based drug delivery systems. Patent pools can significantly reduce litigation risks and transaction costs, while also fostering collaborative innovation, thereby allowing companies to concentrate on progress rather than the complexities of IP navigation.^[^ ^551^ ^]^

Additionally, imposing more stringent patentability criteria could help refine the scope of what is considered a patentable nanotechnology invention. Regulatory bodies and patent offices could enforce stricter requirements for demonstrating novelty and non‐obviousness, coupled with more defined claims to prevent the issuance of excessively broad patents that encompass entire research areas. Such measures would help mitigate the problem of broad patent scopes that can stifle further research and development. Enhancing the patent examination process is also crucial. By providing patent examiners with access to improved technical expertise and databases, and possibly offering specialized training in nanotechnology, patent offices can enhance the accuracy with which they assess the novelty and scope of nanotechnology patents.^[^ ^552^ ^]^

### 6.4 Economic and Market Access Barriers

The economic barriers in the field of nanomedicine are significant, particularly for new entrants who are faced with the daunting costs of R&D and regulatory compliance. The inherent technical specificity of nanomedicines often necessitates substantial upfront investment in cutting‐edge research facilities, specialized equipment, and highly skilled personnel. This initial high capital requirement forms a formidable barrier to entry, particularly for startups and smaller companies without substantial financial backing.^[^ ^553^ ^]^

As discussed, the synthesis of nanoparticles often involves high‐resolution instrumentation such as electron microscopes and atomic force microscopes, which are both expensive and require specialized operational expertise. Furthermore, the characterization of these particles demands advanced techniques like DLS for size distribution analysis and zeta potential measurements to assess surface charge, which are critical factors that influence biodistribution and cellular uptake of nanoparticles.^[^ ^554^ ^]^ Techniques such as nanoprecipitation, liposome extrusion, or polymeric nanoparticle fabrication require precise control over variables like solvent quality, shear forces, and temperature, all of which necessitate high‐end equipment and specialized facilities. Scaling these processes from lab to industrial production while maintaining batch consistency and product stability is both technically demanding and financially burdensome.^[^ ^555^ ^]^

The high costs incurred during the development and approval phases naturally drive up the market price of nanomedicines. This poses an ethical dilemma: how to recover these substantial costs while ensuring that these innovative treatments remain affordable and accessible to patients. The issue of affordability is particularly pressing in the context of life‐saving technologies, where patient access can literally mean the difference between life and death. The challenge is to balance cost recovery with ethical responsibilities, ensuring that financial gains do not come at the expense of patient care and accessibility.^[^ ^556^ ^]^

One viable solution to these economic barriers is the establishment of government or public‐private partnerships. These collaborations can provide critical financial support through funding and subsidies, helping to alleviate the burden of R&D and regulatory costs. For instance, government grants and subsidies could be specifically earmarked for early‐stage research or for covering costs associated with clinical trials.^[^ ^557^ ^]^ Additionally, public‐private partnerships can leverage resources from both sectors, combining public funding and oversight with the innovation and agility of private entities.^[^ ^558^, ^559^ ^]^

### 6.5 Data Requirements and Privacy Issues

The complex data requirements for the regulatory approval of nanomedicines pose significant privacy concerns, particularly when linked to patient‐specific information. The granularity of this data, essential for assessing the safety and effectiveness of nanomedicines, could inadvertently expose highly sensitive personal health information.^[^ ^560^ ^]^ Managing patient data privacy involves several key challenges. First, data security is paramount, as it involves protecting health data against unauthorized access and breaches and ensuring that sensitive information remains confidential. Second, data minimization plays a critical role; it involves collecting only the data that is absolutely necessary for specific regulatory or research purposes, thereby mitigating potential privacy risks.^[^ ^561^ ^]^ Lastly, effective consent management ensures that patients are fully informed about how their data will be used, stored, and shared, thereby respecting their privacy and autonomy. This approach not only safeguards the information but also helps in maintaining trust in the evolving field of nanomedicine.^[^ ^562^ ^]^

To effectively address these data management challenges and privacy issues, robust data management frameworks coupled with enhanced cybersecurity measures are critical. First, it is essential to establish standardized protocols for data collection, processing, and storage that are specifically tailored to the sensitive nature of nanomedicine‐related data.^[^ ^563^ ^]^ Policies that enforce data minimization and purpose limitation should also be implemented to ensure that data is not used beyond its intended scope. In terms of security measures, advanced encryption techniques should be utilized to protect data at rest and in transit, safeguarding against breaches and unauthorized access.^[^ ^564^ ^]^ Additionally, the employment of blockchain technologies can enhance the security and traceability of data transactions within the nanomedicine research and regulatory network, providing a more secure and transparent system.^[^ ^565^ ^]^

Regular security audits and compliance checks are imperative to ensure that data handling practices adhere to the highest standards set by data protection laws such as the GDPR or HIPAA. These practices should be regularly updated and refined to keep pace with technological advancements and emerging threats.^[^ ^566^ ^]^ Lastly, implementing ongoing training and awareness programs for all stakeholders involved in handling nanomedicine data is vital. These programs should focus on raising awareness about the best practices in data privacy and security, ensuring that everyone is informed and equipped to manage data responsibly.^[^ ^567^ ^]^

### 6.6 Transparency and Public Trust

The integration of nanomedicines into clinical and consumer settings is accompanied by a significant trust gap, primarily due to the complex, technical nature of nanotechnology and a lack of transparent information. Public skepticism often stems from concerns about safety, long‐term effects, and ethical implications. Moreover, the rapid pace of development in nanotechnology can overwhelm traditional communication channels, leaving the public with outdated or incomplete information about the risks and benefits of nanomedicines.^[^ ^568^ ^]^ The complexities of nanomedicine are not easily understood without specialized knowledge, which can make transparency challenging. In many cases, the mechanisms of action, potential side effects, and environmental impacts are not communicated effectively to non‐experts. This issue is compounded by sensationalized media reports that can distort public perception, emphasizing potential dangers without equally highlighting the benefits.^[^ ^569^ ^]^

Building public trust in nanomedicine requires strategic initiatives focused on clear, accessible communication and robust engagement with both patients and the broader community. A key component is simplifying the science; this involves breaking down complex scientific concepts into understandable language and using visual aids like infographics and videos to explain the workings and potential impacts of nanomedicines. Additionally, it's crucial to ensure balanced reporting that acknowledges both the potential benefits and the associated risks and uncertainties of nanomedicine. This approach can help prevent misinformation and alleviate fears stemming from unknowns.^[^ ^570^ ^]^

Patient engagement is another vital area. Developing materials and programs specifically designed to educate patients and the public about nanomedicine treatments is essential. These resources might include patient forums, workshops, and interactive web platforms, all aimed at increasing knowledge and understanding.^[^ ^571^ ^]^ Moreover, including patient advocacy groups in the development and review processes of nanomedicines ensures that patient concerns and needs are respected and integrated, further building trust.^[^ ^572^ ^]^ Transparency in regulatory processes is also critical. Publicizing clear, detailed regulatory roadmaps that outline the pathway from nanomedicine development to approval, emphasizing the stringent safety and efficacy standards required, helps demystify the approval process. Openness about the challenges of regulating emerging technologies is also necessary; regular updates on new findings, ongoing research, and changes in regulatory standards can maintain public trust and engagement.^[^ ^573^ ^]^

## 7 Concluding Remarks

In this review, we have systematically explored the evolving interface between nanomedicine and the regulatory and commercial domains that influence its clinical application. Beginning with the classification of nanoparticle systems and extending to the assessment of globally approved products, we have also analyzed regulatory frameworks that shape the path from laboratory innovation to clinical implementation. This comprehensive overview highlights both the progress and the persistent obstacles that continue to affect the clinical translation of nanomedicines.

The rapid advancement of nanotechnology in medicine presents unique challenges, particularly in the context of regulatory science. Existing regulatory paradigms often rely on traditional pharmaceutical frameworks that do not fully accommodate the nano‐specific properties of modern therapeutics. These include quantum effects, increased surface area to volume ratios, and unpredictable biodistribution and clearance mechanisms. As a result, regulatory policies frequently lag behind the pace of innovation. Bridging this gap requires a shift toward adaptive regulatory frameworks that can rapidly integrate scientific and technological breakthroughs into policy‐making. A central component of this shift involves greater investment in foundational research to better understand how nanoparticles interact with biological systems at the molecular, cellular, and tissue levels. Predictive models of toxicity,^[^ ^574^ ^]^ immune reactivity,^[^ ^575^ ^]^ and long‐term biodistribution^[^ ^576^ ^]^ must be developed using physiologically relevant systems, including humanized animal models and advanced in vitro platforms.^[^ ^503^, ^577^ ^]^ Such efforts will enable more accurate preclinical screening and risk assessment for emerging nanotherapeutics.

Fostering a collaborative environment that integrates academic researchers, industry partners, and regulatory authorities is essential. This collaboration should focus on harmonizing nanoparticle characterization methods, setting universal benchmarks for safety and efficacy, and facilitating shared access to high‐quality data. Regulatory convergence at the international level would further reduce duplication of efforts and expedite cross‐border approvals.

The emergence of personalized nanomedicine introduces another layer of complexity. These therapies, often customized for individual patients using genetic, proteomic, or metabolic data, challenge conventional regulatory concepts of batch uniformity and reproducibility.^[^ ^578^ ^]^ Personalized formulations may be manufactured at micro‐scale volumes, making traditional quality control testing and pharmacovigilance protocols difficult to implement. Future regulatory frameworks must be designed to evaluate such products using flexible, risk‐based approaches that ensure patient safety without imposing impractical constraints on innovation.^[^ ^579^ ^]^ Moreover, personalized nanomedicines frequently rely on sensitive personal health information, particularly genetic data. This necessitates strict regulatory oversight regarding data privacy, ownership, and ethical use. Regulatory strategies must include robust cybersecurity measures, consent procedures, and cross‐jurisdictional data governance models that safeguard patient rights while enabling scientific progress.^[^ ^580^ ^]^

Looking ahead, several key research and development areas warrant focused attention. Artificial intelligence and machine learning algorithms have shown tremendous potential in accelerating the design and optimization of nanoparticle formulations.^[^ ^581^, ^582^ ^]^ These tools can be employed to predict physicochemical properties, simulate biodistribution, and forecast potential toxicological outcomes using large datasets derived from experimental and clinical studies.^[^ ^583^ ^]^ AI‐driven platforms may also improve patient stratification by identifying nanoparticle‐responsive subgroups based on omics‐derived biomarkers.^[^ ^584^ ^]^ Digital twin technologies represent another promising avenue. These are computational models of individual patients that integrate real‐time physiological, pathological, and treatment data. Digital twins could be used to test the therapeutic impact of different nanoparticle formulations in silico before actual administration, improving both safety and efficacy while reducing reliance on empirical trial‐and‐error methods.^[^ ^585^, ^586^ ^]^ When integrated with clinical decision support systems, these technologies may also guide dosing regimens, monitor treatment progress, and adapt therapeutic strategies in real time.^[^ ^587^ ^]^

The use of multi‐omics platforms, including genomics, transcriptomics, proteomics, and metabolomics, offers further opportunities to decode the complex interactions between nanoparticles and the human body. These tools may help identify off‐target effects, elucidate mechanisms of cellular uptake, and discover predictive biomarkers that guide both formulation design and patient selection.^[^ ^588^ ^]^ On the regulatory front, emerging technologies such as Process Analytical Technology (PAT) and Quality by Design (QbD) are becoming integral to ensuring product consistency and quality during manufacturing.^[^ ^589^, ^590^ ^]^ Implementation of these tools can reduce batch‐to‐batch variability and facilitate regulatory compliance, particularly for personalized or complex nanomedicine products.

There is also growing interest in the concept of regulatory sandbox frameworks. These are controlled environments where novel therapeutics can be evaluated under flexible regulatory conditions while maintaining safety oversight. Such frameworks could be particularly valuable for evaluating unconventional nanomedicine platforms, including gene‐editing nanoparticles or stimuli‐responsive systems, that fall outside traditional regulatory definitions.^[^ ^591^, ^592^ ^]^ Finally, the international harmonization of terminology, testing protocols, and classification systems for nanomedicine is urgently needed. Current discrepancies between regulatory agencies in different regions often delay product approvals and create barriers for global commercialization. Establishing unified standards through interagency collaboration, such as that fostered by the International Council for Harmonization, will be critical to aligning global regulatory practices.

In conclusion, this review underscores the multifaceted challenges and opportunities at the intersection of nanomedicine innovation and regulatory evolution. Addressing these issues will require a multidimensional strategy that integrates technological advancement, regulatory flexibility, and cross‐sector collaboration. As nanomedicine continues to mature, targeted efforts in emerging research areas and progressive regulatory planning will be essential to ensuring that its full therapeutic potential can be realized efficiently, safely, and equitably.

## Conflict of Interest

The authors declare no conflict of interest.

## Author Contributions

N.D.: Conceptualization, Writing – Original Draft, Writing – Review & Editing; D.R.: Writing – Original Draft; M.P.: Writing – Original Draft; N.B.: Writing – Original Draft; R.P.: Writing – Review & Editing; L.K.V.: Conceptualization, Writing – Review & Editing, Supervision, Project Administration.
